# Zinc(II) Terpyridine Complexes: Substituent Effect on Photoluminescence, Antiproliferative Activity, and DNA Interaction

**DOI:** 10.3390/molecules24244519

**Published:** 2019-12-10

**Authors:** Jiahe Li, Rongping Liu, Jinzhang Jiang, Xing Liang, Ling Huang, Gang Huang, Hailan Chen, Lixia Pan, Zhen Ma

**Affiliations:** 1 School of Chemistry and Chemical Engineering, Guangxi University, Nanning 530004, Guangxi, China; lijiahe006@163.com (J.L.); 18877571207@163.com (R.L.); jjz19941028@gmail.com (J.J.); liangxing587@126.com (X.L.); linghuanglinghuang@163.com (L.H.); 2 Centro de Química Estrutural, Instituto Superior Técnico, Universidade de Lisboa, Av. Rovisco Pais, 1049-001 Lisbon, Portugal; 3 National Engineering Research Center for Non-Food Biorefinery, State Key Laboratory of Non-Food Biomass and Enzyme Technology, Guangxi Academy of Sciences, Nanning 530004, Guangxi, China; wangyi.07@163.com; 4 School of Animal Science and Technology, Guangxi University, Nanning 530004, Guangxi, China

**Keywords:** terpyridine complex, photoluminescence, anti-tumor activity, DNA interaction, molecular docking

## Abstract

A series of ZnCl_2_ complexes (compounds **1**–**10**) with 4′-(substituted-phenyl)-2,2′:6′,2′′-terpyridine that bears hydrogen (**L^1^**), *p*-methyl (**L^2^**), *p*-methoxy (**L^3^**), *p*-phenyl (**L^4^**), *p*-tolyl (**L^5^**), *p*-hydroxyl (**L^6^**), *m*-hydroxyl (**L^7^**), *o*-hydroxyl (**L^8^**), *p*-carboxyl (**L^9^**), or *p*-methylsulfonyl (**L^10^**) were prepared and then characterized by ^1^H NMR, electrospray mass-spectra (ESI-MS), IR, elemental analysis, and single crystal X-ray diffraction. In vitro cytotoxicity assay was used to monitor the antiproliferative activities against tumor cells. Absorption spectroscopy, fluorescence titration, circular dichroism spectroscopy, and molecular modeling studied the DNA interactions. All of the compounds display interesting photoluminescent properties and different maximal emission peaks due to the difference of the substituent groups. The cell viability studies indicate that the compounds have excellent antiproliferative activity against four human carcinoma cell lines, A549, Bel-7402, MCF-7, and Eca-109, with the lowest IC_50_ values of 0.33 (**10**), 0.66 (**6**), 0.37 (**7**), and 1.05 (**7**) μM, respectively. The spectrophotometric results reveal that the compounds have strong affinity binding with DNA as intercalator and induce DNA conformational transition. Molecular docking studies indicate that the binding is contributed by the π…π stacking and hydrogen bonds, providing an order of nucleotide sequence binding selectivity as ATGC > ATAT > GCGC. These compounds intercalate into the base pairs of the DNA of the tumor cells to affect their replication and transcription, and the process is supposed to play an important role in the anticancer mechanism.

## 1. Introduction

Cancer is the second leading cause of global death and it is responsible for an estimated 9.6 million loss of human lives in 2018. The Pt-based drugs, such as cisplatin and carboplatin, are widely used as anticancer agents. However, these platinum complexes display severe side effects, such as nephrotoxicity, neurotoxicity, and ototoxicity, which limit their applications [1,2,3,4]. Generally, molecules can interact with DNA to affect its replication and transcription, leading to cell death and apoptosis [5,6,7]. Meanwhile, non-covalent interaction of metal complexes with DNA has shown the importance in recognition of different DNA conformations (B-, A-, and Z-forms) and binding site selectivity (minor or major groove, mismatched nucleobase sites, and bulge loops, etc.) as well as the nucleobase sequence recognition [8,9,10,11,12,13,14,15,16,17]. One treatment might be effective that combines a metal complex as chemotherapeutic agent with a photosensitizer and followed by the laser destruction of cancer using photodynamic therapy (PDT) [18,19]. Accordingly, the development of metal complexes that have DNA sequence-selective and cleavage potential is essential for further expected applications in molecular biology, medicine, and related fields [20,21].

2,2′:6′,2′′-terpyridine (TPY) has been recognized as a useful ligand for transition metal and rare earth metal ions in inorganic chemistry [22,23,24,25,26,27]. The terpyridine molecule serves as a tridentate, nearly coplanar, N_3_ donor ligand, and, in some cases, also as a mono or bidentate ligand [28]. Square planar and octahedral metal complexes with TPY ligands have been reported to be of great biological interest, and complexes exhibiting planar structures can easily insert themselves in DNA base pairs [29,30,31]. Zinc, being one of the most important trace metals for human being, appears as an attractive metal due to its importance in the regulation of the metabolism of cells, cytoprotective effect, and suppressing the apoptotic pathways [32,33,34,35,36]. Zinc TPY complexes are good candidates for photo-activated processes and they can be used in PDT [37,38,39]. Therefore, the synthesis of new zinc TPY complexes, study of their pharmacological properties, and screening as anti-tumor agents constitute matters of current interest.

As one part of our ongoing interests in designing new substituted TPYs and their metal complexes, ten ZnCl_2_ complexes with 4′-phenyl-2,2′:6′,2′′-terpyridine (**L^1^**), 4′-(4-methyl-phenyl)-2,2′:6′,2′′-terpyridine (**L^2^**), 4′-(4-methoxyl-phenyl)-2,2′:6′,2′′-terpyridine (**L^3^**), 4′-(4-phenyl-phenyl)-2,2′:6′,2′′-terpyridine (**L^4^**), 4′-(4-methylphenyl-phenyl)-2,2′:6′,2′′-terpyridine (**L^5^**), 4′-(4-hydroxyl-phenyl)-2,2′:6′,2′′-terpyridine (**L^6^**), 4′-(3-hydroxyl-phenyl)-2,2′:6′,2′′-terpyridine (**L^7^**), 4′-(2-hydroxyl-phenyl)-2,2′:6′,2′′-terpyridine (**L^8^**), 4′-(4-carboxyl-phenyl)-2,2′:6′,2′′-terpyridine (**L^9^**), and 4′-(4-methylsulfonyl-phenyl)-2,2′:6′,2′′-terpyridine (**L^10^**) were synthesized. The photoluminescent properties of the zinc(II) complexes were determined and the antiproliferative activities of the ten complexes against A549, Bel-7402, MCF-7, and Eca-109 cancer cell lines were tested. The interaction of the complexes with DNA was studied while using multiple spectrophotometric methods. Molecular modeling was carried out to further explore the binding selectivity among various duplex oligonucleotides to explore the nature of their anticancer mechanism. Finally, the effect of the substituents at the 4′-position of zinc TPY complexes on photoluminescence, antiproliferative activity, and DNA interaction has been studied.

## 2. Results and discussion

### 2.1. Synthesis and Characterization

**Scheme 1:**
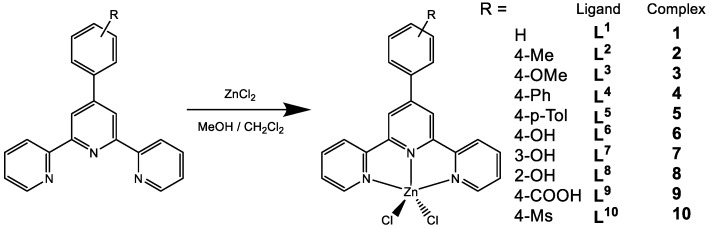
Syntheses of compounds **1**–**10.**

4′-phenyl-terpyridine (**L^1^**), 4′-(4-hydroxyl-phenyl)-2,2′:6′,2′′-terpyridine (**L^6^**), 4′-(3-hydroxyl-phenyl)-2,2′:6′,2′′-terpyridine (**L^7^**), and 4′-(2-hydroxyl-phenyl)-2,2′:6′,2′′-terpyridine (**L^8^**) were obtained following the literature procedures [40,41,42], and **L^2^**–**L^5^**, **L^9^,** and **L^10^** were obtained by the revised procedure through the condensation of the respective substituted benzaldehydes and 2-acetylpyridine in a sodium hydroxide aqueous solution, and then a refluxed procedure in ethanol in the presence of ammonium acetate by the yields in the range of 30–70%. The Zn(II) complexes [Zn(Cl)_2_L^n^] (**1**–**10**, n = 1–10) were synthesized (Scheme 1) by a reaction of **L^1^**–**L^10^** with ZnCl_2_, giving the yields in the range of 70–85%. Compounds **1**, **6**, **7**, and **8** have already been reported [41,42].

As shown in Figures S1–S6, all of the ^1^H NMR spectra show the expected seven doublet or triplet signals between δ 7.2 and 9.3 due to the fourteen aromatic protons on TPY; moreover, compounds **4** and **5** also show aromatic proton signals of the phenyl group. The methyl proton signals of compounds **2**, **3**, **5,** and **10** appear at 2.44, 3.88, 2.39, and 3.36 ppm in high field region, respectively. For compound **9**, the signal of the carboxyl proton occurs as a broad singlet at δ 13.31. Figures S7–S12 show the electrospray mass-spectra (ESI-MS) of compounds **2**–**5**, **9,** and **10** and the most intense peak for each compound is recognized as m/z of [ZnClL^2^]^+^, [ZnClL^3^]^+^, [ZnClL^4^]^+^, [ZnClL^5^]^+^, [L^9^+H^+^]^+^, or [ZnClL^10^]^+^ at 422, 438, 488, 498, 355, and 486, respectively. In the IR spectra of the compounds (Figures S13–S18), the multiple bands in the range of 1425–1615 cm^−1^ are observed for the C=C stretch, and the bands in the range of 1000–1300 cm^−1^ for C–H bending. For compounds **2** and **5**, the bands at 2922 and 2853 cm^−1^ confirm the presence of a methyl group. Similarly, the bands at 2919 and 2840 cm^−1^ prove the methyl group in compound **5**. The band of methoxyl appears at 2841 cm^−1^ in the electrospray mass-spectra (ESI-MS) of compounds **2**–**5**, **9,** and **10**. A broad band in a range of 3300–3600 cm^−1^ and sharp band at 1712 cm^−1^ are due to the carboxyl group of compound **9**. The sulfonyl of compound **10** shows strong SO_2_ stretching bands at 1290 and 1141 cm^−1^, and strong SO_2_ bending bands at 553 and 532 cm^−1^.

### 2.2. Single Crystal X-Ray Crystallography

**Figure 1:**
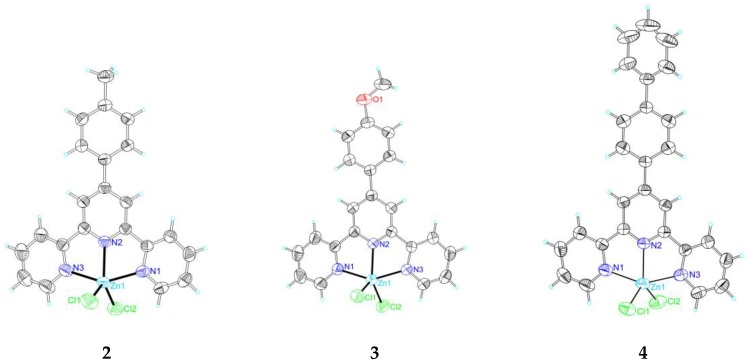

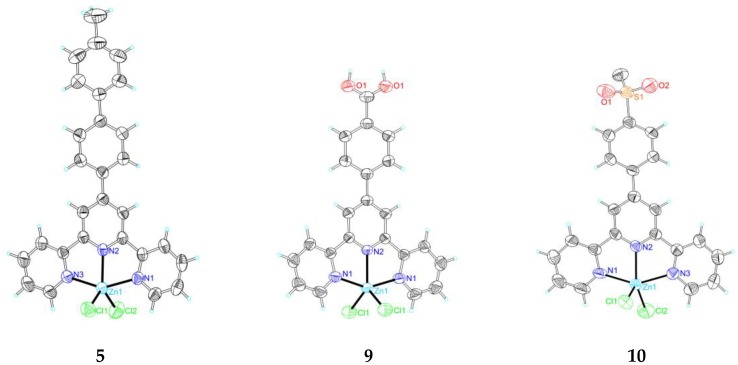
Thermal ellipsoid plot, drawn at the 60% probability level, of [Zn(Cl)_2_L^2^] (**2**), [Zn(Cl)_2_L^3^] (**3**), [Zn(Cl)_2_L^4^] (**4**), [Zn(Cl)_2_L^5^] (**5**), [Zn(Cl)_2_L^9^] (**9**), and [Zn(Cl)_2_L^10^] (**10**) with atomic numbering scheme.

The crystal structures of [Zn(Cl)_2_L^1^] (**1**), [Zn(Cl)_2_L^6^] (**6**), [Zn(Cl)_2_L^7^] (**7**), and [Zn(Cl)_2_L^8^] (**8**) have been already reported [39,40]. The single crystal X-ray crystallography of **2**–**5, 9**, and **10** confirms their formulation as the pentacoordinated [Zn(Cl)_2_L], and the zinc cation presents the common square pyramidal geometry. Figure 1 shows thermal ellipsoid plots of compounds **2**–**5, 9,** and **10** a, and crystal data are listed in Table 1.

Compound **2** is a mononuclear neutral zinc complex (Figure 1). Each zinc ion is coordinated by the three N atoms of the 4′-(4-methyl-phenyl)-2,2′:6′,2′′-terpyridine (L) ligand, and two chloride atoms as two auxiliary ligands, which therefore form an irregular square based pyramid with a N_3_Cl_2_ coordination environment with the two terminal coordinated nitrogen atoms of the ligand occupying the apical positions (bond angles range from 74.36(5) to 98.87(4)° between the apical atoms and the equatorial positions). This type of geometry is that usually reported for related Zn(II) compounds, e.g., [Zn_2_Cl_2_L] (L = 4′-phenyl-2,2′:6′,2′′-terpyridyl) [41,42] or [Zn(4′-MeSterpy)Cl_2_] (4′-MeSterpy = 4′-methylthio-2,2′:6′,2″-terpyridyl) [43]. In its structure, the terpy group in the ligand is nearly planar (presenting an RMS deviation of 0.0787 Å with the dihedral angle between the three pyridyl planes being 1.09, 8.50, and 8.94°, respectively). Moreover, the tolyl ring is twisted (29.21(8)°) relative to the plane containing N1, N2, N3, and Zn1 or (29.89(8)°) with the connecting pyridyl ring. No hydrogen bond is detected in the structure. The complex molecules present two kinds of intermolecular π–π interactions due to the specific arrangements of the ligands in **2**, including one between the two pyridyl rings of the ligand and the other between the ring formed by Zn–N1–C5–C6–N2 and one terminal pyridyl ring with the centroid distances of 3.5153(2) and 3.8005(2) Å. The structure of **2** shows one kind of π–ring (Y–H...Cg) interaction between one hydrogen at C20 and a neighboring terminal pyridyl ring of the ligand with an atom-centroid distance (X...Cg) of 3.5060(2) Å.

The three pyridyl units of L in these complexes are planar (with an RMS deviation of 0.1218 Å in **3**, 0.0333 Å in **4**, 0.0211 Å in **5**, 0.0427 in **9**, and 0.0452 in **10**). The angles between the pendent phenyl rings, the connected pyridyls, and the attached N–N–N–Zn planes are 36.88(6)° and 38.09(6)° in **3**, 37.19(17)° and 35.55(16)° in **4**, 30.96(6)° and 30.97(6)° in **5**, 34.15(18)° and 35.83(16)° in **9**, and 17.44(21)° and 21.32(18)° in **10**, respectively, showing that the hydroxyl substituent has no significant effect on these angles. No any hydrogen bond is detected in the structures of **3** and **4** and no classic hydrogen bond is found in the structure of **5**, but two other kinds of intermolecular hydrogen bonds exist in its structure, which involve the oxygen O1 of the carboxyl group at the ligand and one chloride atom Cl1 and the hydrogens at the carbon atoms of the terpyridine ligand (C3 and C4). Intermolecular hydrogen bonds exist in the structures of **9** and **10**, which are in the range of 2.50–3.60 Å for **9** and 2.90–3.50 Å for **10** (including one intramolecular hydrogen bond between the oxygen (O2) of the sulfuryl group and the hydrogen at the carbon atom of the terpyridine ligand (C18).

Several π–ring interactions exist in each structure of complexes **3**, **4,** and **5**, but no such interaction in **9** and **10**, between H atoms at the ligands and their neighboring rings. The structure of **3** presents one kind of π–ring (Y–H…Cg) interaction between one hydrogen at C14 and a neighboring middle pyridyl ring of the ligand with an atom-centroid distance (X…Cg) of 3.6948(8) Å. In **4**, there are two kinds of π–ring (Y–X…Cg) interactions between one hydrogen at C3 and two neighboring phenyl rings of the ligand with the same atom-centroid distances (X…Cg) of 3.5793(6) Å. For **5**, one interaction is 3.6734(4) Å for one H at C28 and its neighboring ring constituted by Zn1–N2–C10–C11–N3, and no such interaction is observed in **9** and **10**.

In these structures, the different packing patterns lead to the different π–π stackings: one (with distance being 3.5501(6) Å, which involves two pyridyl rings), five (in the range of 3.50–3.93 Å, involving the ring formed by Zn1–N2–C5a–C6a–N1a, two pyridyl rings and two phenyl rings), four (in the range of 3.67–3.88 Å, involving the rings formed by Zn1–N1–C5–C6–N2 and Zn1–N1–C5–C6–N2 and two pyridyl rings), and one (3.6220(2) Å, involving two pyridyl rings) π–π stacking are observed in **4**, **5**, **7**, and **10**, respectively. No such stacking is presented in **3**.

### 2.3. Photoluminescent Properties

**Figure 2:**
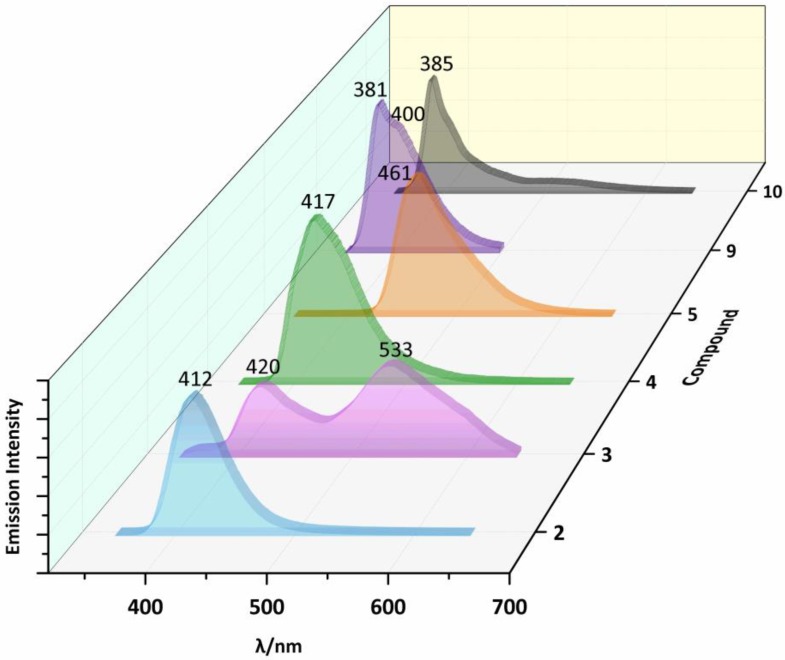
Solid state emission spectra (excitation at 300 nm) of compounds **2**–**5, 9**, and **10** at room temperature.

**Figure 3:**
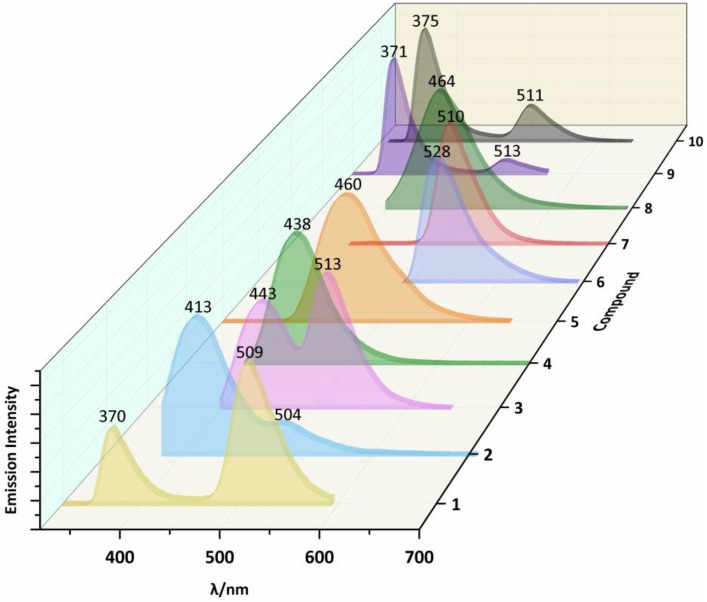
Emission spectra of compounds **1**–**10** (excitation at 270 nm for **1** and **9**, 350 nm for **2**, 380 nm for **3**, 360 nm for **4**, 300 nm for **5**, 470 and for **6**, 370 nm for **7**, 380 nm for **8**, and 310 nm for **10**) in *N,N*-Dimethylformamide (DMF) solution at room temperature.

The photoluminescent properties of compounds **1** (in solid) and **6**–**8** (in solid and DMF solution) have been determined and reported [41,42] and Figure 2 (in solid) and Figure 3 (in DMF solution) presents the emission spectra data of compounds **2**–**5**, **9**, and **10**, and Table 2 presents the emission bands. Compounds **1**–**10**, which bear the groups with varied electronegativity, show interesting photoluminescent properties in the solid state at room temperature. The maximal emission peaks of compounds **1**–**10** can be tuned from 381 nm to 533 nm when the substituent groups changed.

Only one band is detected in the emission spectra of compounds **1**, **2, 4**–**8**, and **10,** while two bands are detected in compounds **3** and **9**. In compound **1,** only a broad band at 409 nm is reported in the literature [41]. For compound **2**, only one band in its emission spectrum with high intensity at 412 nm is detected when excited at 300 nm. For **4**–**8** and **10,** their photoluminescent spectra also display one band, 417 nm for **4**, 461 nm for **5**, 425 nm for **6** [42], 415 nm for **7** [42], 483 nm for **8** [42], and 385 nm for **10**, when being excited at 300 nm. Compounds **3** and **9** show multiple photoluminescent behaviors in their spectra, a low intense one at 420 nm and another with higher intensity at 533 nm for **3** (excited at 300 nm), and two bands at 381 nm and 400 nm for **9** (excited at 300 nm). On account of the different substituents at the terpyridyl group, their emission peaks show different changes. When comparing with **1**, an obvious red shift phenomenon being observed in the compounds **2** (bearing *p*-methyl), **3** (*p*-methoxy), **4** (*p*-phenyl), **5** (*p*-tolyl), **8** (*o*-hydroxyl), **9** (*p*-carboxyl), and **10** (*p*-methylsulfonyl). The substituents at **2**, **3**, **6** (*p*-hydroxyl), **7** (*m*-hydroxyl), **9**, and **10** show peaks at low energy region being similar to those of **1**, located at a range of 504–528 nm. According to the already reported photoluminescent results of 4′-phenyl-terpyridine, the peak at ca. 380 nm is tentatively assigned to π-π*/MLCT [44]. The peaks of compounds **1**, **2**, **3**, **6**, **7**, **9**, and **10** at low energy region also show interesting patterns, which are temporarily assigned to n-π*/LMCT [45].

The photoluminescent properties of the compounds were also studied in solution. *N,N*-Dimethylformamide (DMF) was the selected solvent because of is solubility. The photoluminescent properties of compounds **1**–**10** in the DMF at room temperature are expressed by the emission spectra that are presented in Figure 3, and Table 2 lists the emission bands. All of them not only display interesting photoluminescent properties with strong emission, but also tune the maximal emission peaks from 370 nm to 528 nm due to the substituent groups. Compounds **4**–**8** only show one band at 438, 460, 528, 510, and 460 nm, while the other compounds display two peaks in the range of 370–443 nm and 504–513 nm. The results are different from those in the solid state, which indicate that the solvent DMF has a significant effect on the photoluminescence properties of the compounds.

### 2.4. Solution Stability

Stability in a buffer solution is a prerequisite for the study of compounds with great potential as anticancer drugs, which can be qualitatively confirmed by comparing the UV-visible absorption spectra that were measured at different times within 72 h at 37 °C [46]. Stock concentrations of the ten compounds were prepared in DMSO and freshly diluted with PBS for analysis. Figures S19–S28 show the results of the stability of the compounds and no obvious change in the UV-Vis spectra of the compounds is observed after 72 h incubated in PBS, which indicated that the compounds are almost stable in solution.

### 2.5. Antiproliferative Properties

**Figure 4:**
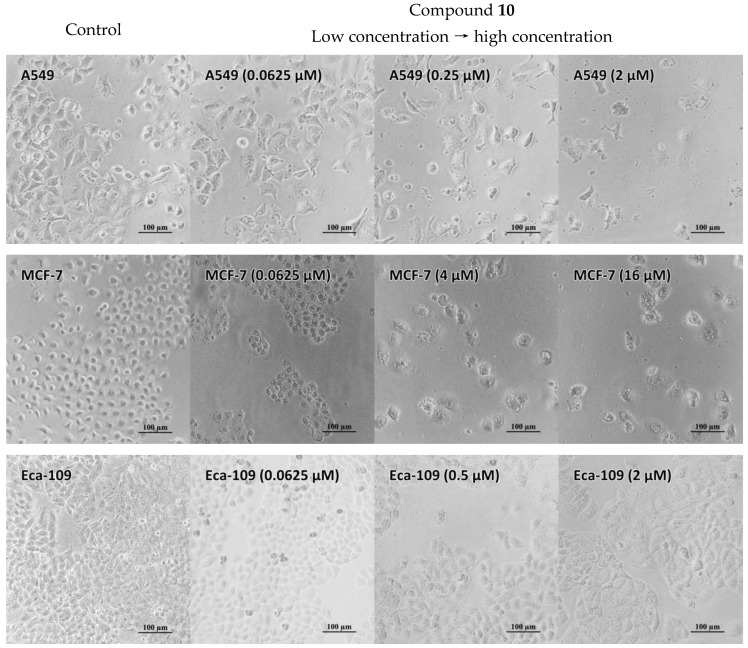
The microscopic photographs of the A-549, MCF-7, and Eca-109 cancer cells treated with increased concentrations of compound **10** at magnification of 200×.

Four human carcinoma cell lines, including A549 (lung adenocarcinoma), Bel-7402 (hepatocellular carcinoma), MCF-7 (breast adenocarcinoma), and Eca-109 (esophageal squamous carcinoma), were treated with various concentrations of compounds **1**–**10** (0.0624–4 µM) to evaluate the in vitro antiproliferative activities of the ten compounds. Figure 4 shows the live-cell images with or without compound **10** for 72 h. Figures S29–S31 provide the microscopic photographs for the other compounds. The microscopic analysis of the cancer cells that were treated with the compounds shows a decrease in the number of the cancer cells with the increasing compound concentrations. It is interesting to note that the different cells distinctively respond to these compounds. For A549 cells, the low concentrations of the compounds caused a swell of the cell body, blur of the cell boundaries, and fragment of some cell nucleus. A phenomenon was observed that the cells contract and the nuclei shrink, which results in a display of severe cell necrosis as the concentrations of the compounds increase. An obvious nucleus shrinkage was also observed in MCF-7 cells at low concentrations of the compounds, and many small black particles, which may be the lysate of cell organelles, appearing in the cytoplasm and surrounding culture medium at high concentrations of the compounds, and indicating that the cells have undergone severe necrosis. Regarding the Eca-109 line, cell swell is a noticeable phenomenon; especially, the body of the cells treated with compound **10** is almost twice that of the control cells. It is not surprising that nucleus shrinkage was also observed as the concentrations of the compounds increased. The cell morphological changes can be used as evidences for determining the mechanism of apoptosis, which is suggested to relate to the interaction of the nucleus and the compounds.

**Figure 5:**
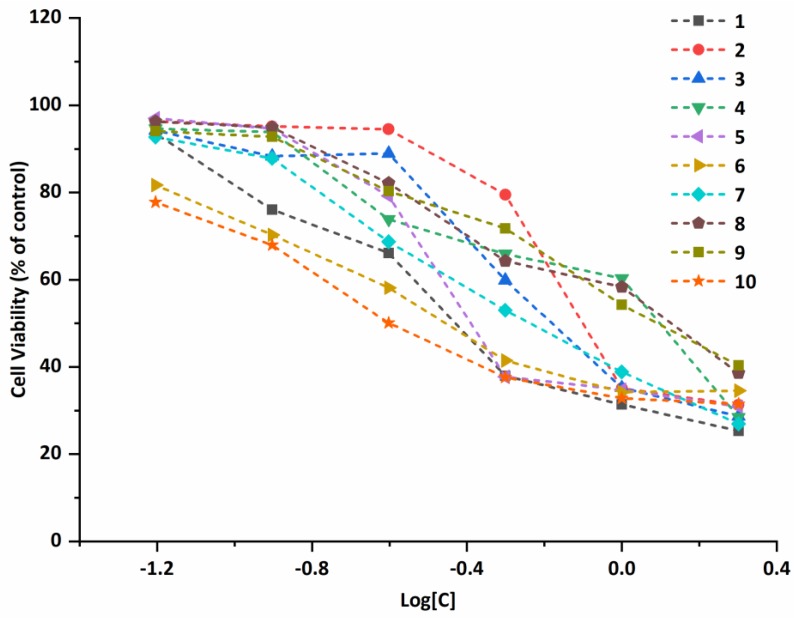
The plots of the cell viability vs. the concentration of compounds **1**–**10** against A549 cell line.

The plots of the cell viability vs. the concentration of compounds **1**–**10** against A549 cell line (Figure 5) show that all of the compounds exhibit a strong inhibitory effect against the selected cell lines. The viability of A549 cells decreased as the increasing the compound concentrations, exhibiting a dose-dependent manner. For Bel-7402, MCF-7, and Eca-109 cell lines, similar trends that the viability decreased as the increase of the compound concentrations are observed and are shown in the plots of the antiproliferative activities (Figures S32–S34).

**Figure 6:**
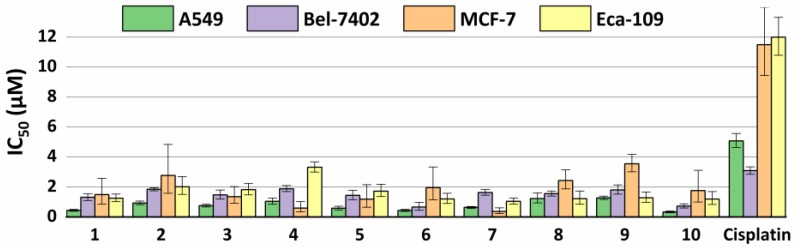
IC_50_ values (μM) from the dose-response assay of compounds **1**–**10** and the reference compound cisplatin in the A549, Bel-7402, MCF-7, and Eca-109 cell lines, after an incubation time of 72 h. The results shown are means ± SD of quadruplicate experiments.

The half maximal inhibitory concentration (IC_50_) for all of the compounds is calculated and listed in Table 3. Much lower IC_50_ values against the A549, Bel-7402, MCF-7, and Eca-109 cell lines are observed for compounds **1**–**10**, when comparing with that of cisplatin, which is a widely used anti-tumor drug [47]. From all of the compounds reported here, **10** presents the lowest IC_50_ value against the A549 cell line and compound **6** gives the lowest IC_50_ value against Bel-7402 cell line, while compound **7** is the best for MCF-7 and Eca-109 cell lines. These investigations show that compounds **1**, **5**–**7**, and **10** are more effective against A549 rather than the other cell lines, while compounds **4** and **7** are more effective against MCF-7 than the others. Additionally, compound **7** shows the highest efficiency against the line Eca-109. In summary, the A549 cell line is the most susceptible one to the tested compounds, and the MCF-7 cell line is the most tolerant one against all of the tested compounds (Figure 6). RAW 264.7, which is a kind of mouse monocyte macrophage, was used to evaluate the cytotoxicity of the synthesized compounds. The viability of RAW 264.7 cells that were treated with compounds **1**–**10** was tested following the same methods on tumor cell lines and Figure S35 show the plots of cell viability of compounds **1**–**10** in increasing concentration. The results show that the compounds promote the growth of RAW 264.7 cells at low concentration (<0.25 µM) and they inhibit the growth of the cells at high concentration (>0.5 µM).

### 2.6. Interaction of the Compounds with DNA.

Most anti-tumor drugs have functions in incorporating into the base pairs of DNA of tumor cells to interrupt their replication and transcription [48,49]. The decrease of cell viability, cell swelling, and cell necrosis, etc. observed in this study may be due to the effect of the compounds on DNA replication and transcription. The DNA interaction with the compounds was studied using multiple spectrophotometric methods, such as UV-vis absorption, fluorescence, and circular dichroism spectropolarimetry, in order to explore the mechanism of their anticancer activity.

#### 2.6.1. UV-vis Absorption Spectroscopy

Molecular absorption spectroscopy in the UV-vis region is an efficient tool for investigating the binding of compounds to DNA [50,51]. The UV-vis absorption spectra of compounds **1**–**10** are significantly perturbed by the addition of increasing amounts of DNA. Figure S36 shows the corresponding spectra for the ten compounds. Absorption bands at about 260 nm for CT-DNA (calf thymus DNA) were observed and the intensity increased following the increasing concentration of the DNA. The interaction between a compound and DNA can perturb the intraligand transitions. Absorption bands in the region 300–400 nm, which have been attributed to ILCT (intraligand charge transfer), were used to monitor the interaction of **1**–**10** with duplex DNA. The obvious hypochromism was clearly observed without any bathochromism when increasing quantities of CT-DNA were added to the compounds, which suggests an electronic interaction between the binding compounds and the biomolecule [52]. The absorption bands of compound **1** show a hypochromism of 45.6% upon the addition of DNA to [compound]/[DNA] = 0.12 without any significant shift in λ_max_. Similar features are observed for compounds **2**–**10**, since the comparable hypochromism without wavelength shifting occurs. Generally, a significant hypochromism with a red shift is known to be a characteristic of a strong π–π stacking interaction between the aromatic chromophore ligand of a metal complex and the aromatic rings of DNA bases, which is called intercalation interaction. The coupling of π orbitals, which are also partially filled by electrons, reduces the transition probability and, thus, results in hypochromism [53,54]. On the other hand, the hypochromism that is manifested in terms of a small decline in absorbance and either no or only minor changes in *λ*_max_ has been correlated to groove binding [55,56]. Accordingly, it can be suggested that compounds **1**–**10** interact with the DNA through the partial insertion of the aromatic rings of the ligands to the DNA duplex, or it is also likely that the compounds can bind to the DNA helix via a groove mode. It has been demonstrated that the structure of the compounds is one of the most important factors that contribute to the DNA binding affinity. However, some factors of the ligands, including size, geometry, hydrophobicity, and hydrogen-bonding ability, can also influence the overall affinity [57].

Binding constant (*K*_b_) is a useful parameter for evaluating the binding strength of a compound to the DNA and it can be determined from the variation in the electronic spectra before and after the addition of DNA by applying Benesi-Hildebrand equation [58,59] (Equation (1)), given below:

(1) $$\frac{A_{0}}{A-A_{0}}=\frac{\varepsilon_{f}}{\varepsilon_{b}-\varepsilon_{f}}+\frac{\varepsilon_{f}}{\varepsilon_{b}-\varepsilon_{f}}\frac{1}{K_{b}\left[DNA\right]}$$

where *A*_0_ is the initial absorbance of a free compound, *A* is the absorbance of a compound in the presence of DNA, ε_f_ corresponds to the extinction coefficient of a compound in its free form, and *ε*_b_ refers to the extinction coefficient of a compound in the bound form. The plot of *A*_0_/(*A* − *A*_0_) *versus* 1/[DNA] gives a straight line with an intercept of *ε*_f_/(*ε*_b_ − *ε*_f_) and a slope of *ε*_f_/*K*_b_(*ε*_b_ − *ε*_f_). The values of *K*_b_ are calculated from the ratio of the intercept to the slope and then summarized in Table 4. The binding constant *K*_b_ for **2** amounts to 4.78 × 10^4^ M^−1^, which suggests a strong affinity of the compound with CT-DNA. Comparable values of *K*_b_ (3.49 × 10^3^–9.79 × 10^4^) for compounds **4**–**10** are also observed. The *K*_b_ values of the compounds are similar to those of [Zn(dppt)_2_Cl_2_] (*K*_b_ = 1.97 × 10^5^ M^−1^) [60] and [Ni(dppt)_2_Cl_2_] (*K*_b_ = 1.0 × 10^4^ M^−1^) [61], being larger than those of many Cu(II) complexes with N-donor ligands (*K*_b_ = 2.2–10.9 × 10^3^ M^−1^ and *K*_b_ = 1.8–3.0 × 10^4^ M^−1^) [62,63]. The differences among these *K*_b_ values are most likely due to the additional functional group at the 4′-position of the terpyridyl unit, namely the substituent moiety. However, the binding constants of compounds **1**, **3,** and **10** are significantly lower than those of the others, thus indicating that the binding mode of these compounds is different. These low affinities may be due to the absence of hydrogen bonding interactions between the compounds and DNA.

#### 2.6.2. Fluorescence Titration

**Figure 7:**
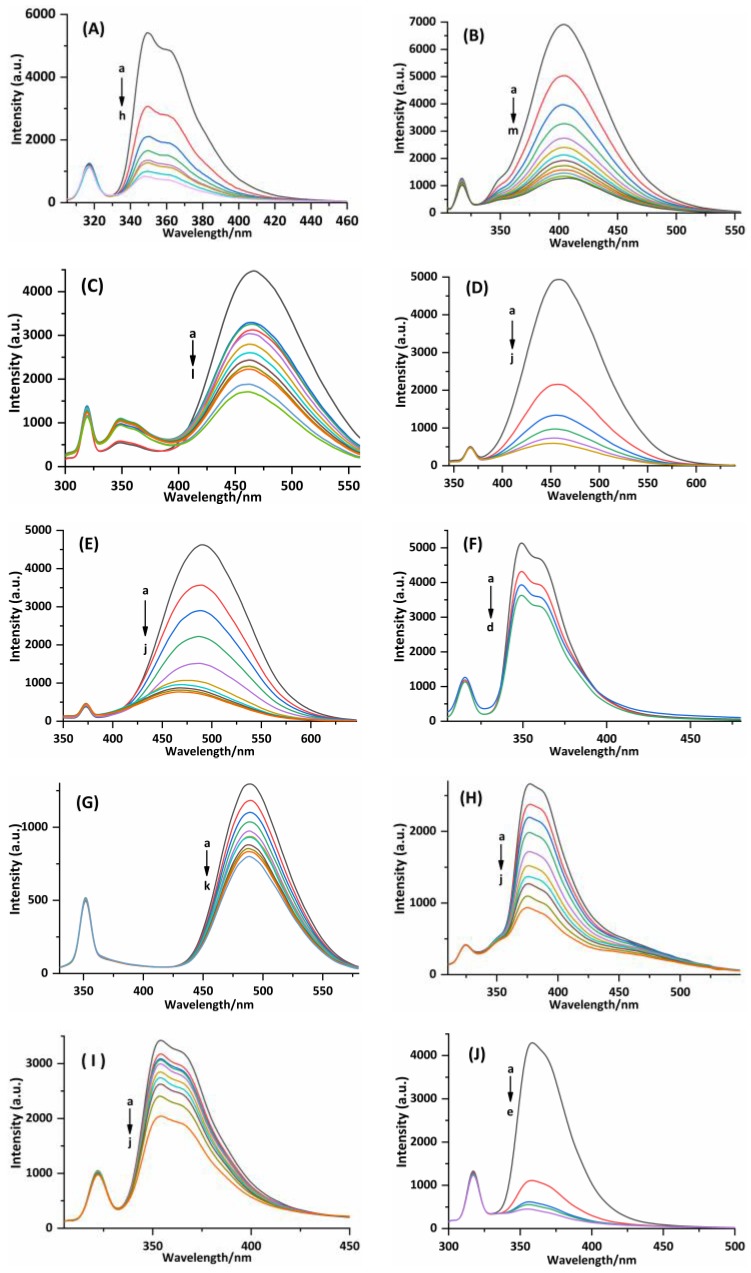
Fluorescence spectra of compounds **1**–**10** in the absence or presence of CT-DNA in Tris-HCl buffer (pH 7.2). (**A**) Emission spectra excited at 285 nm, c(**1**) =100 µM and c(CT-DNA) = 0, 10, 20, 30, 40, 50, 60, and 70 µM corresponding to the curves from a to h, respectively. (**B**) Emission spectra excited at 313 nm, c(**2**) = 0.25 µM and c(CT-DNA) = 0, 8, 16, 24, 32, 40, 48, 56, 64, 72, 80, 88, and 96 µM corresponding to the curves from a to m, respectively. (**C**) Emission spectra excited at 287 nm, c(**3**) = 1 µM and c(CT-DNA) = 0, 8, 16, 24, 32, 40, 48, 56, 64, 80, 96, and 112 µM corresponding to the curves from a to l, respectively. (**D**) Emission spectra excited at 325 nm, c(**4**) = 0.5 µM and c(CT-DNA) = 0, 4, 8, 12, 16, and 20 µM corresponding to the curves from a to f, respectively. (**E**) Emission spectra excited at 330 nm, c(**5**) = 0.5 µM and c(CT-DNA) = 0, 2, 3, 4, 6, 8, 10, 12, 14, and 16 µM corresponding to the curves from a to j, respectively. (**F**) Emission spectra excited at 284 nm, c(**6**) = 10 µM and c(CT-DNA) = 0, 8, 16, and 24 µM corresponding to the curves from a to d, respectively. (**G**) Emission spectra excited at 313 nm, c(**7**) = 10 µM and c(CT-DNA) = 0, 8, 16, 24, 32, 40, 48, 56, 64, 80, and 96 µM corresponding to the curves from a to k, respectively. (**H**) Emission spectra excited at 289 nm, c(**8**) = 30 µM and c(CT-DNA) = 0, 1.8, 3.6, 5.4, 9, 12.6, 16.2, 19.8, 27, and 32 µM corresponding to the curves from a to j, respectively. (**I**) Emission spectra excited at 289 nm, c(**9**) = 0.5 µM and c(CT-DNA) = 0, 1.8, 3.6, 7.7, 12.6, 16.2, 22.4, 33, and 49 µM corresponding to the curves from a to j, respectively. (**J**) Emission spectra excited at 285 nm, c(**10**) = 0.5 µM and c(CT-DNA) = 0, 8, 16, 24, and 32 µM corresponding to the curves from a to e, respectively.

Fluorescent technique is widely used to obtain significant information regarding the interaction between a compound and a biomacromolecule, such as quenching constant, binding constant, and sites [57,64]. Accordingly, the technique was used to study the interaction between the compounds and DNA. All of the compounds show a high intensity fluorescent emission peak, while pure CT-DNA exhibits no fluorescent signal. The fixed amount of the compounds was titrated with the increasing amounts of CT-DNA. Figure 7 illustrates the results of emission titration for the compounds with DNA at 298K. The fluorescence of the compounds is remarkably quenched by CT-DNA. The reduction in fluorescent intensity indicates that interaction between CT-DNA and the compounds occurs. Whereas, there are no changes in both the position and the shape of the fluorescent spectra of the compounds, which suggests that, rather, the non-covalent bonds than covalent ones form between the compounds and CT-DNA [65].

#### 2.6.3. Circular Dichroism (CD) Spectroscopic Studies

CD measurement is very sensitive to detecting minor conformational variations of DNA that are induced by small molecule binding [66,67]. The heterocyclic bases of DNA are themselves achiral, but they become chiral when placed within the framework of the chiral sugar–phosphate backbone. Stereospecific coupling of degenerate or near-degenerate electronic transitions, exciton coupling, of neighboring base chromophores results in a CD signal [68]. The observed CD spectrum of free DNA shows a positive band at 277 nm due to base stacking and a negative band at 246 nm from the right-handed helicity. The two bands are the known features of a right-handed B-form DNA [69]. The characteristic bands of DNA are quite sensitive to the mode of DNA interactions with small molecules. Simple groove binding and electrostatic interaction do not cause any appreciable changes in the base-stacking and helicity bands, while intercalation influences the intensities of the two bands [70].

**Figure 8:**
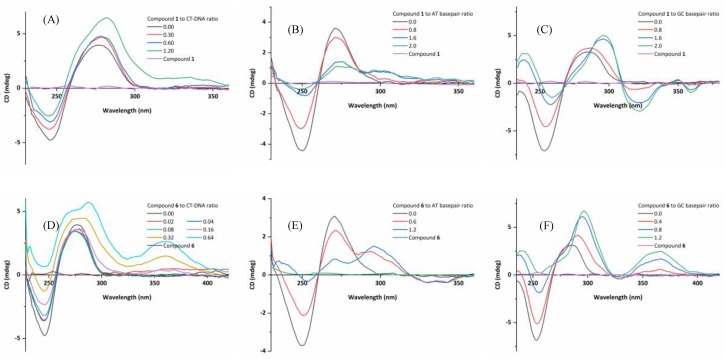
Circular dichroism spectra of three kinds of DNA in the presence or absence of compounds **1** and **6** in Tris-HCl buffer (pH 7.2), at 20 °C. (**A**) CT-DNA (4.0 × 10^−4^ M) treated with compound **1**, (**B**) ds(AT)_6_ (1.58 × 10^−5^ M) treated with compound **1**, (**C**) ds(GC)_6_ (1.58 × 10^−5^ M) treated with compound **1**, (**D**) CT-DNA (4.0 × 10^−4^ M) treated with compound **6**, (**E**) ds(AT)_6_ (1.44 × 10^−5^ M) treated with compound **6**, and (**F**) ds(GC)_6_ (1.58 × 10^−5^ M) treated with compound **6**.

The CD spectra were measured in the presence of compounds **1**–**10** with various ratios of the compounds to CT-DNA, as shown in Figure 8 and Figures S36 and S37. It is found that the CD spectra of CT-DNA displayed obvious changes in both positive and negative bands when the compounds were incubated with DNA, while the free compounds showed no CD signal in the measuring wavelength range. All of the CD spectral bands of the DNA alone and with the compounds are tabulated in Table S1. The intensity of the negative bands decreases (shifting to zero level) and the ellipticity of the positive bands increases with shifts of the *λ*_max_. The results suggest that the binding of the compounds to CT-DNA reduces the right handed helicity of CT-DNA and increases the base stacking degree of CT-DNA [71]. The increased CD signal around 275 nm with an increased concentration of the compounds is important for stating their intercalation binding mode [72]. Based on this, the recorded similar CD changes demonstrate an intercalating interaction mode between the ten compounds and CT-DNA.

The next investigation is to find out the DNA binding mode with G–C rich or A–T rich sequences. DNA molecules adopt more or less different structures depending on the base sequence, and thus the CD spectra differ from each other as well. The self-complementary duplexes containing alternating purine–pyrimidine sequence of d(GC)n and d(AT)n dinucleotides display very similar spectra to the native sequentially heterogeneous DNA, and their conformations are typical B form. The CD spectra of two synthetic duplex oligonucleotides, ds(AT)_6_ and ds(GC)_6_ with compounds **1**–**10** were measured and are depicted in Figure 8 and Figures S37 and S38. Table S1 tabulates the CD spectral bands of ds(AT)_6_, ds(GC)_6_ with the ten compounds. As shown in the figures, ds(AT)_6_ provides a positive band around 271 nm and a negative band around 250 nm, while the positive band of ds(GC)_6_ noticeably shifts to 286 nm and the negative one to 253 nm. These four elliptical components are highly sensitive to the interaction of small molecules with DNA. Thus, spectral variations in CD bands in terms of band position and intensity were observed upon the addition of the compounds in the increasing concentration (keeping DNA concentration constant). When compound **1** interacts with ds(AT)_6_, the band attributed to base stacking (at 271 nm) shows a 70% decrease in positive ellipticity with no considerable shift in its position, at the same time, another positive band (at 295 nm) appears and increases with the concentration of the compound. Correspondingly, the band attributed to helicity of B-DNA at 250 nm undergoes a slight red shift (hypochromism) of 1 nm (to 251 nm), along with 92% reduction in the negative ellipticity at the highest compound/DNA molar ratios. For compounds **2**–**10**, at the same mole ratio, the hypochromism of both the positive and negative bands are obviously different, owing to the variation of the 4′-substituent on the terpyridyl. It can be seen that the structure and the functional substituents significantly affect the intercalating interaction mode between the compounds and DNA. These spectral alterations referring to above-mentioned characteristic dichroic components indicate that the perturbation in the secondary structure of B-DNA mainly disrupts nucleobases stacking interactions and helix local base-pair geometry. The elliptical intensity of 271 nm band is directly related to winding angle (or propeller twist) and a substantial reduction in its magnitude after a compound intercalating with DNA lead to increase in the winding angle and decrease in the propeller twist. Further, the loss of intensity can be associated with the conformational transition in DNA duplex with the change in the number of base-pairs in each turn of helix, for instance, from B-form DNA (10.4 bp per turn) to C-form DNA (9.4 bp per turn).

Subsequently, a more than 90% reduction in ellipticity along with red shift at 250 nm band position reflecting a distortion in the duplex right-handed helical geometry can ascertain this conformational change from B- to C-form. In the meantime, the rising new positive band at 295 nm announces the formation of C-form DNA and the transformation from B- to C-form. For ds(GC)_6_, the decrease of the negative band and striking increase of the positive band along with a red shift from 286 nm to 295 nm also indicate the conformational change from B- to C-form. Interestingly, the abnormal change of the positive band ellipticity might be due to the compounds with the three coplanar rings participating and facilitating the π…π stacking of base pairs, which will be discussed in the molecular docking studies section.

The change mode of the positive band is also found in compounds **2**, **5,** and **6** interacting with CT-DNA. A global interpretation of all of the data suggests that the titled compounds induce DNA to form a new conformation with both B- and C-form characteristic features.

#### 2.6.4. Induced CD (ICD) Characteristics

Many DNA-binding compounds are achiral and, as such, are optically inactive. However, a compound can acquire an ICD signal through the coupling of its electric transition moments and the DNA bases upon interaction with DNA. The observation of an ICD signal within the absorption bands of the achiral compound is immediately indicative of a compound–DNA interaction [67]. As further evidence for the binding mode of a compound and DNA, ICD bands in the region of 300–400 nm were obtained and are shown in Figure 8 and Figures S37 and S38.

**Scheme 2:**
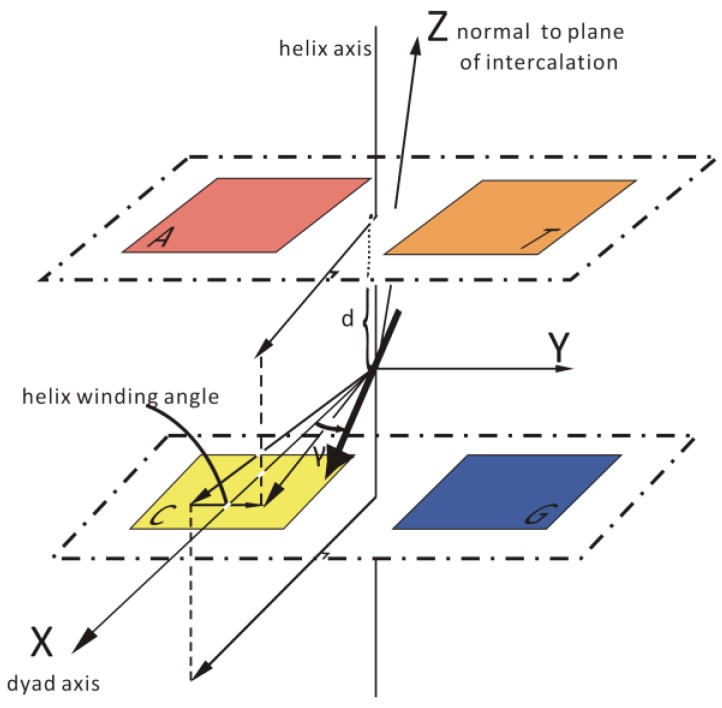
Schematic view of the duplex with an intercalated transition moment.

The exact position and orientation of a compound in DNA and the environment around its binding site greatly affect the ICD of a bound compound. While considering the geometry of a compound with DNA, it is usual to use the reference points and principal axes of the DNA binding site and the compound, such as the dyad and helix axes of DNA and the transition moments and molecular axes of the compound (see Scheme 2). In general, the ICD signal is dependent on the angular orientation of the intercalator relative to the base-pair dyad axis for an intercalation mode close to the center of the system. The orientation of the transition dipole within the intercalation plane is defined by the angle γ, as shown in Scheme 2. The ICD signal is typically positive if the transition moment of a compound is oriented parallel to the dyad axis (γ = 0°) and negative if perpendicular (γ = 90°) for an intercalator with a transition moment oriented along its long axis [73,74].

These observations are made for transition moments oriented along the long axis of a compound and appropriate considerations must be made for the transition moments of a compound oriented in other directions. For a compound that is not located in the center of the intercalation site, the sign and magnitude of the signal are both affected in a more complicated manner by the lateral displacement of the compound as well as the nature of the base pairs surrounding it. A compound bound in the minor groove of B-DNA, with a transition moment that is oriented along the groove (45° to the bases), will exhibit a strong, positive signal. The intensity and +/− sign of the ICD signal for a compound bound in the major groove are more variable due to the high numbers of possible orientations permitted by the width of the major groove.

As shown in Figure 8 and Figures S37 and S38, the spectra of compounds **1** and **4** are complicated, while those of **2**, **3**, and **6** are basic. The ds(AT)_6_ and ds(GC)_6_ both exhibit weak negative bands in the presence of compounds **2** or **3**, perhaps due to that the intercalators incline to parallel to the pseudo-dyad and the γ is at near 45°. The spectra of compound **6** with CT-DNA, ds(AT)_6_, and ds(GC)_6_ exhibit a relatively strong positive ICD band. The positive ICD spectrum in this region is a characteristic of the intercalated chromophore with the transition moment of intercalator oriented parallel to the dyad axis of the DNA base pairs (γ at near 0°). The spectrum of compound **4** with ds(GC)_6_ shows a biphasic ICD signal, namely a negative band at low wavelengths and a positive band at high wavelengths, probably arising from the intercalation of compound **4** with DNA through two different directions. Meanwhile, compound **1** exhibits a more complicated biphasic signal than compound **4** when it interacts with ds(GC)_6_, where it gives two negative bands and a small positive band. It seems that it is a combination of a wide positive band and a narrow negative band, both of which are assigned to tge two binding conformations caused by the rotation of **1** around the helix axis, one being γ at close to 0° and the other at near 90°. These interesting signals will be discussed further in the molecular docking studies.

### 2.7. Molecular Docking Studies

Computational docking is a useful tool for gaining an understanding of synthesized compounds and biological target interactions, which is very important in drug discovery. In this study, the structures of the synthesized zinc(II) complexes were used for molecular docking methods while using AutoDock Tools (ADT) version 1.5.6 and AutoDock version 4.2.5.1 docking programs [75,76], which are interactive molecular graphics programs, for examining the compound–DNA interactions to investigate the potential binding mode and energy.

The docked ligand conformation was analyzed in terms of energy, hydrogen bonding, and hydrophobic interaction between the synthesized zinc complexes and B-DNA (PDB ID: 1BNA). Detailed calculations of the compound–DNA interactions were carried out, and the final results of the compounds and receptor were analyzed. From the docking results, the free energy of the binding of the compounds with DNA was calculated and Table 5 shows the details.

**Figure 9:**
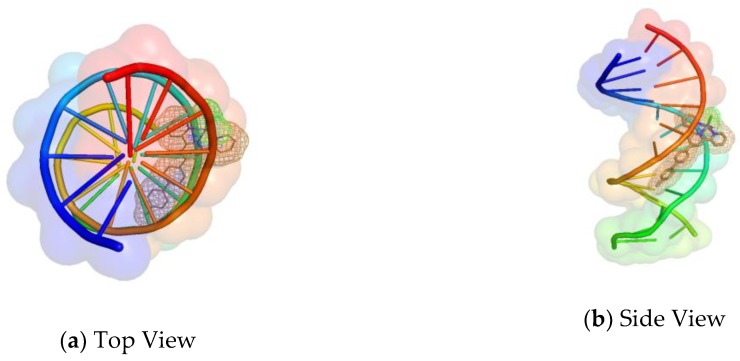
(**a**) Top View and (**b**) Side view of the most favorable orientation of compound **5** bound with the minor groove of the B-DNA (PDB ID: 1BNA).

The in silico molecular docking experiment reveals that the docked compounds fit into the DNA comfortably, involving van der Waals interaction, hydrophobic and hydrogen bonding contacts with DNA functional groups, and resulting in the binding energy between −8.70 and −11.83 kcal mol^−1^. Figure 9 and Figures S39–S45 show the binding interactions of all the zinc(II) complexes with the B-DNA receptor.

No hydrogen bond is detected in the most favorable pose of compounds **1**, **2**, **4**, and **5**, while compounds **3** and **6**–**10** show the binding energy value from −8.70 to −10.83 kcal mol^−1^ with one hydrogen bond. For compounds **3**, **9,** and **10**, one N–H at the guanine interacts with the oxygen at the 4′-substitute methoxyl, carboxyl, or methylsulfonyl, and it forms a hydrogen bond with the bond lengths of 1.877, 1.837, and 1.765 Å, respectively. The *m*-hydroxyl of compound **8** interacts with the oxygen at a thymine to form a hydrogen bond. However, for **6** and **7**, the O–H forms a hydrogen bond with an oxygen of the phosphate group at the backbone of the DNA. Table 6 summarizes the details of the hydrogen bonds. Interestingly, the binding energy values of compounds **4** and **5**, from −11.43 to −11.83 kcal mol^−1^, are much lower than those of the compounds that form hydrogen bonds. This anomalous phenomenon is due to the fact that both the volumes and shapes of compounds **4** and **5** with multiple rotatable aryl rings that fit in the helical conformation of the minor groove.

A molecular modelling study was undertaken to explore further the nature of the DNA binding selectivity to the zinc complexes. The three kinds of duplexes, (1) ds(ATGCAT)_2_, (4JD8) (2) AT-rich duplexes ds(ATAT)_2_ (2DA8M), and (3) GC-rich duplexes ds(GCGCGC)(CGCGCG) (2ROUM) were chosen for the docking simulation with the ten compounds. The binding energy of the ten compounds was calculated and are listed in Table 5, which is in the range of −7.38–−8.94 kcal mol^−1^. The results show that the intercalation of the compounds to DNA is spontaneous and the van der Waals interaction and π–π interaction play major roles in the interaction between the molecules and DNA functional groups. For most of the compounds (except **3** and **8**), the decreasing order of the binding energy for the duplexes is 4JD8 > 2DA8M > 2ROUM. An analysis of the interactions and the results of the binding energy suggests the order of nucleotide sequence binding selectivity being ATGC > ATAT > GCGC.

**Figure 10:**
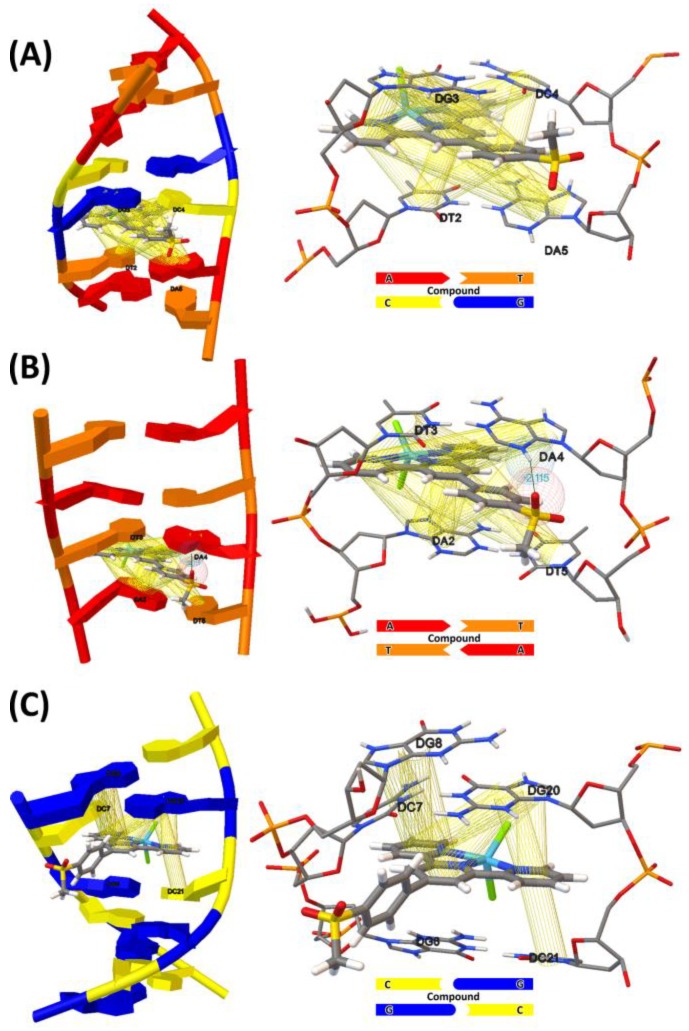
View of the energy minimized docked poses of compound **10** with: (**A**) ds(ATGCAT)_2_ (PDB ID: 4JD8), (**B**) ds(ATAT)_2_ (PDB ID: 2DA8M), and (**C**) ds(CGCGCG)_2_ (PDB ID: 2ROUM).

The graphical program AutoDock Tools 1.5.6 was used to analyze the relative orientation and the interaction that were determined from the results of the docking calculation. The views in Figure 10 and Figures S46–S54 show the different modes by which the compounds intercalate in the different DNA sequences. The different number of observed stacking π…π, H-bonding, C–H…π, and C=O…π interactions suggests that they are related to the strength of the ligand binding mode in accord with the obtained binding energies. Thus, the docking results are reliable and warrant further analysis and discussion of the nature of the sequence recognition. The preliminary assessment shows that the orientations of the substituent moiety relevant to the planar middle pyridyl ring are important. The intercalation of the terpyridyl in a given site and mode anchors it to the DNA and directs the orientation of the 4′-substituent for the interaction with the nucleobases by H-bonding and π…π stacking. It is found that many hydrogen bonds are formed between the compounds and the base pairs (Table 6). Compound **6** forms two hydrogen bonds with 4JD8 and 2DA8M, while compound **7** forms hydrogen bonds with all three kinds of duplexes benefiting from the presence of the hydroxyl groups. Compound **8** is also involved in the hydrogen bond interaction with 2DA8M and 2ROUM, and compound **10** only forms a hydrogen bond with 2DA8M, while other compounds show no hydrogen bond formation.

**Figure 11:**
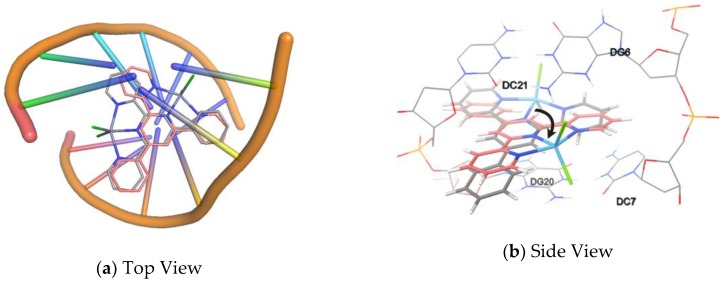
(**a**) Top view and (**b**) side view of two selected docked poses of compound **6** with ds(CGCGCG)_2_ (PDB ID: 2ROUM).

Through the results of molecular simulation it is observed that the π…π stacking between the terpyridyl moiety and G–C base pairs is much stronger, involving more bases in far position than that with A–T base pairs. These powerful π…π stackings promote more G–C base pairs that are closely aligned, with the decrease of the *d* value and the increase of the number of base pairs in each turn of helix, resulting in the rise of CD positive band. On the other hand, the hydrogen bond between *p*-hydroxyl and the oxygen atom of the sugar moiety fixes the orientation of compound **6** (γ close to 0°) when intercalating, which generates the strong positive ICD bands. For compound **1**, Figure 11 presents two docking poses: one of them can be rotated for ca. 120° around the helix axis to give another one. Compound **1** intercalates with ds(GC)_6_ to give conformations 1 and 2. When it shows the conformation described as 1, the transition moment is oriented closing to the dyad axis, which induces a large negative band. Meanwhile, it can also intercalate with DNA with conformation 2 leading to a positive band at the same position in CD spectra. The determined CD spectra are the superposition of the two bands that are presented in Figure 8. Similarly, the biphasic ICD signal of CT-DNA–compound **4** is a result of the mixture of the two dominant conformations that one of them can be turned into another by a rotation (*ca.* 90°) around the helix of DNA and a lateral displacement in the base pairs. For compounds **2** and **3**, the γ is at near 45° when the molecules at the energy minimized the docked poses. As a result, weak negative bands appear in both the ds(AT)_6_ and ds(GC)_6_ spectra. The transition moment of compound **6** at the most favorable pose tends to oriented parallel to the dyad axis of the DNA base pairs and the γ becomes smaller due to lack of hydrogen bonding; meanwhile, strong positive ICD bands are observed in the spectra of compound **6** with CT-DNA, ds(AT)_6_, and ds(GC)_6_. There is no obvious ICD band observed for compounds **5**, **7**, **8**, **9**, and **10**, which might be attributable to the coexistence of multiple docking conformations and the lack of dominant one.

Taking all of the results obtained above together, compounds **1**, **3,** and **5,** which show that obvious changes in CD spectra present better in vitro antiproliferative activities than compounds **2**, **4**, **8**, and **9** do. For compounds **6**, **7**, and **10**, significant reductions in ellipticity, as well as the smaller IC_50_ values than those of the others, are observed. This phenomenon may be related to the ability of the compounds to form hydrogen bonds with DNA.

## 3. Materials and Methods

### 3.1. Chemicals and Reagents

All of the chemicals and solvents were analytically pure and used without further purification, unless specially noted. Ultrapure water (18.2 MΩ·cm) was used in all of the experiments. CT-DNA was purchased from Solarbio Science & Technology Co., Ltd. (Beijing, China). The purity of the CT-DNA was checked by monitoring the absorption ratio at 260/280 nm (A260/A280), and the ratio was observed 1.82, which indicated that DNA was fully free of protein [77]. The concentration of CT-DNA per nucleotide phosphate was calculated from the absorbance at 260 nm by using ε = 6600 M^−1^cm^−1^. Sangon Biotech synthesized the single strand (AT)_6_ and (GC)_6_ (Shanghai, China). The double strand ds(AT)_6_ and ds(CG)_6_ were annealed from two complementary 12-mersoligonucleotide (AT)_6_ and (GC)_6_. The stock solutions of DNA were prepared by dissolving in the 5 mM Tris-HCl, 50 mM NaCl buffer (pH 7.2) at 4 °C, and the resultant homogeneous solutions were used.

### 3.2. Physical Measurements

The IR spectra were obtained with a Nicolet iS10 spectrophotometer (Thermo Scientific). ^1^H NMR spectra were measured on a Bruker AVANCE III HD 600 MHz spectrometer. The ESI-MS spectra were measured on an Exactive mass spectrometer (Thermo Scientific). Elemental analyses (C, H, N) were performed on a Perkin-Elmer 2400 series II analyzer. DNA concentration was measured by a NanoDrop 8000 spectrophotometer (Thermo Scientific).

### 3.3. Synthesis of Ligands and the Complexes

For complexes **2**, **3**, **5**, and **10**, the dichloromethane solutions of the corresponding ligands were added dropwise to methanol solutions of ZnCl_2_ and the system was stirred for 24 h. Filtration led to the separation of the powder of compound from the mother solution, which was dried in a desiccator. For complex **4**, the dimethyl formamide solution of **L^4^** was added dropwise to the dimethyl formamide solution of ZnCl_2_ and the system was stirred for 24 h. A large amount of diethyl ether was added to the mother solution to obtain the crystals and separate by filtration, and then dry in a desiccator. For complex **9**, a mixture of ZnCl_2_, **L^9^** and distilled water was sealed in a stainless reactor with a teflon liner and heated at 120 °C for two days to obtain the crystals of **9**. Subsequently, IR, ^1^H NMR, elemental analysis, and single crystal X-ray diffraction characterized the structures of compounds **2**–**5, 9,** and **10**.

[ZnCl_2_**L^2^**] (**2**) Yield: 0.36 g, 84%. Anal. calcd for C_22_H_17_Cl_2_N_3_Zn·H_2_O: C, 55.31; H, 4.01; N, 8.80%. Found: C 55.09, H 3.83, N 8.78%. ^1^H NMR (600 MHz, DMSO-*d*_6_) δ 9.04 (s, 2H), 8.92 (d, *J* = 7.5 Hz, 2H), 8.83 (s, 2H), 8.31 (t, *J* = 7.4 Hz, 2H), 8.18 (d, *J* = 7.4 Hz, 2H), 7.87 (s, 2H), 7.45 (d, *J* = 7.7 Hz, 2H), and 2.44 (s, 3H). ESI-MS: [ZnL^2^–H^+^]^+^: (386.06, 54%), [ZnClL^2^]^+^: (422.04, 100%), [Zn_2_Cl_3_L^2^_2_]^+^: (883.04, 6%). IR (KBr disc, cm^−1^, s = strong, m = medium, w = weak): 3058 (m, ν_C–H_), 3031 (w, ν_C–H_), 2922 (m, ν_C–H_), 2853 (w, ν_C–H_), 1604 (s, ν_C=C_), 1573 (m, ν_C=C_), 1550 (m, ν_C=C_), 1478 (s, ν_C=C_), 1425 (m, ν_C=C_), 1408 (m, ν_C=C_), 1249 (m, β_C–H_), 1016 (m, β_C–H_), 897 (m, γ_C–H_), 823 (m, γ_C–H_), 795 (s, γ_C–H_), 732 (m, γ_C–H_), and 497 (m). Slow evaporation of its DMF solution led to colorless crystals that were suitable for X-ray analysis.

[ZnCl_2_**L^3^**] (**3**) Yield: 0.30 g, 71%. Anal. calcd for C_22_H_17_Cl_2_N_3_OZn·H_2_O: C, 53.52; H, 3.88; N, 8.51%. Found: C 53.03, H 3.81, N 8.39%. ^1^H NMR (600 MHz, DMSO-*d*_6_) δ 8.97 (s, 2H), 8.88 (d, *J* = 8.0 Hz, 2H), 8.82 (d, *J* = 5.0 Hz, 2H), 8.29 (t, *J* = 7.8 Hz, 2H), 8.25 (d, *J* = 8.4 Hz, 2H), 7.85 (t, *J* = 6.2 Hz, 2H), 7.16 (d, *J* = 8.3 Hz, 2H), and 3.88 (s, 3H). ESI-MS: [ZnClL^3^]^+^: (438.03, 100%), [Zn_2_Cl_3_L^3^_2_]^+^: (915.03, 15%). IR (KBr, cm^−1^): 3078 (m, ν_C–H_), 3056 (m, ν_C–H_),2841 (m, ν_C–H_), 1595(s, ν_C=C_), 1573(s, ν_C=C_), 1547(s, ν_C=C_), 1523 (s, ν_C=C_), 1473 (s, ν_C=C_), 1434 (s, δ_C–H_), 1408 (s, ν_C=C_), 1311 (m, β_C–H_), 1285 (m, β_C–H_), 1241 (s, β_C–H_), 1183 (s, β_C–H_), 1014 (s, β_C–H_), 843 (s, γ_C–H_), 787 (s, γ_C–H_), 746 (m, γ_C–H_), 727 (m, γ_C–H_), and 658 (m), 637 (m), 577 (s). Slow evaporation of its DMF solution led to colorless crystals that were suitable for X-ray analysis.

[ZnCl_2_**L^4^**] (**4**) Yield: 0.21 g, 78%. Anal. calcd for C_27_H_19_Cl_2_N_3_Zn·0.5H_2_O: C, 61.10; H, 3.80; N, 7.92%. Found: C 60.99, H 3.76, N 8.00%. ^1^H NMR (600 MHz, DMSO-*d*_6_) δ 9.19 (s, 2H), 9.02 (d, *J* = 7.6 Hz, 2H), 8.87 (d, *J* = 3.2 Hz, 2H), 8.42 (d, *J* = 7.4 Hz, 2H), 8.38 (t, *J* = 7.8 Hz, 2H), 8.00 (d, *J* = 7.9 Hz, 2H), 7.91 (d, *J* = 6.3 Hz, 2H), 7.86 (d, *J* = 7.5 Hz, 2H), 7.56 (t, *J* = 7.6 Hz, 2H), and 7.47 (t, *J* = 7.8 Hz, 1H). ESI-MS: [L^4^+H^+^]^+^: (386.16, 50%), [ZnL^4^–H^+^]^+^: (451.95, 42%), [ZnClL^4^]^+^: (487.93, 100%). IR (KBr, cm^–1^): 3059 (w, ν_C–H_), 3029 (w, ν_C–H_), 1668 (w, δ_C–H_), 1612(s, ν_C=C_), 1598 (s, ν_C=C_), 1573 (m, ν_C=C_), 1548 (m, ν_C=C_), 1500 (w, ν_C=C_), 1475 (s, ν_C=C_), 1428 (s, ν_C=C_), 1391 (m), 1301 (m, β_C–H_), 1252 (m, β_C–H_), 1161(w, β_C–H_), 1081(m, β_C–H_), 1066 (m, β_C–H_), 1032(m, β_C–H_), 1016 (s, β_C–H_), 1008(s, β_C–H_), 895(m, γ_C–H_), 830 (s, γ_C–H_), 793 (s, γ_C–H_), 771 (s, γ_C–H_), 731 (s, γ_C–H_), 659 (s), and 640 (s). Slow evaporation of its DMF solution led to colorless crystals that were suitable for X-ray analysis.

[ZnCl_2_**L^5^**] (**5**) Yield: 0.20 g, 74%. Anal. calcd for C_28_H_21_Cl_2_N_3_Zn·CH_2_Cl_2_: C, 56.11; H, 3.73; N, 6.73%. Found: C 55.85, H 3.87, N 6.73%. ^1^H NMR (600 MHz, DMSO-*d*_6_) δ 9.12 (s, 2H), 8.96 (d, *J* = 7.8 Hz, 2H), 8.85 (d, *J* = 3.6 Hz, 2H), 8.36 (d, *J* = 7.9 Hz, 2H), 8.32 (t, *J* = 7.7 Hz, 2H), 7.93 (d, *J* = 8.1 Hz, 2H), 7.87 (t, *J* = 5.4 Hz, 2H), 7.74 (d, *J* = 7.9 Hz, 2H), 7.35 (d, *J* = 7.9 Hz, 2H), and 2.39 (s, 3H). ESI-MS: [L^5^+H^+^]^+^: (400.18, 36%), [ZnL^5^–H^+^]^+^: (462.09, 73%), [ZnClL^5^]^+^: (498.07, 100%). IR (KBr, cm^−1^): 3057 (w, ν_C–H_), 3028 (w, ν_C–H_), 2919 (w, ν_C–H_), 2840 (w, ν_C–H_), 1598 (s, ν_C=C_), 1573 (m, ν_C=C_), 1545 (m, ν_C=C_), 1505 (w), 1475 (s, ν_C=C_), 1430 (m, ν_C=C_), 1401 (m, ν_C=C_), 1302 (w, β_C–H_), 1249 (m, β_C–H_), 1161 (m, β_C–H_), 1014 (s, β_C–H_), 893 (m, γ_C–H_), 815 (m, γ_C–H_), 787 (s, γ_C–H_), 731 (m, γ_C–H_), 684 (m), 663 (m), and 638 (m). Slow evaporation of its DMF solution led to colorless crystals that were suitable for X-ray analysis.

[ZnCl_2_**L^9^**] (**9**) Yield: Anal. calcd for C_22_H_15_Cl_2_N_3_OZn·1.5H_2_O: C, 51.14; H, 3.51; N, 8.13%. Found: C 51.41, H 3.30, N 8.11%. ^1^H NMR (600 MHz, DMSO-*d*_6_) δ 13.31 (s, 1H), 9.18 (s, 2H), 8.99 (d, *J* = 7.6 Hz, 2H), 8.87 (d, *J* = 3.9 Hz, 2H), 8.40 (d, *J* = 8.3 Hz, 2H), 8.37 (d, *J* = 7.2 Hz, 2H), 8.20 (d, *J* = 8.0 Hz, 2H), and 7.91 (t, *J* = 5.4 Hz, 2H). ESI–MS: [L^9^+H^+^]^+^: (355.10, 100%), [ZnL^9^–H^+^]^+^: (421.89, 41%), [ZnCl_2_L^9^–OH^–^]^+^: (472.21, 1%). IR (KBr, cm^–1^): 3510 (w, ν_OH_), 3059 (w, ν_C–H_), 1712 (s, ν_C=O_), 1601 (s, ν_C=C_), 1575 (m, ν_C=C_), 1548 (m, ν_C=C_), 1477 (s, ν_C=C_), 1427 (s, ν_C=C_), 1400 (m, ν_C=C_), 1306 (m, ν_C–O_), 1243 (s, β_C–H_), 1120 (m, β_C–H_), 1111 (m, β_C–H_), 1017 (s, β_C–H_), 898 (m, γ_C–H_), 867 (m, γ_C–H_), 798 (s, γ_C–H_), 776 (s, γ_C–H_), 734 (m, γ_C–H_), 699 (m, γ_C–H_), 661 (m), and 642 (m). Slow evaporation of its DMF solution led to colorless crystals which were suitable for X-ray analysis.

[ZnCl_2_**L^10^**] (**10**) Yield: 0.53 g, 78%. Anal. calcd for C_22_H_17_Cl_2_N_3_O_2_SZn·1.5H_2_O: C, 47.97; H, 3.66; N, 7.63%. Found: C 48.14, H 3.45, N 7.58%. ^1^H NMR (600 MHz, DMSO-*d*_6_) δ 9.18 (s, 2H), 8.96 (d, *J* = 8.0 Hz, 2H), 8.86 (s, 2H), 8.51 (d, *J* = 8.0 Hz, 2H), 8.37 (t, *J* = 7.7 Hz, 2H), 8.21 (d, *J* = 7.6 Hz, 2H), 7.90 (t, *J* = 5.9 Hz, 2H), and 3.36 (s, 3H). ESI–MS: [L^10^+H^+^]^+^: (388.11, 19%), [ZnL^10^–H^+^]^+^: (452.04, 10%), [ZnClL^10^]^+^: (486.00, 100%). IR (KBr, cm^–1^): 3104 (w, ν_C–H_), 3077 (w, ν_C–H_), 2988(w, ν_C–H_), 2906 (w, ν_C–H_), 1614 (m, ν_C=C_), 1598 (m, ν_C=C_), 1571 (m, ν_C=C_), 1551 (m, ν_C=C_), 1475 (s, ν_C=C_), 1444(w, δ_C–H_), 1428 (s, ν_C=C_), 1392 (s, ν_C=C_), 1290 (s, ν_SO2_), 1250 (m, β_C–H_), 1141 (s, ν_SO2_), 1093 (m, β_C–H_), 1013 (m, β_C–H_), 982 (s, γ_C–H_), 900 (m, γ_C–H_), 842 (m, γ_C–H_), 797 (s, γ_C–H_), and 776 (s, ν_S–O_), 737 (m, γ_C–H_), 676 (m), 659 (m), 641 (m), 553 (s, δ_SO2_), 532(s, δ_SO2_). Slow evaporation of its DMF solution led to colorless crystals that were suitable for X-ray analysis.

### 3.4. Crystallography

Single crystals of **2**–**5**, **9**, and **10** were mounted on glass fibers. Intensity data were collected while using an Agilent SuperNova diffractometer diffractometer (for compounds **2**–**4** and **9**) or a Bruker AXS-KAPPA APEX II diffractometer (for compounds **5** and **10**) with graphite monochromated Mo-Kα (λ 0.71073) radiation. The data were collected using omega scans of 0.5° per frame and full sphere of data were obtained. For the former machine, the cell parameters were retrieved and refined by Agilent CrysAlisPro software on all of the observed reflections [78]. For the latter one, the cell parameters were retrieved while using Bruker SMART software and refined using Bruker SAINT on all of the observed reflections [79]. Absorption corrections were applied while using SADABS [80]. The structures were solved by direct methods using the SHELXS–97 package and refined with SHELXL–97 [81]. Calculations were performed while using the WinGX System–Version 1.80.03 [82]. The hydrogen atoms were inserted in calculated positions. Least square refinements with anisotropic thermal motion parameters for all of the non–hydrogen atoms and isotropic for the remaining atoms were employed. CCDC 1568387–1568392, for compounds **2**–**5**, **9**, and **10** contain the supplementary crystallographic data of this paper. These data can be obtained free of charge from the Cambridge Crystallographic Data Centre via www.ccdc.cam.ac.uk/data_request/cif. Crystal data and details of data collections are reported in Table 1.

### 3.5. Solution Chemistry

The stability of the compounds has been analyzed by a DU 800 UV–VIS spectrophotometer (Beckman Coulter, Fullerton, CA). The solutions of the compounds in PBS (pH 7.4) were prepared while using DMSO stock solutions (2 mM), and the spectra were then measured at different time intervals over 72 h at 37 °C.

### 3.6. Antiproliferative Activity In Vitro

Five different cell lines, human lung carcinoma cell line (A549), human hepatocellular carcinoma cell line (Bel-7402), human breast cancer cell line (MCF-7), human esophageal squamous carcinoma cell line (Eca-109), and mouse monocyte macrophage cell line (RAW 264.7) purchased from American Type Culture Collection (ATCC), were used to evaluate the antiproliferative activity of the synthesized compounds. All of the cells were cultured with a completed DMEM medium supplemented with 10% fetal bovine serum, 100 U/mL penicillin, and 100 U/mL streptomycin in a humidified atmosphere at 37 °C with 5% CO_2_. The cells were seeded in 96-well plates with 3000 cells per well. 16 h later, series concentrations of compounds **1**–**10** were added into the predefined wells and then incubated for 72 h. Cell morphology was observed and imaged with an inverted microscope (Nikon eclipses TS100) with a Nikon digital camera (DXM 1200F). The cell viability was measured while using CCK-8 (cell counting kit-8, Beyotime Biotechnology, China) or MTT (3-(4,5-dimethylthiazol-2-yl)-2,5-diphenyl tetrazolium bromide) assay following the manufacturer’s instructions. The OD value was measured by using a multifunctional microplate reader (Infinite 200 PRO, TECAN, Switzerland) at 490 nm for the CCK-8 assay or 570 nm for MTT assay and the percentages of cell viability were determined. The cytotoxicity was evaluated based on the percentage cell survival in a dose-dependent manner relative to the negative control. GraphPad Prism V5.0 for windows (Graphpad Software, San Diego, CA, USA) was used to calculate the 50% inhibitive concentration (IC_50_). All the data are shown as the mean value ± S.D. of four independent experiments for the dose–response curves.

### 3.7. DNA Binding Studies

#### 3.7.1. UV-vis Absorption Studies

The UV-vis spectra were measured on a DU 800 UV-VIS spectrophotometer (Beckman Coulter, Fullerton, CA) in 5 mM Tris-HCl, 50 mM NaCl buffer (pH 7.2). Spectroscopic titrations were carried out at room temperature to determine the binding affinity between the DNA and Zn(II) complexes. Initially, the solutions of the blank buffer and Zn(II) complex sample were placed in the reference and sample cuvettes (1 cm path length), respectively, and first spectrum was then recorded in the range of 230–400 nm. During the titration, aliquot of buffered DNA solution was added to each cuvette to eliminate the absorbance of DNA itself, and repeated inversion was used to mix the solutions. The absorption spectra were recorded after the solutions were mixed for 10 min. The titration processes were repeated until there was no change in the spectra for four titrations at least, which indicated that binding saturation had been achieved. The changes in the metal complex concentration were negligible due to dilution at the end of each titration [83].

#### 3.7.2. Fluorescence Studies

Fluorescence measurements were made using Hitachi FL-7000 spectrophotometers with a slit width 5 nm for the excitation and emission beams. The fluorescence titrations were carried out by adding increasing amounts of CT-DNA directly into the cell containing the solution of Zn(II) complex in 5 mM Tris-HCl, 50 mM NaCl buffer (pH 7.2). The concentration range of the DNA was 0–120 μM. Emission spectra were recorded while using different excitation wavelengths for compounds **1**–**10** of 285 nm, 313 nm, 287 nm, 325 nm, 330 nm, 284 nm, 313 nm, 289 nm, 289 nm, and 285 nm, respectively. All of the measurements were performed at room temperature.

#### 3.7.3. Circular Dichroism (CD) Spectropolarimetry

Circular dichroism (CD) spectra (differential absorption of left and right circularly polarized light) of CT-DNA, ds(AT)_6_ and ds(CG)_6_ in the presence or absence of compounds **1**–**10** at different concentrations were recorded while using a Chirascan spectropolarimeter (Applied Photophysics, UK). The CD measurements were carried out using 2 mm Suprasil quartz cells from Hellma Analytics and maintained at a temperature of 20 °C using a TC125 temperature controller from Quantum Northwestern running on the Chirascan spectrophotometer. The spectra were recorded between 220 and 500 nm, with a bandwidth of 1 nm, time per point of 1 s. The spectra of 5 mM Tris-HCl and 50 mM NaCl buffer (pH 7.2) were used as the baselines and they were automatically subtracted from the CD spectra of the samples.

### 3.8. Molecular Docking

Molecular docking is a powerful method for predicting the orientation of ligand when binding to receptor. The coordination sphere of the Zn(II) complex was generated from its X-ray crystal structure as a CIF file. Subsequently, the CIF file was converted to the PDB format using Mercury software (http://www.ccdc.cam.ac.uk/). The three-dimensional structures of B-DNA dodecamer d(CGCGAATTCGCG)_2_ (PDB ID: 1BNA), ds(ATGCAT)_2_ (PDB ID: 4JD8), ds(ATAT)_2_ (PDB ID: 2DA8M), and ds(CGCGCGGC)_2_ (PDB ID: 2ROUM) were obtained and modified from the protein Data Bank (https://www.rcsb.org/). Molecular docking study was performed with Autodock 4.2.6 software while using the implemented empirical free energy function and the Lamarckian Genetic Algorithm [73]. The structures of the receptors were kept rigid during the docking, while the metal complex was allowed to have rotatable bonds. Prior to perform docking, all of the water molecules were charged and polar hydrogen atoms were added. The size of the grid was set to 60 Å × 60 Å × 160 Å or 60 Å × 60 Å × 80 Å with a spacing of 0.375 Å. All of the other parameters were kept as default. Amongst them, the conformation having the lowest energy was selected to depict the mode of interaction between the complexes and DNA. The results were visualized while using the PyMol Molecular Viewer package [84,85].

## 4. Conclusions

Various physicochemical and spectroscopic methods, including single crystal X-ray diffraction, have been used to synthesize and fully characterize a series of Zn(II) terpyridine complexes. All of the compounds display interesting photoluminescent properties with strong emission. The cytotoxicity assay in vitro indicates that the compounds have excellent anticancer activity against the four human carcinoma cell lines, with the lowest IC_50_ values of **10** against A549 (0.33 μM), **6** against Bel-7402 (0.66 μM), **7** against MCF-7 (0.37 μM), and **7** against Eca-109 (1.05 μM). The intensity of fluorescence emission peaks remarkably decreases when CT-DNA is gradually added to the solutions of the compounds. The results of electronic absorption, fluorescence titration, and circular dichroism spectroscopy show that the ten compounds have strong affinity to bind with DNA. The CD spectra show that the secondary structure of DNA is changed by the addition of the compounds, which results in the DNA conformational transition. Molecular docking studies further reveal that the compounds intercalate into the base pairs of DNA and such a binding is stabilized through π…π stacking and hydrogen bonding between DNA base pairs and the metal complexes. An analysis of the interactions suggests the order of nucleotide sequence binding selectivity as ATGC > ATAT > GCGC. The results of molecular docking confirm the results of the CD spectra and further clarify their origin. The intercalation between the compounds and DNA base pairs interrupts the replication and transcription of DNA for inducing cell apoptosis, playing an important role in their anticancer mechanism.

A global interpretation of the results shows that the determined properties, including photoluminescence, antiproliferative activity, and DNA binding mode, depend on the nature of the substituents at the tridentate terpyridine ligands. It is very meaningful to continue the study on interaction of these compounds with the DNA of tumor cells and carry out photocytotoxicity experiments to confirm their potential values as photodynamic therapy drugs.

## Figures and Tables

**Table 1:** Crystal data for compounds **2**–**5, 9**, and **10**.

Table. *Cont.*	2	3	4	5	9	10
Empirical formula	C_22_H_17_Cl_2_N_3_Zn	C_22_H_17_Cl_2_N_3_OZn	C_27_H_19_Cl_2_N_3_Zn	C_32_H_33_Cl_2_N_3_O_2_S_2_Zn	C_22_H_19_Cl_2_N_3_O_4_Zn	C_22_H_14_Cl_2_N_3_O_2_SZn
Formula weight	459.66	475.66	521.72	692.02	525.69	520.69
Temperature	298(2) K	298(2) K	298(2) K	298(2) K	298(2) K	298(2) K
Crystal system	Monoclinic	Monoclinic	Orthorhombic	Triclinic	Monoclinic	Monoclinic
space group	*P*2_1_*/c*	*P*2_1_*/c*	*Pbcn*	*P*-1	*I*2/*a*	*P*2_1_/*n*
*a* (Å)	12.6962(6)	8.5597(17)	12.025(2)	10.3442(12)	11.065(2)	10.3908(5)
*b* (Å)	9.5863(3)	14.455(3)	17.748(4)	12.2318(13)	17.610(3)	18.0058(9)
*c* (Å)	17.4340(9)	16.558(3)	11.024(2)	13.2856(14)	11.531(6)	12.7990(7)
*α* (°)	90	90	90	97.470(5)	90	90
*β* (°)	111.229(5)	99.10(3)	90	95.354(6)	107.31(3)	111.638(2)
*γ* (°)	90	90	90	97.459(5)	90	90
Volume (Å^3^)	1977.90(15)	2023.0(7)	2352.7(8)	1642.2(3)	2145.1(12)	2225.9(2)
*Z*	4	4	4	2	4	4
Calculated desity(Mg/m^3^)	1.544	1.562	1.473	1.399	1.628	1.554
Absorption coefficient(mm^−1^)	1.525	1.497	1.292	1.071	1.430	1.462
*F*(000)	936	968	1064	716	1072	1052
Crystal size(mm^−1^)	0.49 × 0.48 × 0.29	0.48 × 0.46 × 0.34	0.48 × 0.36 × 0.27	0.43 × 0.28 × 0.25	0.48 × 0.46 × 0.34	0.39 × 0.22 × 0.21
*θ*_max_, *θ*_min_ (°)	27.50, 2.74	27.49, 2.86	29.61, 2.95	27.92, 2.13	29.59, 3.22	29.60, 2.05
Index range *h*	−16 → 16	−11 → 9	−16 → 15	−13 → 13	−8 → 14	−14 → 13
*K*	−10 → 12	−17 → 18	−24 → 22	−15 → 16	−22 → 20	−24 → 20
*L*	−15 → 22	−20 → 21	−14 → 14	−17 → 17	−15 → 14	−17 → 17
Reflections collected/unique	16,461/4492 [*R*(int) = 0.0181]	13,327/4516 [*R*(int) = 0.0265]	23,550/3051 [*R*(int) = 0.0273]	24,876/7674 [*R*(int) = 0.0222]	5545/2558 [*R*(int) = 0.0188]	23,202/6085 [*R*(int) = 0.0472]
Data/restraints/parameters	4492/0/253	4516/0/262	3051/0/153	7674/0/389	2558/0/148	6085/0/280
Goodness-of-fit on *F*^2^	1.032	1.019	1.038	1.027	1.046	1.008
Final *R* indices [*I* > 2σ(*I*)]	*R*1 = 0.0254, *wR*2 = 0.0717	*R*1 = 0.0324, *wR*2 = 0.0805	*R*1 = 0.0426, *wR*2 = 0.1298	*R*1 = 0.0407, *wR*2 = 0.1151	*R*1 = 0.0430, *wR*2 = 0.1268	*R*1 = 0.0582, *wR*2 = 0.1600
*R* indices (all data)	*R*1 = 0.0311, *wR*2 = 0.0756	*R*1 = 0.0422, *wR*2 = 0.0861	*R*1 = 0.0528, *wR*2 = 0.1414	*R*1 = 0.0494, *wR*2 = 0.1220	*R*1 = 0.0490, *wR*2 = 0.1355	*R*1 = 0.1109, *wR*2 = 0.1844
Largest diff. peak and hole(e Å^−3^)	0.306 and −0.375	0.324 and −0.473	1.169 and −0.440	1.486 and −0.651	0.372 and −0.893	0.828 and −0.442
CCDC number	1,568,389	1,568,387	1,568,390	1,568,391	1,568,392	1,568,388

**Table 2:** Emission bands of compounds **1**–**10** in solid state and DMF solution at room temperature.

Compound	Solid State			DMF Solution		
	Excitation (nm)	Emission Bands (nm)		Excitation (nm)	Emission Bands (nm)	
1	300^a^	409^a^		270	370	509
2	300	412		350	413	504
3	300	420	533	380	443	513
4	300	417		360	438	
5	300	461		300	460	
6	330^b^	420^b^		470	528	
7	300^b^	415^b^		370	510	
8	360^b^	483^b^		380	464	
9	300	381	400	270	371	513
10	300	385		310	375	511

^a^ Emission data was cited from reference [41]. ^b^ Emission data was cited from reference [42].

**Table 3:** IC_50_ values (μM) from the dose-response assay of compounds **1**–**10** and the reference compound cisplatin in the A549, Bel-7402, MCF-7, and Eca-109 cell lines, after an incubation time of 72 h.

Compound	A549		Bel-7402		MCF-7		Eca-109	
	IC_50_ (μM)	95% Confidence Intervals (μM)	IC_50_ (μM)	95% Confidence Intervals (μM)	IC_50_ (μM)	95% Confidence Intervals (μM)	IC50 (μM)	95% Confidence Intervals (μM)
**1**	0.440	0.384–0.504	1.309	1.098–1.560	1.486	0.855–2.582	1.251	1.024–1.528
**2**	0.933	0.821–1.061	1.842	1.741–1.949	2.769	1.584–4.840	2.017	1.507–2.701
**3**	0.756	0.672–0.852	1.470	1.202–1.797	1.358	0.911–2.025	1.821	1.475–2.247
**4**	1.042	0.858–1.265	1.883	1.691–2.096	0.589	0.336–1.035	3.320	2.996–3.680
**5**	0.586	0.476–0.722	1.435	1.150–1.790	1.187	0.655–2.152	1.722	1.360–2.181
**6**	0.435	0.370–0.513	0.660	0.447–0.975	1.956	1.148–3.332	1.198	0.908–1.580
**7**	0.633	0.568–0.705	1.636	1.459–1.835	0.374	0.229–0.609	1.045	0.862–1.266
**8**	1.228	0.938–1.608	1.557	1.396–1.736	2.428	1.870–3.153	1.215	0.852–1.732
**9**	1.270	1.165–1.385	1.804	1.521–2.139	3.548	3.016–4.173	1.280	0.985–1.665
**10**	0.333	0.279–0.396	0.730	0.604–0.883	1.764	0.999–3.116	1.193	0.841–1.692
Cisplatin	5.082	4.639–5.568	3.088	2.856–3.340	11.49	9.435–14.00	11.99	10.79–13.32

**Table 4:** Binding constants *K*_b_ values of compounds **1**–**10** interacting with CT-DNA (calf thymus DNA).

Compound	*K*_b_ (M^−1^)	Log *K*_b_	*R^a^*
**1**	6.89 × 10^2^	2.84	0.9949
**2**	4.78 × 10^4^	4.68	0.9830
**3**	9.95 × 10^1^	0.00	0.9949
**4**	3.96 × 10^4^	4.60	0.9923
**5**	9.79 × 10^4^	4.99	0.9898
**6**	1.36 × 10^4^	4.13	0.9973
**7**	1.86 × 10^4^	4.27	0.9830
**8**	8.55 × 10^4^	4.93	0.9911
**9**	1.08 × 10^4^	4.03	0.9830
**10**	3.49 × 10^3^	3.54	0.9987

*^a^ R* is the correlation coefficient for the *K*_b_ values.

**Table 5:** The calculated free energy of binding of the compounds **1**–**10** with B-DNA (1BNA), ds(ATGCAT)_2_ (4JD8), ds(ATAT)_2_ (2DA8M), and ds(CGCGCG)_2_ (2ROUM).

Compound	Affinity (kcal/mol)			
	1BNA	4JD8	2DA8M	2ROUM
**1**	−9.09	−8.43	−8.30	−8.13
**2**	−9.63	−8.88	−8.42	−8.16
**3**	−9.20	−8.21	−7.49	−8.00
**4**	−11.43	−8.77	−8.94	−8.71
**5**	−11.83	−9.14	−8.90	−8.83
**6**	−9.56	−8.40	−8.21	−7.92
**7**	−9.32	−8.64	−8.50	−8.28
**8**	−9.29	−8.85	−8.12	−8.60
**9**	−8.70	−7.71	−7.74	−7.38
**10**	−10.83	−8.65	−8.33	−8.35

**Table 6:** Hydrogen bond interactions for compounds **3** and **6**–**10**.

Compound	DNA	Bonds Formed	Bond Distance (Å)	Bond Energy (kcal/mol)
3	1BNA	N–H…O (DG4)	1.877	−0.818
6	1BNA	O–H…O (DA18)	2.047	−5.96
	4JD8	N–H…O (DA5)	2.065	−5.551
	2DA8M	N–H…O (DT5)	2.113	−5.749
	2ROUM	N–H…O (DT2)	1.946	−3.502
7	1BNA	O–H…O (DT19)	2.164	−5.727
	4JD8	O–H…O (DT2)	2.229	−0.263
	2DA8M	O–H…O (DT5)	1.820	−1.640
	2ROUM	O–H…O (DC7)	1.975	−5.769
8	1BNA	O–H…O (DT19)	1.828	−4.119
	2DA8M	N–H…O (DT3)	1.862	−4.078
	2ROUM	N–H…O (DC7)	2.210	−3.302
9	1BNA	N–H…O (DG16)	1.837	−7.674
10	1BNA	N–H…O (DG16)	1.765	−7.869
	2DA8M	O–H…O (DA4)	2.115	−4.156

## Supplementary Materials

The following are available online, Figures S1–S6: ^1^H NMR spectrum of compounds **2**, **3**, **4**, **5**, **9** and **10** in DMSO, Figures S7–S12: The ESI-MS spectra of compounds **2**, **3**, **4**, **5**, **9** and **10**, Figures S13–S18: The IR spectra of compounds **2**, **3**, **4**, **5**, **9** and **10**, Figures S19–S28: UV-vis spectra of compounds **1**–**10** for a period of 72 h, Figures S29–S31: The microscopic photographs of the A549, the MCF-7 and the Eca-109 cancer cells treated with increased concentrations of the compounds **1**–**9** and cisplatin at magnification of 200×, Figures S32–S35: The plots of the cell viability vs. the concentration of compounds 1–10 against Bel-7402, MCF-7, Eca-109 and RAW 264.7 cell lines, Figure S36: UV-vis spectra for compounds **1**–**10** in Tris-HCl 1.0 × 10^−3^ (pH 7.2) with increasing the concentration of CT-DNA, and the inset show plots of A_0_/(A – A_0_) versus the concentration of CT-DNA. Figures S37 and S38: Circular dichroism spectra of three kinds of DNA in the presence or absence of compounds **2**–**5** and **7**–**10** in Tris-HCl buffer (pH 7.2), at 20 °C, Figure S39: The most favorable orientation of (A): compound 1, (B): compound 2 and (C): compound 4 bound with the minor groove of the CT-DNA, Figures S40–S45: The most favorable orientation of compounds **3** and **6**–**10** bound with the minor groove of the B-DNA (PDB ID: 1BNA), Figures S46–S54: View of the energy minimized docked poses of compounds **1**–**10** with (A) ds(ATGCAT)2 (PDB ID: 4JD8), (B) ds(ATAT)2 (PDB ID: 2DA8M), and (C) ds(CGCGCG)2 (PDB ID: 2ROUM).
