# Supplementary material for: Zinc(II) Terpyridine Complexes: Substituent Effect on Photoluminescence, Antiproliferative Activity, and DNA Interaction
Source: Molecules. 2019 Dec 10;24(24):4519. doi: 10.3390/molecules24244519 (PMC6943603; doi:10.3390/molecules24244519)

checkCIF/PLATON report

Structure factors have been supplied for datablock(s) I

THIS REPORT IS FOR GUIDANCE ONLY. IF USED AS PART OF A REVIEW PROCEDURE FOR PUBLICATION, IT SHOULD NOT REPLACE THE EXPERTISE OF AN EXPERIENCED CRYSTALLOGRAPHIC REFEREE.

No syntax errors found. CIF dictionary Interpreting this report

Datablock: I

```
Bond precision:  C-C = 0.0059 A          Wavelength=0.71073

Cell:            a=10.3908(5)    b=18.0058(9)        c=12.7990(7)
                 alpha=90        beta=111.638(2)     gamma=90
Temperature:     298 K

                Calculated                  Reported
Volume          2225.9(2)                   2225.9(2)
Space group     P 21/n                      P 21/n
Hall group      -P 2yn                      -P 2yn
Moiety formula  C22 H14 Cl2 N3 O2 S Zn      C22 H14 Cl2 N3 O2 S Zn
Sum formula     C22 H14 Cl2 N3 O2 S Zn      C22 H14 Cl2 N3 O2 S Zn
Mr              520.71                      520.69
Dx,g cm-3       1.554                       1.554
Z               4                           4
Mu (mm-1)       1.462                       1.462
F000            1052.0                      1052.0
F000'           1055.10
h,k,lmax        14,25,17                    14,24,17
Nref            6258                        6085
Tmin,Tmax       0.687,0.736                 0.638,0.746
Tmin'           0.560

Correction method= # Reported T Limits: Tmin=0.638 Tmax=0.746
AbsCorr = MULTI-SCAN

Data completeness= 0.972          Theta(max)= 29.595

R(reflections)= 0.0581( 3548)     wR2(reflections)= 0.1844( 6085)

S = 1.008                  Npar= 280
```

The following ALERTS were generated. Each ALERT has the format
test-name_ALERT_alert-type_alert-level.
Click on the hyperlinks for more details of the test.

Alert level C

```
PLAT906_ALERT_3_C Large K value in the Analysis of Variance ......      2.057 Check
PLAT911_ALERT_3_C Missing # FCF Refl Between THmin & STh/L=  0.600         33 Report
```

Alert level G

```
PLAT072_ALERT_2_G SHELXL First  Parameter in WGHT  Unusually Large       0.10 Report
PLAT343_ALERT_2_G Unusual sp?      Angle Range in Main Residue for        C22 Check
PLAT899_ALERT_4_G SHELXL97   is Deprecated and Succeeded by SHELXL       2014 Note
PLAT912_ALERT_4_G Missing # of FCF Reflections Above STh/L=  0.600        141 Note
PLAT978_ALERT_2_G Number C-C Bonds with Positive Residual Density.          1 Note
```

0 **ALERT level A** = Most likely a serious problem - resolve or explain
0 **ALERT level B** = A potentially serious problem, consider carefully
2 **ALERT level C** = Check. Ensure it is not caused by an omission or oversight
5 **ALERT level G** = General information/check it is not something unexpected

0 ALERT type 1 CIF construction/syntax error, inconsistent or missing data
3 ALERT type 2 Indicator that the structure model may be wrong or deficient
2 ALERT type 3 Indicator that the structure quality may be low
2 ALERT type 4 Improvement, methodology, query or suggestion
0 ALERT type 5 Informative message, check

checkCIF publication errors

Alert level A

```
PUBL008_ALERT_1_A  _publ_section_title is missing. Title of paper.
PUBL009_ALERT_1_A  _publ_author_name is missing. List of author(s) name(s).
PUBL010_ALERT_1_A  _publ_author_address is missing. Author(s) address(es).
PUBL012_ALERT_1_A  _publ_section_abstract is missing.
            Abstract of paper in English.
```

Alert level G

```
PUBL017_ALERT_1_G The _publ_section_references section is missing or
            empty.
```

4 **ALERT level A** = Data missing that is essential or data in wrong format
1 **ALERT level G** = General alerts. Data that may be required is missing

Publication of your CIF

You should attempt to resolve as many as possible of the alerts in all categories. Often the minor alerts point to easily fixed oversights, errors and omissions in your CIF or refinement strategy, so attention to these fine details can be worthwhile. In order to resolve some of the more serious problems it may be necessary to carry out additional measurements or structure refinements. However, the nature of your study may justify the reported deviations from journal submission requirements and the more serious of these should be commented upon in the discussion or experimental section of a paper or in the "special_details" fields of the CIF. *checkCIF* was carefully designed to identify outliers and unusual parameters, but every test has its limitations and alerts that are not important in a particular case may appear. Conversely, the absence of alerts does not guarantee there are no aspects of the results needing attention. It is up to the individual to critically assess their own results and, if necessary, seek expert advice.

If level A alerts remain, which you believe to be justified deviations, and you intend to submit this CIF for publication in a journal, you should additionally insert an explanation in your CIF using the Validation Reply Form (VRF) below. This will allow your explanation to be considered as part of the review process.

Validation response form

Please find below a validation response form (VRF) that can be filled in and pasted into your CIF.

```
# start Validation Reply Form
_vrf_PUBL008_GLOBAL
;
PROBLEM: _publ_section_title is missing. Title of paper.
RESPONSE: ...
;
_vrf_PUBL009_GLOBAL
;
PROBLEM: _publ_author_name is missing. List of author(s) name(s).
RESPONSE: ...
;
_vrf_PUBL010_GLOBAL
;
PROBLEM: _publ_author_address is missing. Author(s) address(es).
RESPONSE: ...
;
_vrf_PUBL012_GLOBAL
;
PROBLEM: _publ_section_abstract is missing.
RESPONSE: ...
;
# end Validation Reply Form
```

If you wish to submit your CIF for publication in Acta Crystallographica Section C or E, you should upload your CIF via the web. If you wish to submit your CIF for publication in IUCrData you should upload your CIF via the web. If your CIF is to form part of a submission to another IUCr journal, you will be asked, either during electronic submission or by the Co-editor handling your paper, to upload your CIF via our web site.

PLATON version of 27/03/2017; check.def file version of 24/03/2017

Datablock I - ellipsoid plot

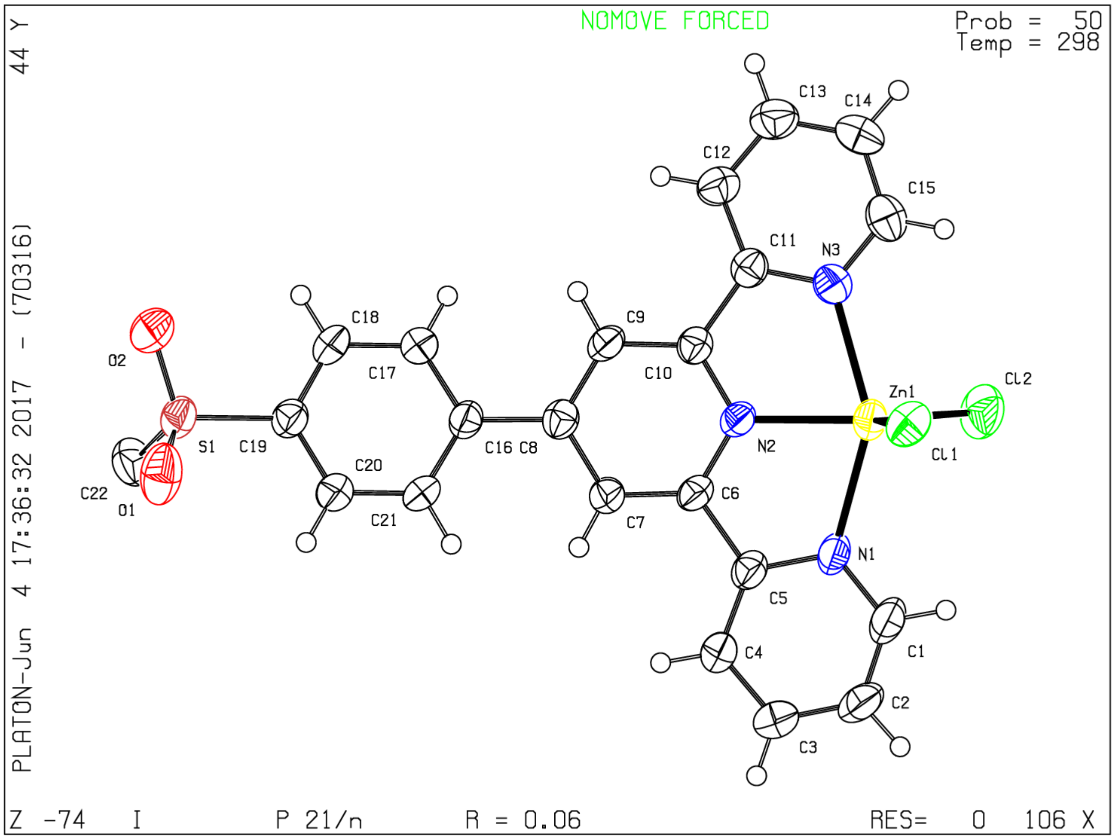

Supplement: Supplementary file 1 [file molecules-24-04519-s001.zip › Supplementary File/Checkcif_1568388 (compound 10).pdf]
