# Supplementary material for: Zinc(II) Terpyridine Complexes: Substituent Effect on Photoluminescence, Antiproliferative Activity, and DNA Interaction
Source: Molecules. 2019 Dec 10;24(24):4519. doi: 10.3390/molecules24244519 (PMC6943603; doi:10.3390/molecules24244519)
Supplement: Supplementary file 1 [file molecules-24-04519-s001.zip › Supplementary File/Molecules_Supporting Information.pdf]

Supporting Information for

Zinc(II) terpyridine complexes: substituent effect on photoluminescence, antiproliferative activity and DNA interaction

Jiahe Li [1,2], Rongping Liu [1], Jinzhang Jiang [1], Xing Liang [1], Ling Huang [1], Gang Huang [3], Hailan Chen [4,*], Lixia Pan [2,*] and Zhen Ma [1,2,*]

[1] School of Chemistry and Chemical Engineering, Guangxi University, Nanning 530004, Guangxi, People's Republic of China; lijiahe006@163.com(J.L.); 1152543513@qq.com(R.L.); jjz19941028@gmail.com(J.J.); liangxing587@126.com(X.L.); 441073854@qq.com(L.H.); mzmz2009@sohu.com(Z.M)

[2] Centro de Química Estrutural, Instituto Superior Técnico, Universidade de Lisboa, Av. Rovisco Pais, 1049-001, Lisbon, Portugal

[3] National Engineering Research Center for Non-Food Biorefinery, State Key Laboratory of Non-Food Biomass and Enzyme Technology, Guangxi Key Laboratory of Bio-refinery, Guangxi Biomass Engineering Technology Research Center, Guangxi Academy of Sciences, Nanning 530004, Guangxi, People’s Republic of China; wangyi.07@163.com; panlixia@gxas.cn

[4] School of Animal Science and Technology, Guangxi University, Nanning 530004, Guangxi, People's Republic of China; hlchen319@163.com

* Correspondence: hlchen319@163.com(H.C.); panlixia@gxas.cn(L.P.); mzmz2009@sohu.com(Z.M.)

INDEX

Figure S1 ^{1}H NMR spectrum of compound **2** in DMSO (600 MHz). S6

Figure S2 ^{1}H NMR spectrum of compound **3** in DMSO (600 MHz). S6

Figure S3 ^{1}H NMR spectrum of compound **4** in DMSO (600 MHz). S7

Figure S4 ^{1}H NMR spectrum of compound **5** in DMSO (600 MHz). S7

Figure S5 ^{1}H NMR spectrum of compound **9** in DMSO (600 MHz). S8

Figure S6 ^{1}H NMR spectrum of compound **10** in DMSO (600 MHz). S8

Figure S7 The ESI–MS spectrum of compound **2**. S9

Figure S8 The ESI–MS spectrum of compound **3**. S9

Figure S9 The ESI–MS spectrum of compound **4**. S9

Figure S10 The ESI–MS spectrum of compound **5**. S10

Figure S11 The ESI–MS spectrum of compound **9**. S10

Figure S12 The ESI–MS spectrum of compound **10**. S10

Figure S13 The IR spectrum of compound **2**. S11

Figure S14 The IR spectrum of compound **3**. S11

Figure S15 The IR spectrum of compound **4**. S11

Figure S16 The IR spectrum of compound **5**. S12

Figure S17 The IR spectrum of compound **9**. S12

Figure S18 The IR spectrum of compound **10**. S12

Figure S19 UV−vis spectra of compounds **1** for a period of 72 h. S13

Figure S20 UV−vis spectra of compounds **2** for a period of 72 h. S13

Figure S21 UV−vis spectra of compounds **3** for a period of 72 h. S13

Figure S22 UV−vis spectra of compounds **4** for a period of 72 h. S14

Figure S23 UV−vis spectra of compounds **5** for a period of 72 h. S14

Figure S24 UV−vis spectra of compounds **6** for a period of 72 h. S14

Figure S25 UV−vis spectra of compounds **7** for a period of 72 h. S15

Figure S26 UV−vis spectra of compounds **8** for a period of 72 h. S15

Figure S27 UV−vis spectra of compounds **9** for a period of 72 h. S15

Figure S28 UV−vis spectra of compounds **10** for a period of 72 h. S16

Figure S29 The microscopic photographs of the A549 cancer cell treated with increased concentrations of the compounds **1**–**9** and cisplatin at magnification of 200×..........S18

Figure S30 The microscopic photographs of the MCF-7 cancer cell treated with increased concentrations of the compounds **1**–**9** and cisplatin at magnification of 200×. S20

Figure S31 The microscopic photographs of the Eca-109 cancer cell treated with increased concentrations of the compounds **1**–**9** and cisplatin at magnification of 200×. S22

Figure S32 The plots of the cell viability *vs.* the concentration of compounds **1**–**10** against Bel-7402 cell line..........S23

Figure S33 The plots of the cell viability *vs.* the concentration of compounds **1**–**10** against MCF-7 cell line..........S23

Figure S34 The plots of the cell viability *vs.* the concentration of compounds **1**–**10** against Eca-109 cell line. S24

Figure S35 The plots of the cell viability *vs.* the concentration of compounds **1**–**10** against RAW 264.7 cell line. S24

Figure S36 UV–vis spectra for compounds **1**–**10** in Tris-HCl 1.0×10^{-3} (pH 7.2) with increasing the concentration of CT-DNA, and the inset show plots of $A_0/(A–A_0)$ versus the concentration of CT-DNA..........S26

Figure S37 Circular dichroism spectra of three kinds of DNA in the presence or absence of compounds **2**–**5** in Tris-HCl buffer (pH 7.2), at 20 °C. S27

Figure S38 Circular dichroism spectra of three kinds of DNA in the presence or absence of compounds **7**–**10** in Tris-HCl buffer (pH 7.2), at 20 °C. S28

Figure S39 The most favorable orientation of (A): compound **1**, (B): compound **2** and (C): compound **4** bound with the minor groove of the CT-DNA (PDB ID: 1BNA). S31

Figure S40 The most favorable orientation of compound **3** bound with the minor groove of the B-DNA (PDB ID: 1BNA)..........S31

Figure S41 The most favorable orientation of compound **6** bound with the minor groove of the B-DNA (PDB ID: 1BNA)..........S31

Figure S42 The most favorable orientation of compound **7** bound with the minor groove of the B-DNA (PDB ID: 1BNA)....S32
Figure S43 The most favorable orientation of compound **8** bound with the minor groove of the B-DNA (PDB ID: 1BNA)....S32
Figure S44 The most favorable orientation of compound **9** bound with the minor groove of the B-DNA (PDB ID: 1BNA)....S32
Figure S45 The most favorable orientation of compound **10** bound with the minor groove of the B-DNA (PDB ID: 1BNA)....S33
Figure S46 View of the energy minimized docked poses of compound **1** with (A) ds(ATGCAT)$_2$ (PDB ID: 4JD8), (B) ds(ATAT)$_2$ (PDB ID: 2DA8M), and (C) ds(CGCGCG)$_2$ (PDB ID: 2ROUM)....S34
Figure S47 View of the energy minimized docked poses of compound **2** with (A) ds(ATGCAT)$_2$ (PDB ID: 4JD8), (B) ds(ATAT)$_2$ (PDB ID: 2DA8M), and (C) ds(CGCGCG)$_2$ (PDB ID: 2ROUM)....S35
Figure S48 View of the energy minimized docked poses of compound **3** with (A) ds(ATGCAT)$_2$ (PDB ID: 4JD8), (B) ds(ATAT)$_2$ (PDB ID: 2DA8M), and (C) ds(CGCGCG)$_2$ (PDB ID: 2ROUM)....S36
Figure S49 View of the energy minimized docked poses of compound **4** with (A) ds(ATGCAT)$_2$ (PDB ID: 4JD8), (B) ds(ATAT)$_2$ (PDB ID: 2DA8M), and (C) ds(CGCGCG)$_2$ (PDB ID: 2ROUM)....S37
Figure S50 View of the energy minimized docked poses of compound **5** with (A) ds(ATGCAT)$_2$ (PDB ID: 4JD8), (B) ds(ATAT)$_2$ (PDB ID: 2DA8M), and (C) ds(CGCGCG)$_2$ (PDB ID: 2ROUM)....S38
Figure S51 View of the energy minimized docked poses of compound **6** with (A) ds(ATGCAT)$_2$ (PDB ID: 4JD8), (B) ds(ATAT)$_2$ (PDB ID: 2DA8M), and (C) ds(CGCGCG)$_2$ (PDB ID: 2ROUM)....S39
Figure S52 View of the energy minimized docked poses of compound **7** with (A) ds(ATGCAT)$_2$ (PDB ID: 4JD8), (B) ds(ATAT)$_2$ (PDB ID: 2DA8M), and (C) ds(CGCGCG)$_2$ (PDB ID: 2ROUM)....S40
Figure S53 View of the energy minimized docked poses of compound **8** with (A)

$ds(ATGCAT)_2$ (PDB ID: 4JD8), (B) $ds(ATAT)_2$ (PDB ID: 2DA8M), and (C) $ds(CGCGCG)_2$ (PDB ID: 2ROUM)..S41

Figure S54 View of the energy minimized docked poses of compound **9** with (A) $ds(ATGCAT)_2$ (PDB ID: 4JD8), (B) $ds(ATAT)_2$ (PDB ID: 2DA8M), and (C) $ds(CGCGCG)_2$ (PDB ID: 2ROUM)..S42

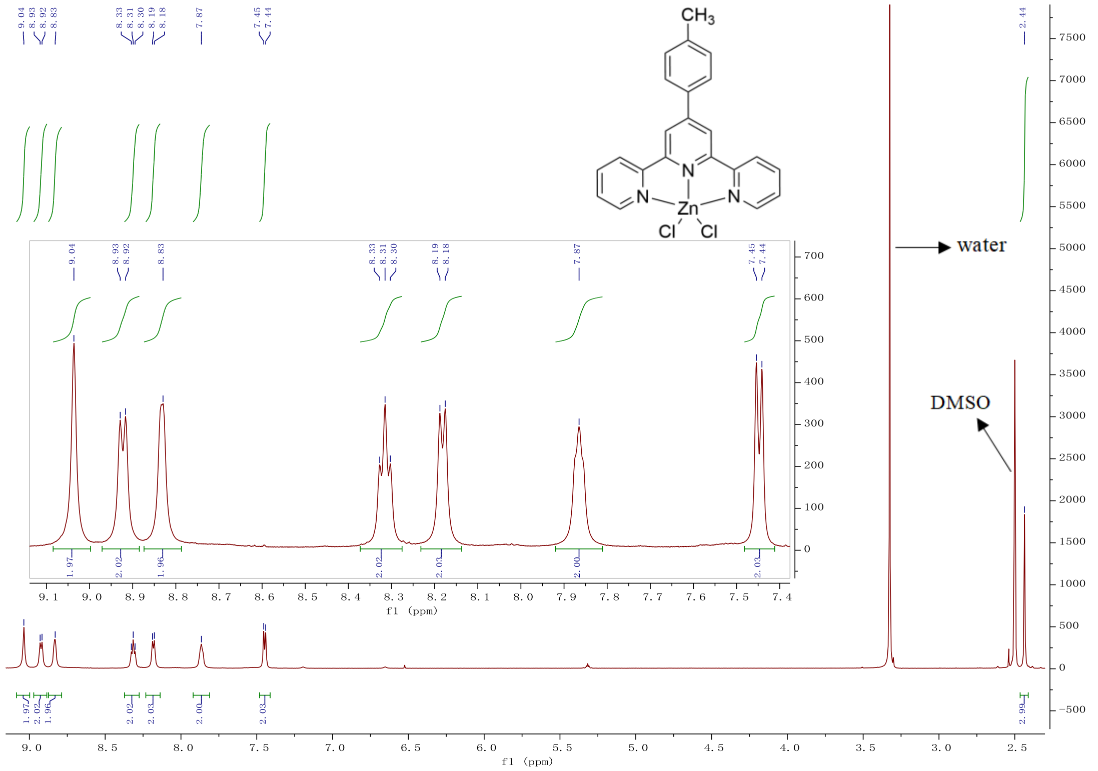

Figure S1 ^{1}H NMR spectrum of compound **2** in DMSO (600 MHz).

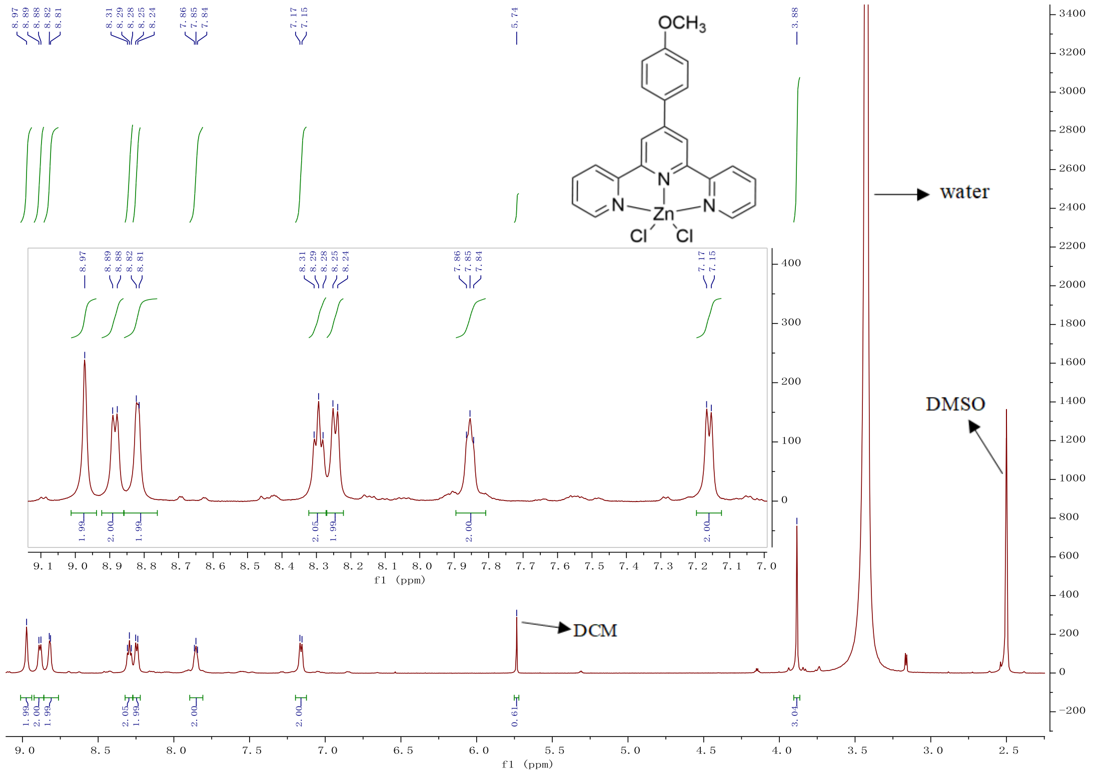

Figure S2 ^{1}H NMR spectrum of compound **3** in DMSO (600 MHz).

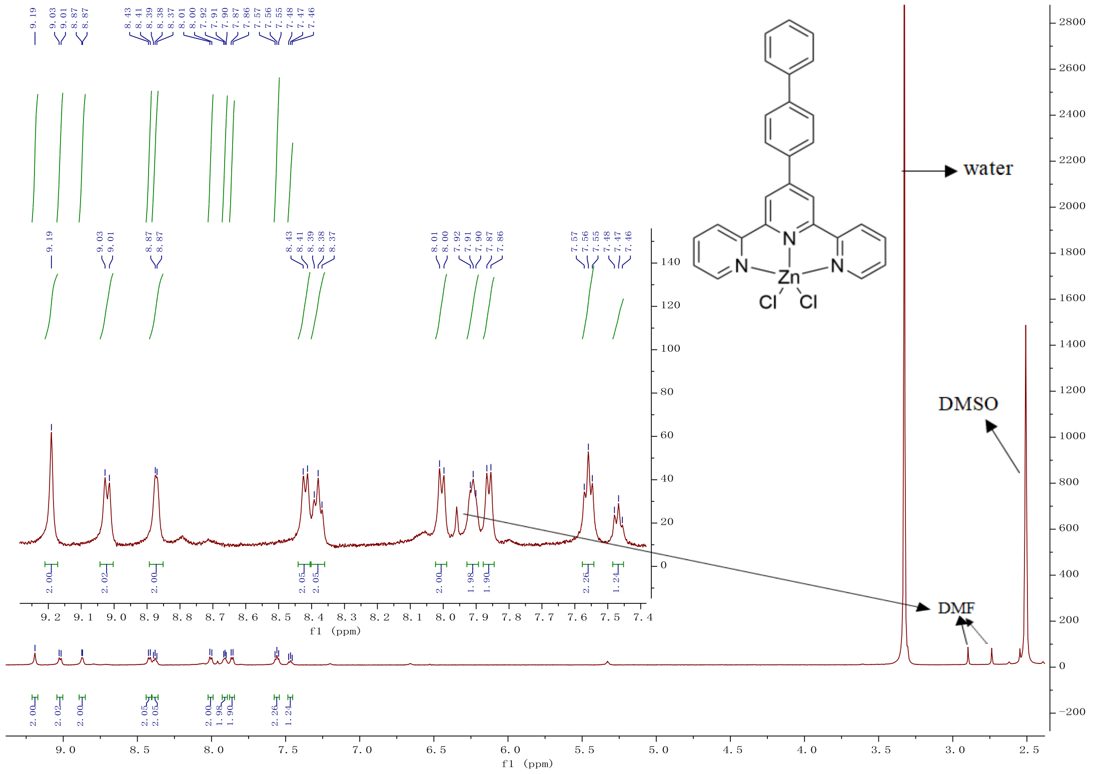

Figure S3 ^{1}H NMR spectrum of compound **4** in DMSO (600 MHz).

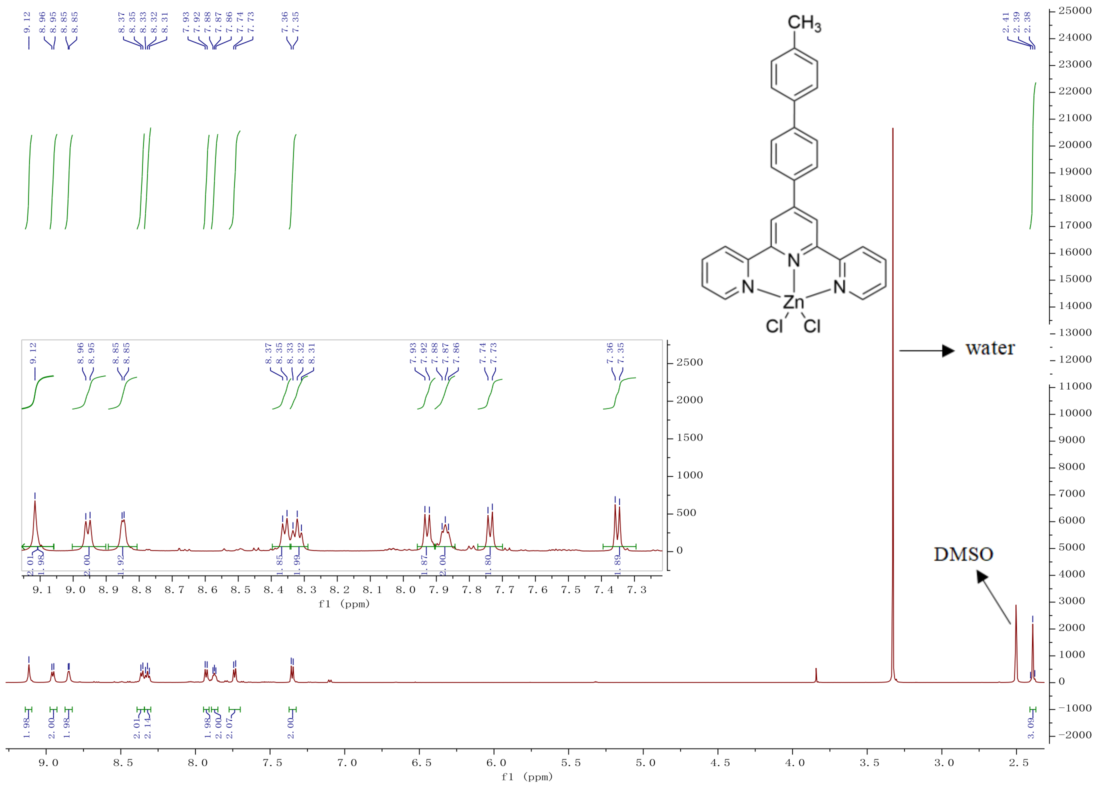

Figure S4 ^{1}H NMR spectrum of compound **5** in DMSO (600 MHz).

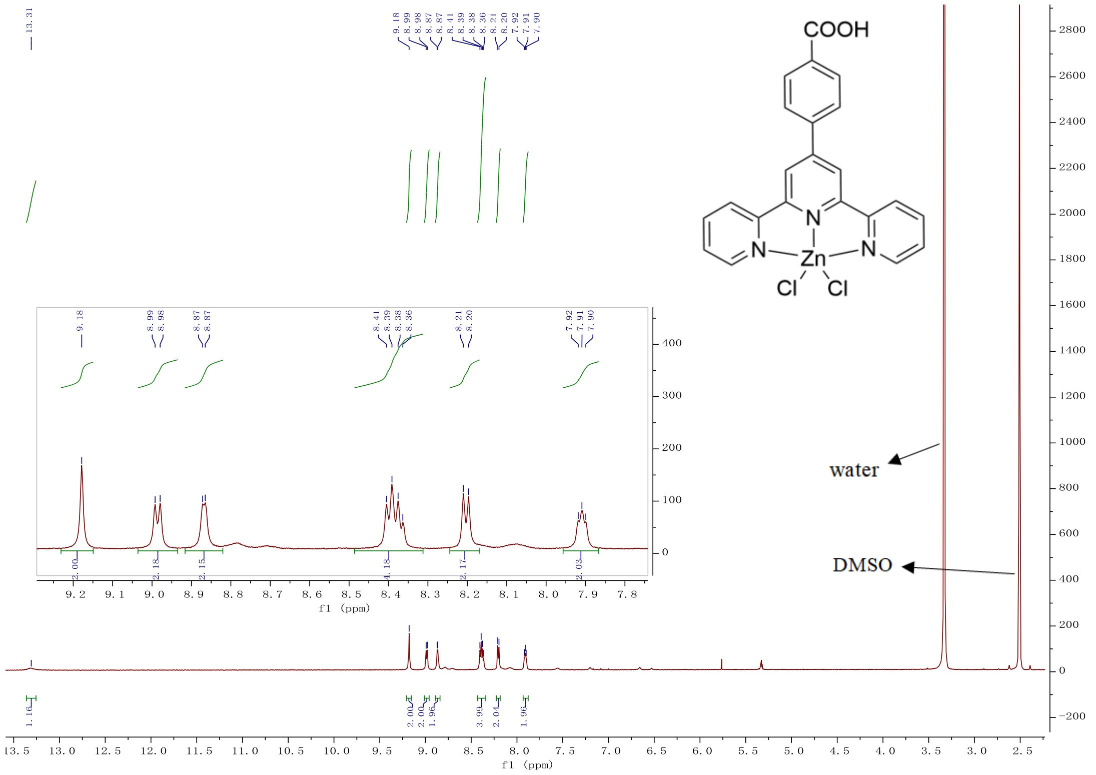

Figure S5 ^{1}H NMR spectrum of compound **9** in DMSO (600 MHz).

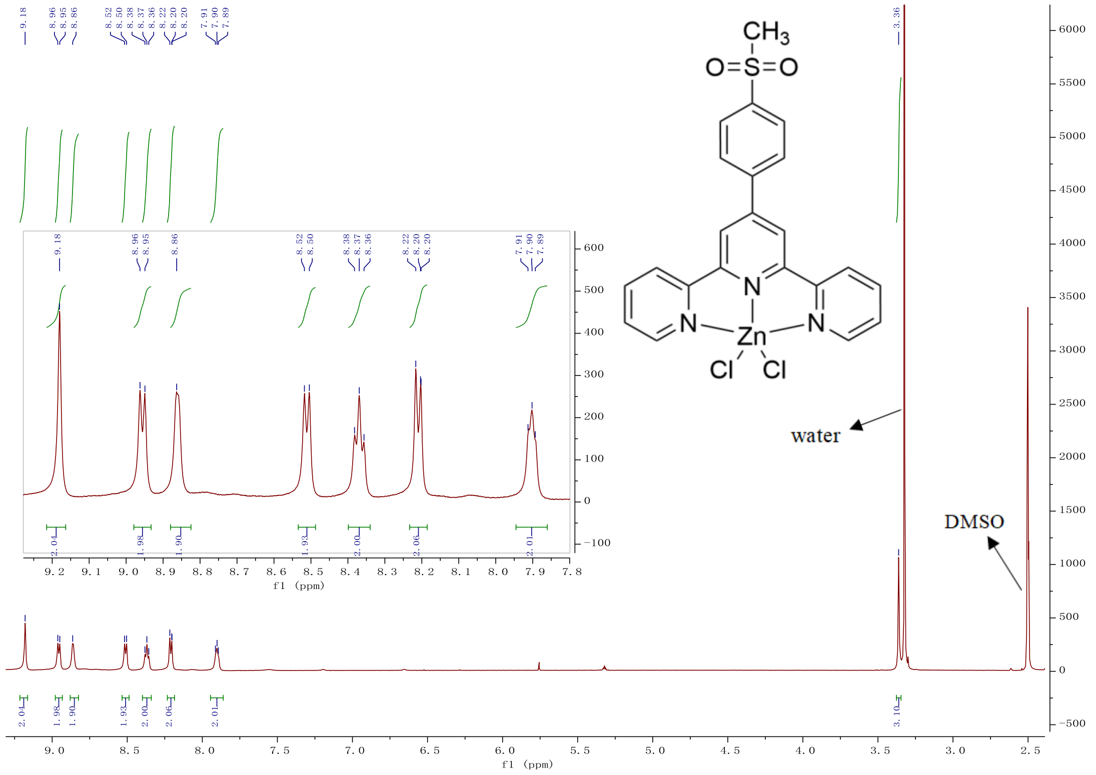

Figure S6 ^{1}H NMR spectrum of compound **10** in DMSO (600 MHz).

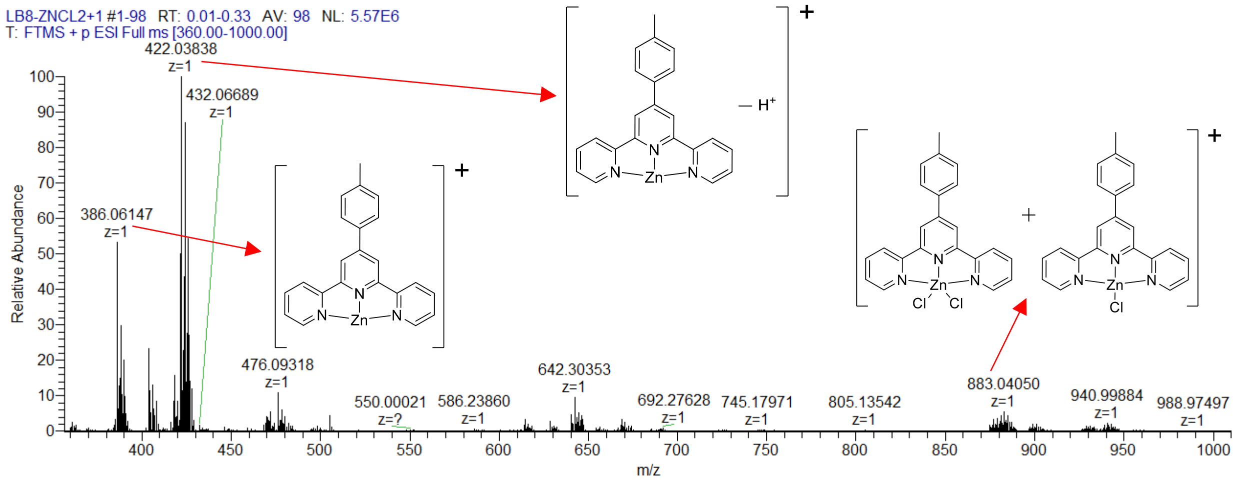

Figure S7 The ESI–MS spectrum of compound **2**.

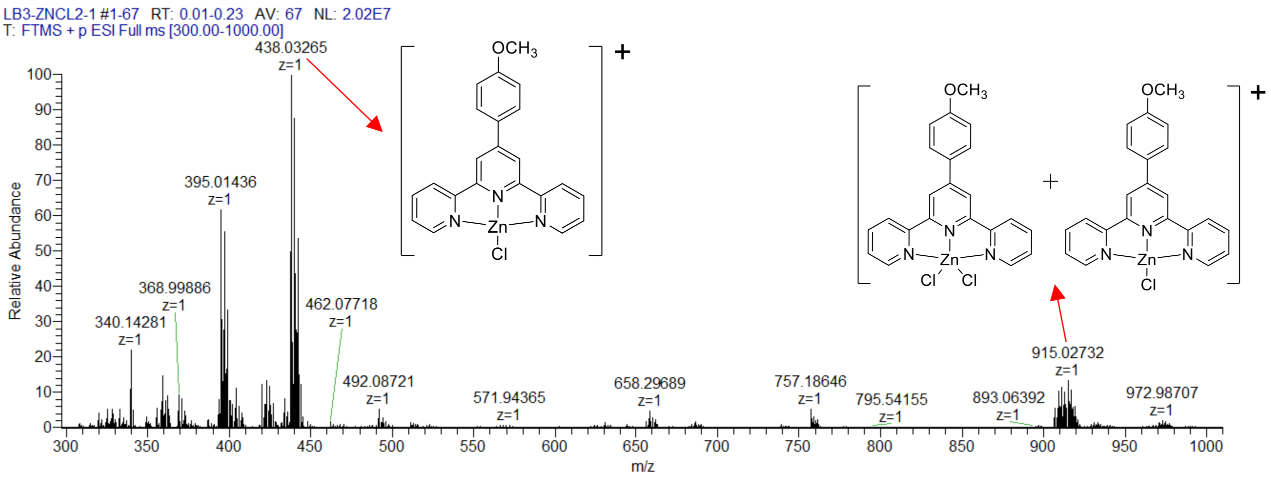

Figure S8 The ESI–MS spectrum of compound **3**.

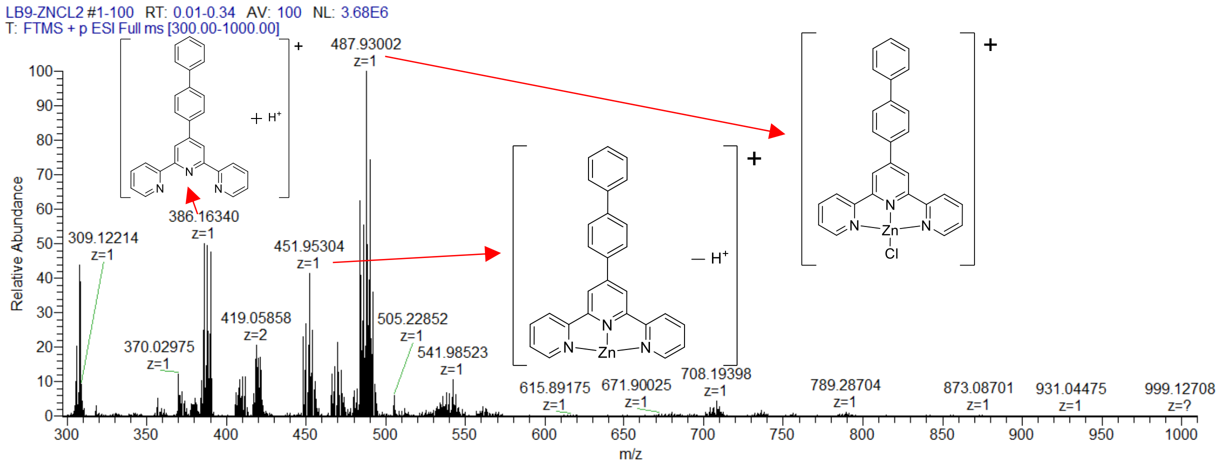

Figure S9 The ESI–MS spectrum of compound **4**.

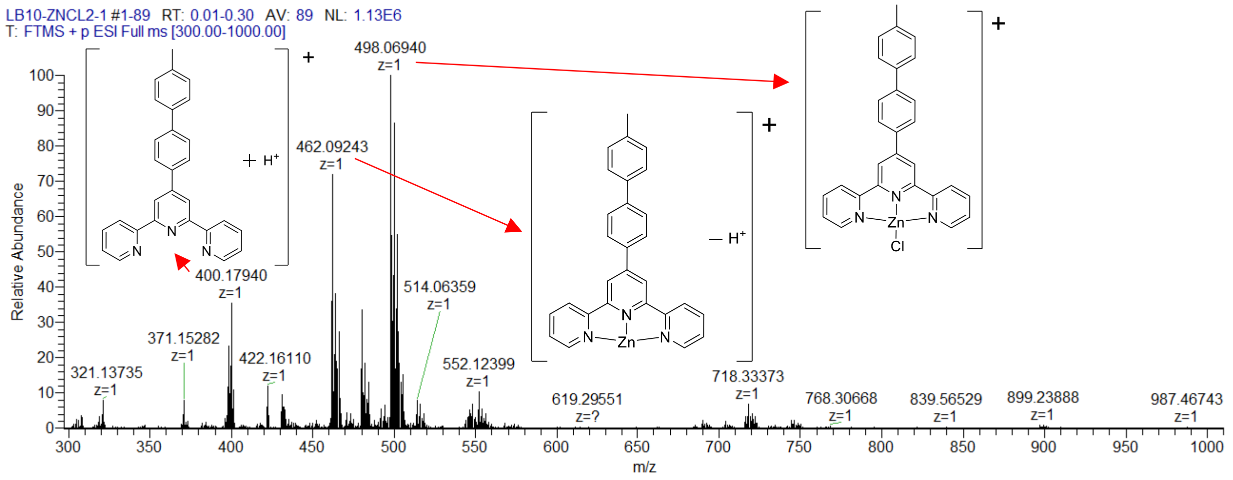

Figure S10 The ESI–MS spectrum of compound **5**.

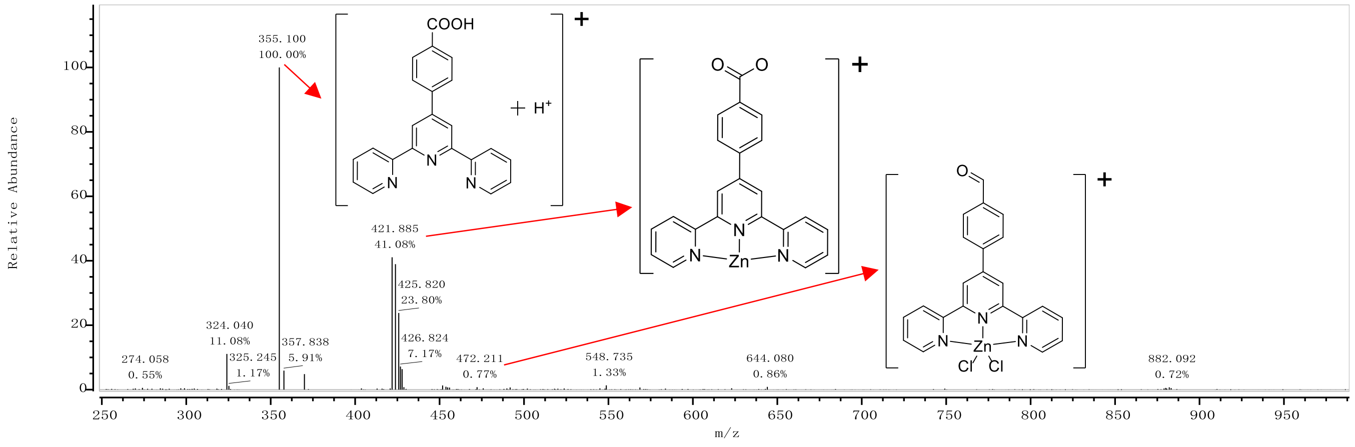

Figure S11 The ESI–MS spectrum of compound **9**.

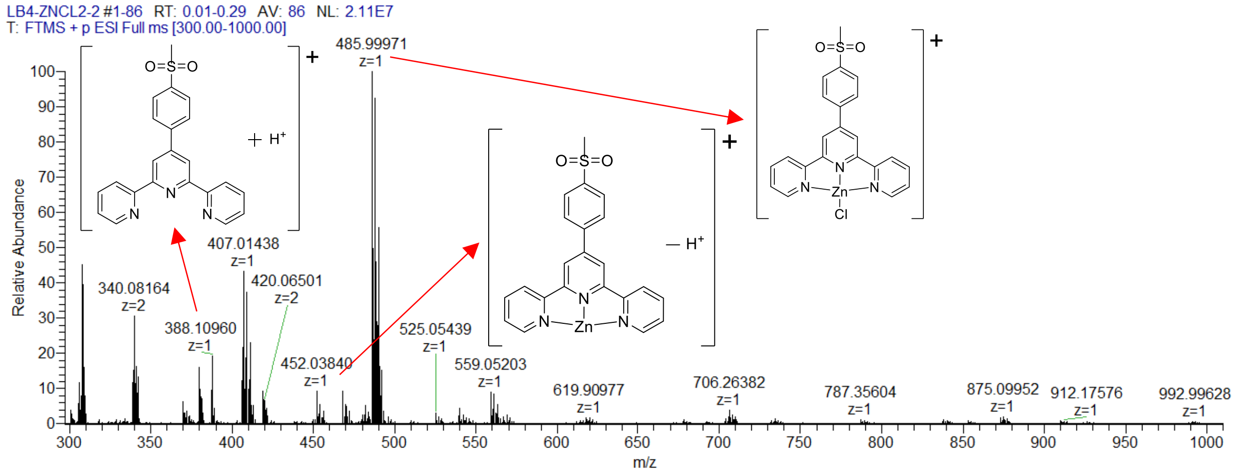

Figure S12 The ESI–MS spectrum of compound **10**.

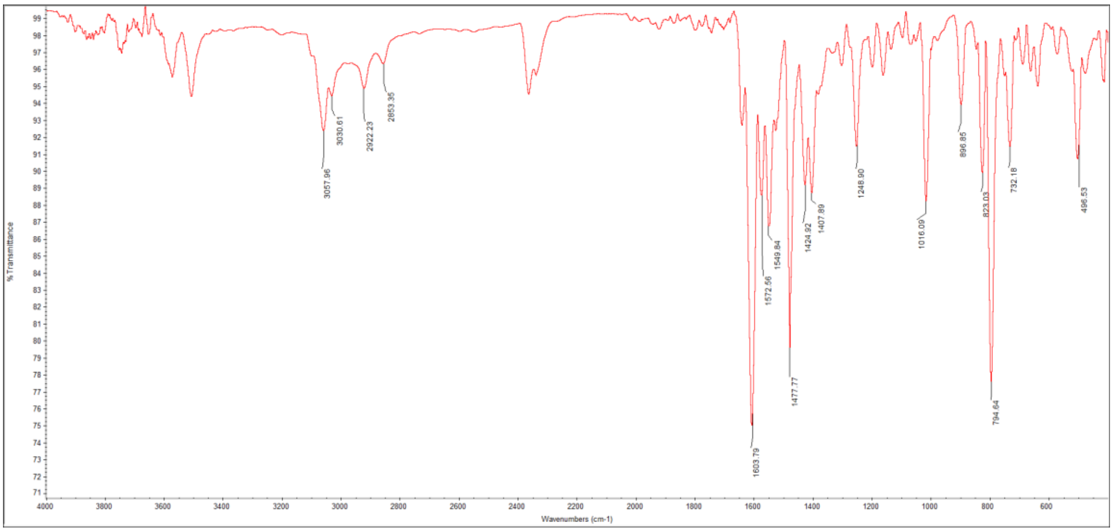

Figure S13 The IR spectrum of compound **2**.

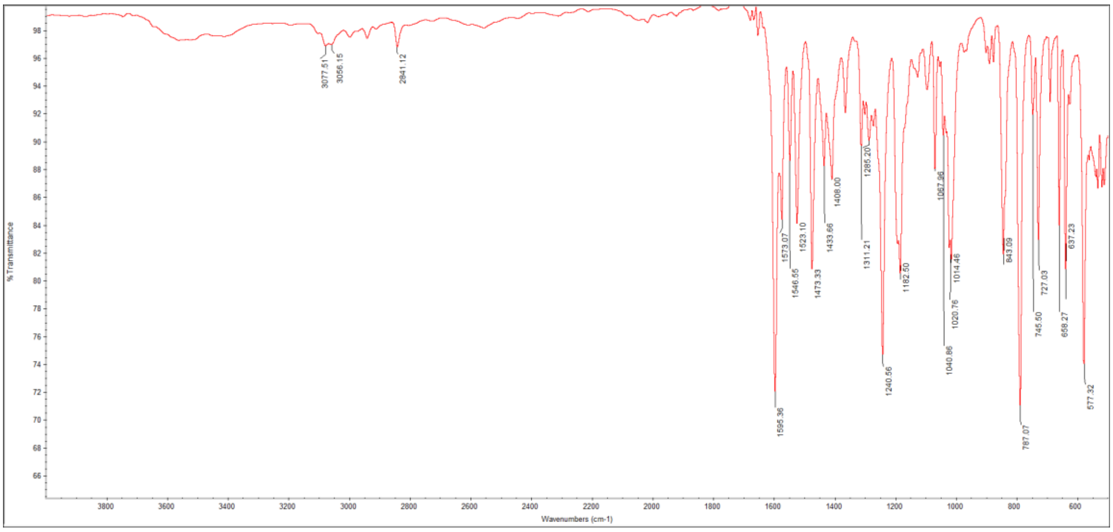

Figure S14 The IR spectrum of compound **3**.

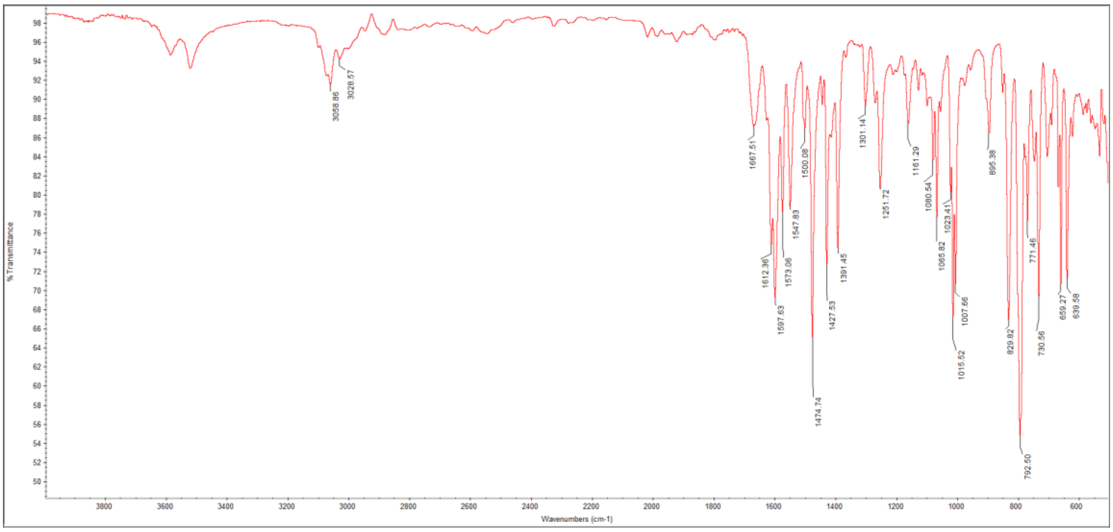

Figure S15 The IR spectrum of compound **4**.

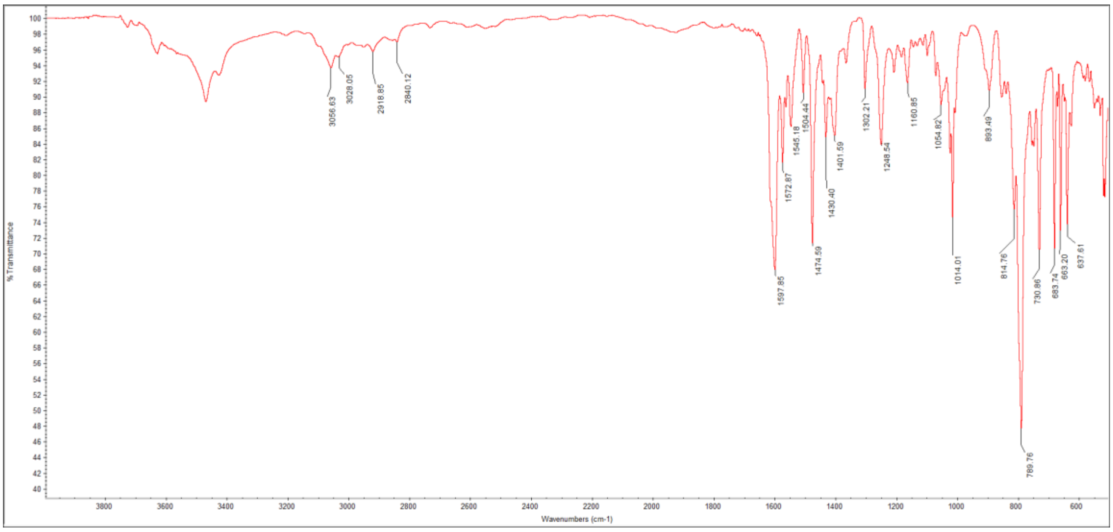

Figure S16 The IR spectrum of compound **5**.

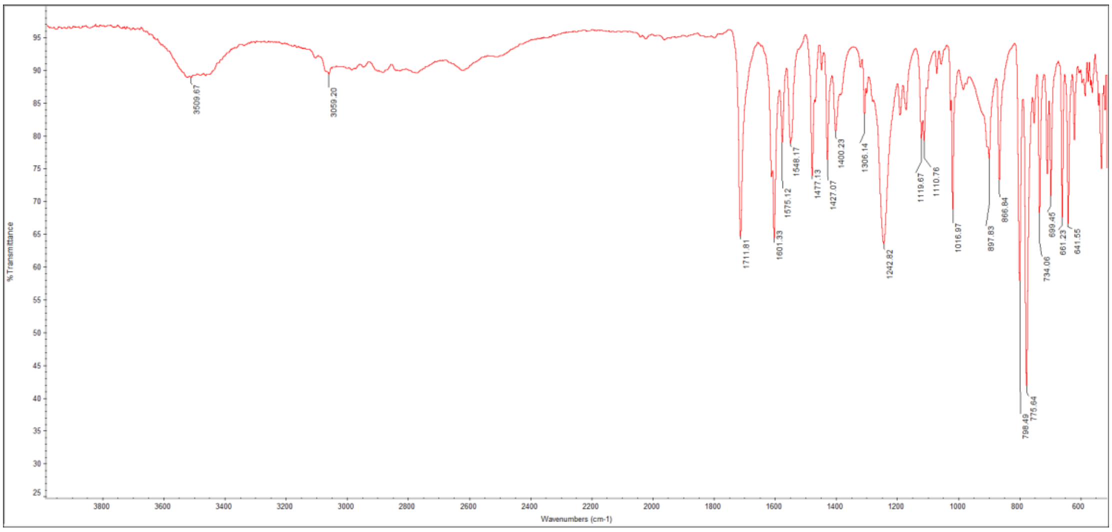

Figure S17 The IR spectrum of compound **9**.

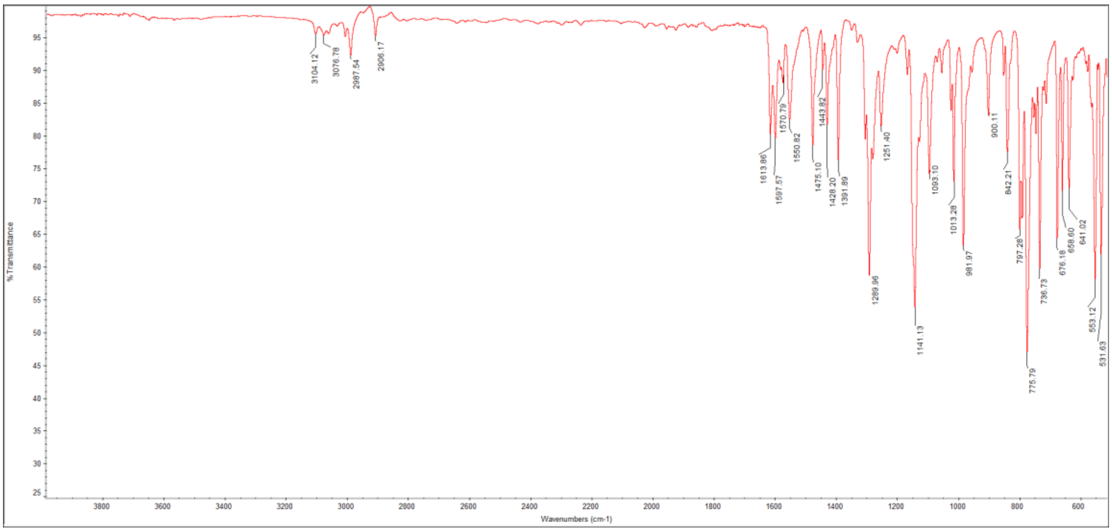

Figure S18 The IR spectrum of compound **10**.

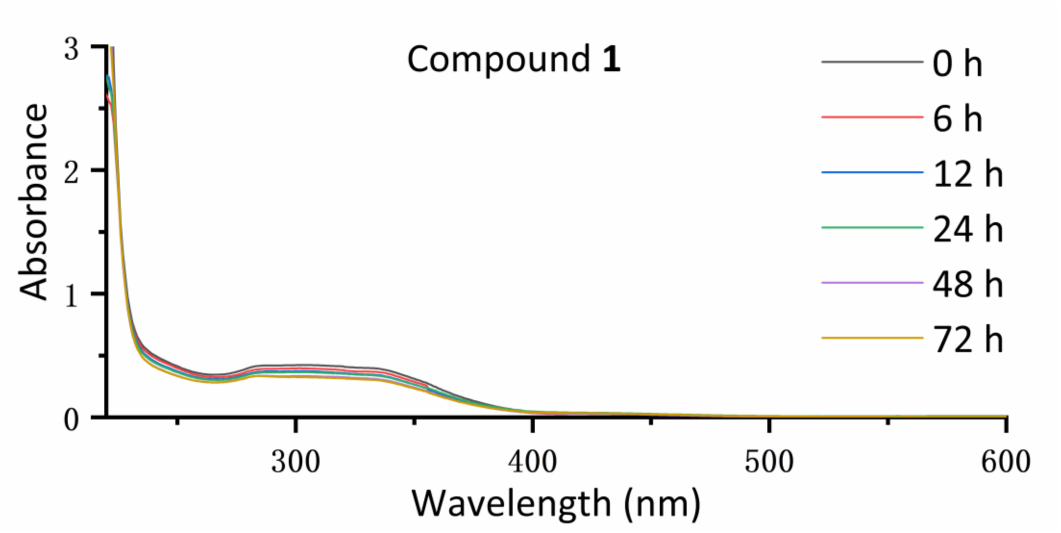

Figure S19 UV−vis spectra of compounds **1** for a period of 72 h.

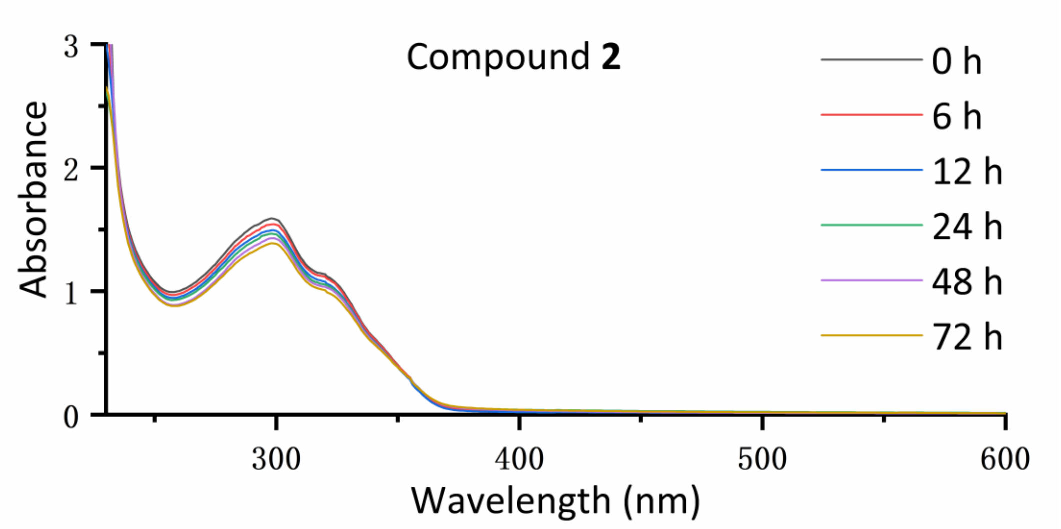

Figure S20 UV−vis spectra of compounds **2** for a period of 72 h.

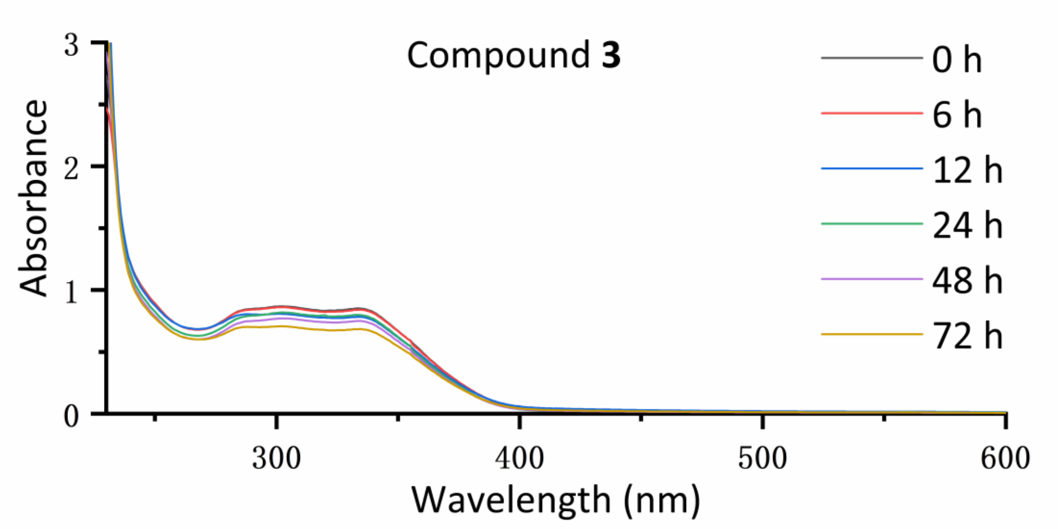

Figure S21 UV−vis spectra of compounds **3** for a period of 72 h.

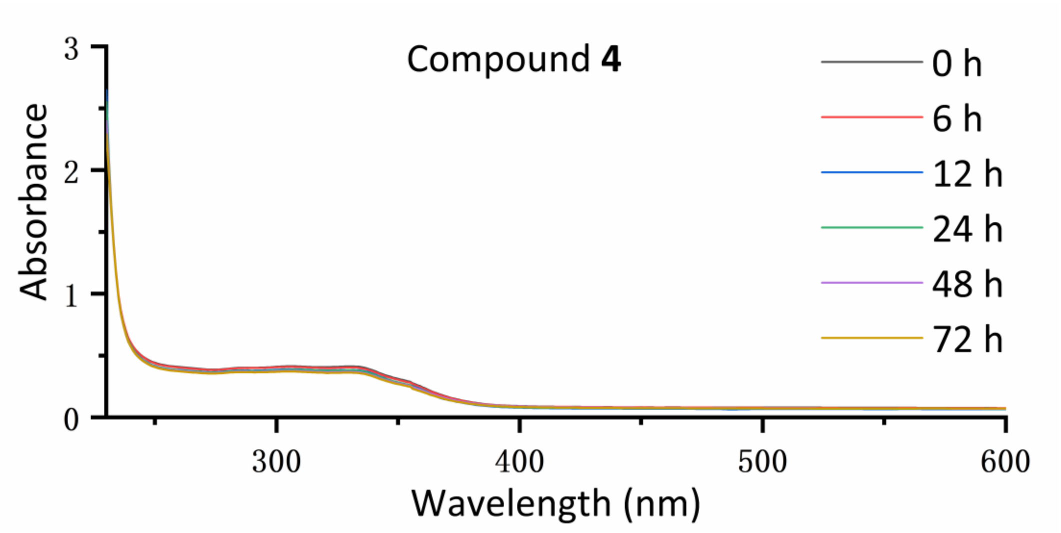

Figure S22 UV−vis spectra of compounds **4** for a period of 72 h.

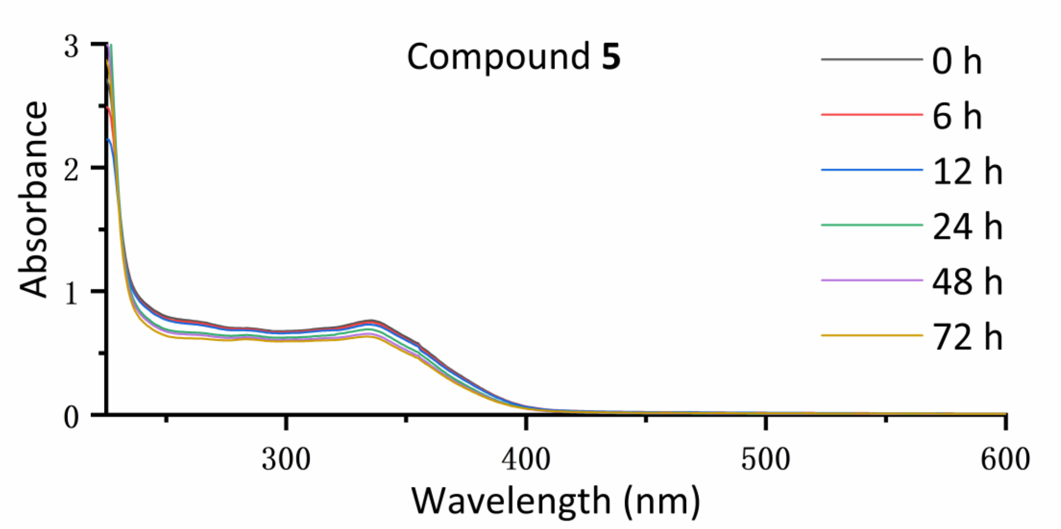

Figure S23 UV−vis spectra of compounds **5** for a period of 72 h.

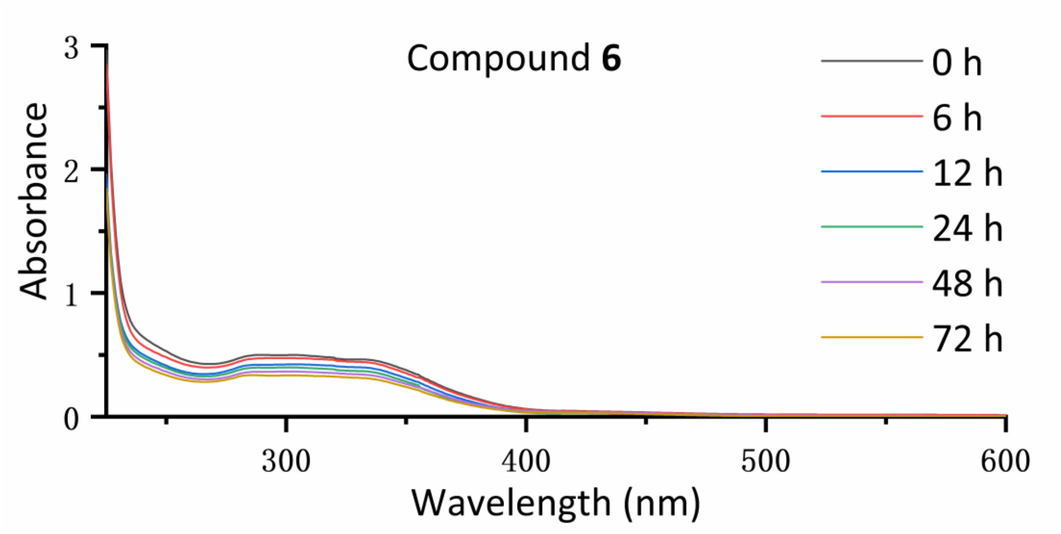

Figure S24 UV−vis spectra of compounds **6** for a period of 72 h.

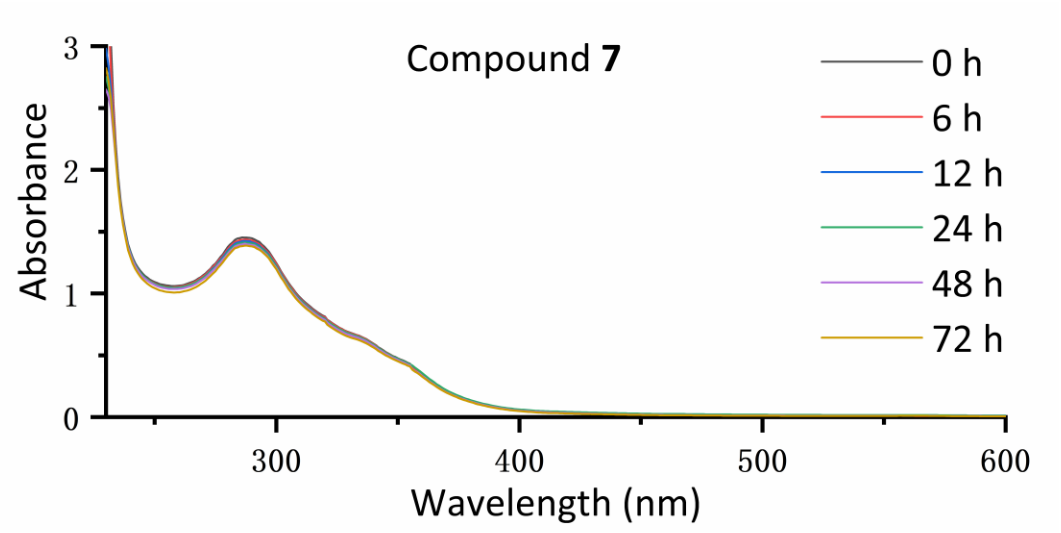

Figure S25 UV−vis spectra of compounds **7** for a period of 72 h.

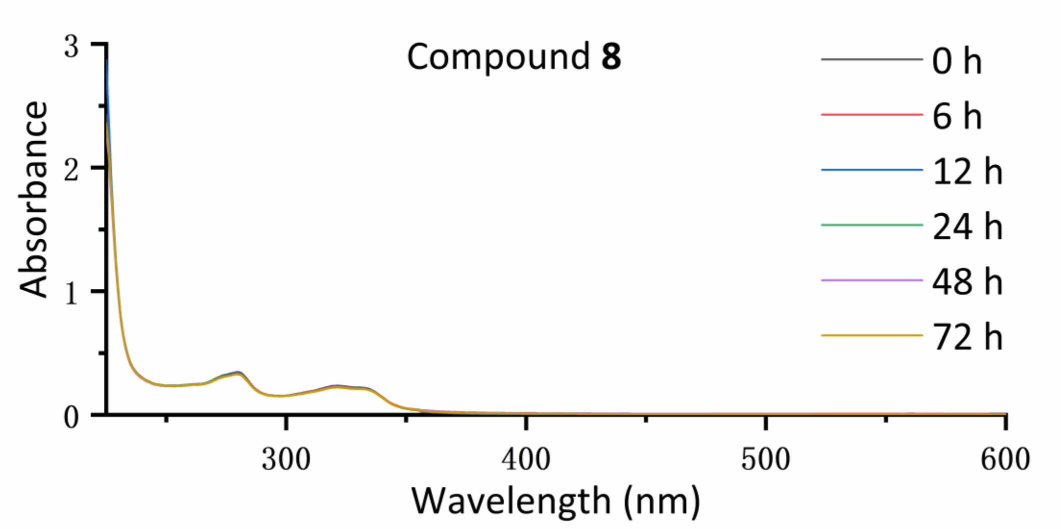

Figure S26 UV−vis spectra of compounds **8** for a period of 72 h.

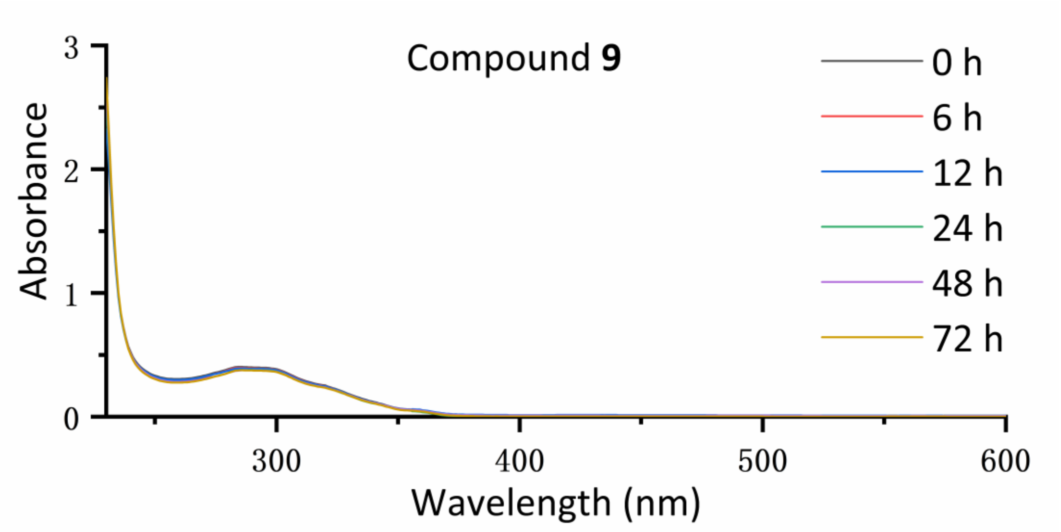

Figure S27 UV−vis spectra of compounds **9** for a period of 72 h.

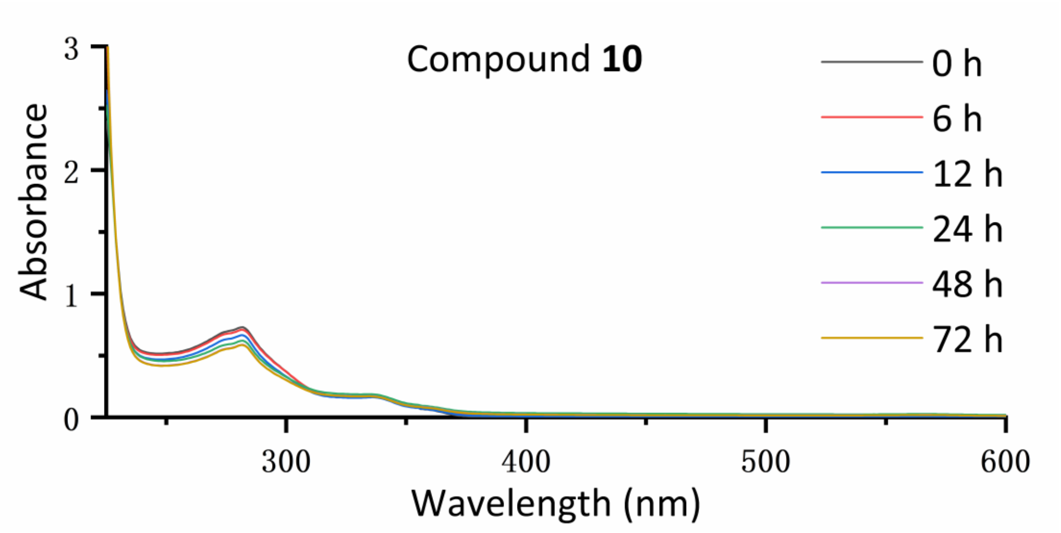

Figure S28 UV−vis spectra of compounds **10** for a period of 72 h.

Compound | Control | Compound
Low concentration → high concentration

1
2
3
4
5

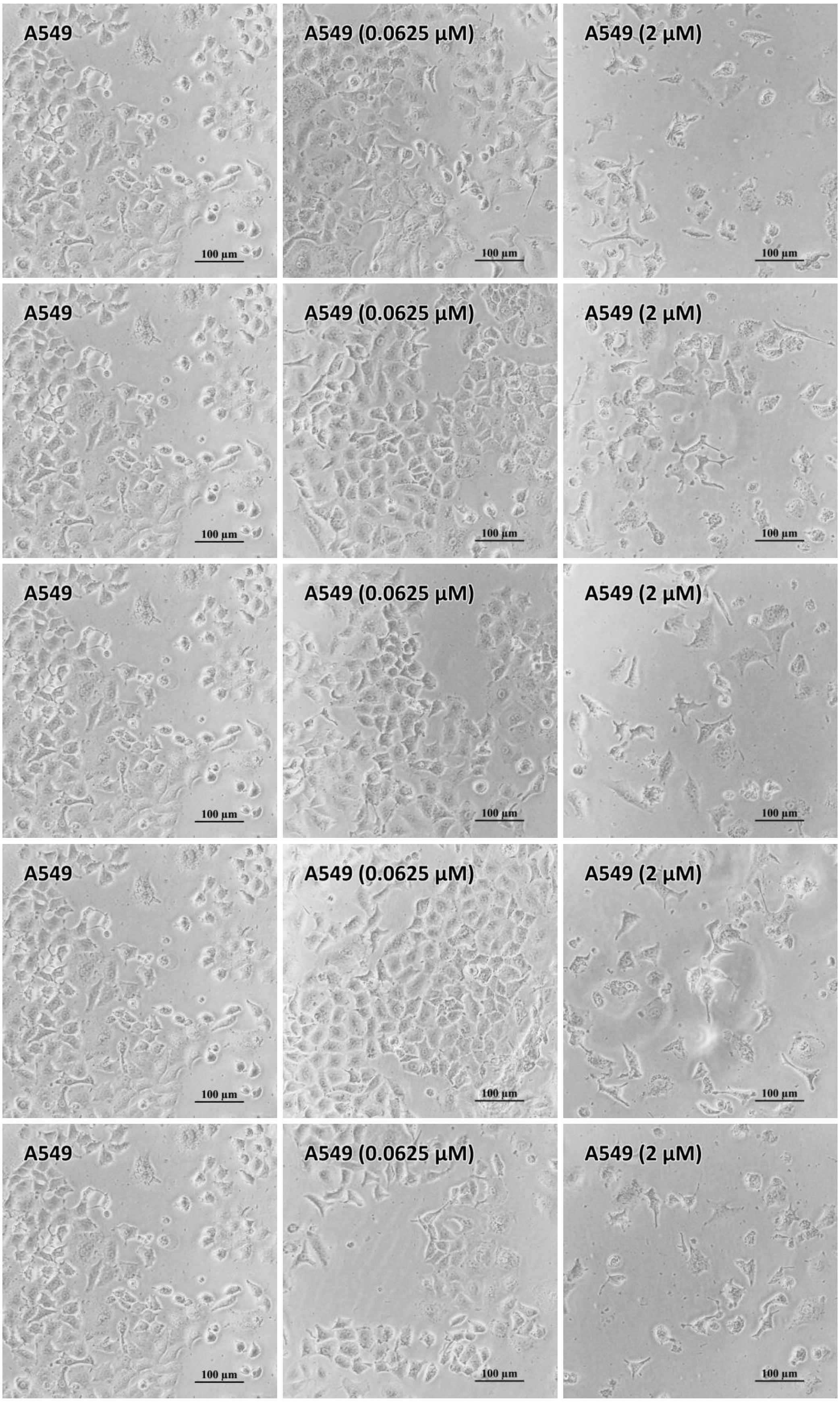

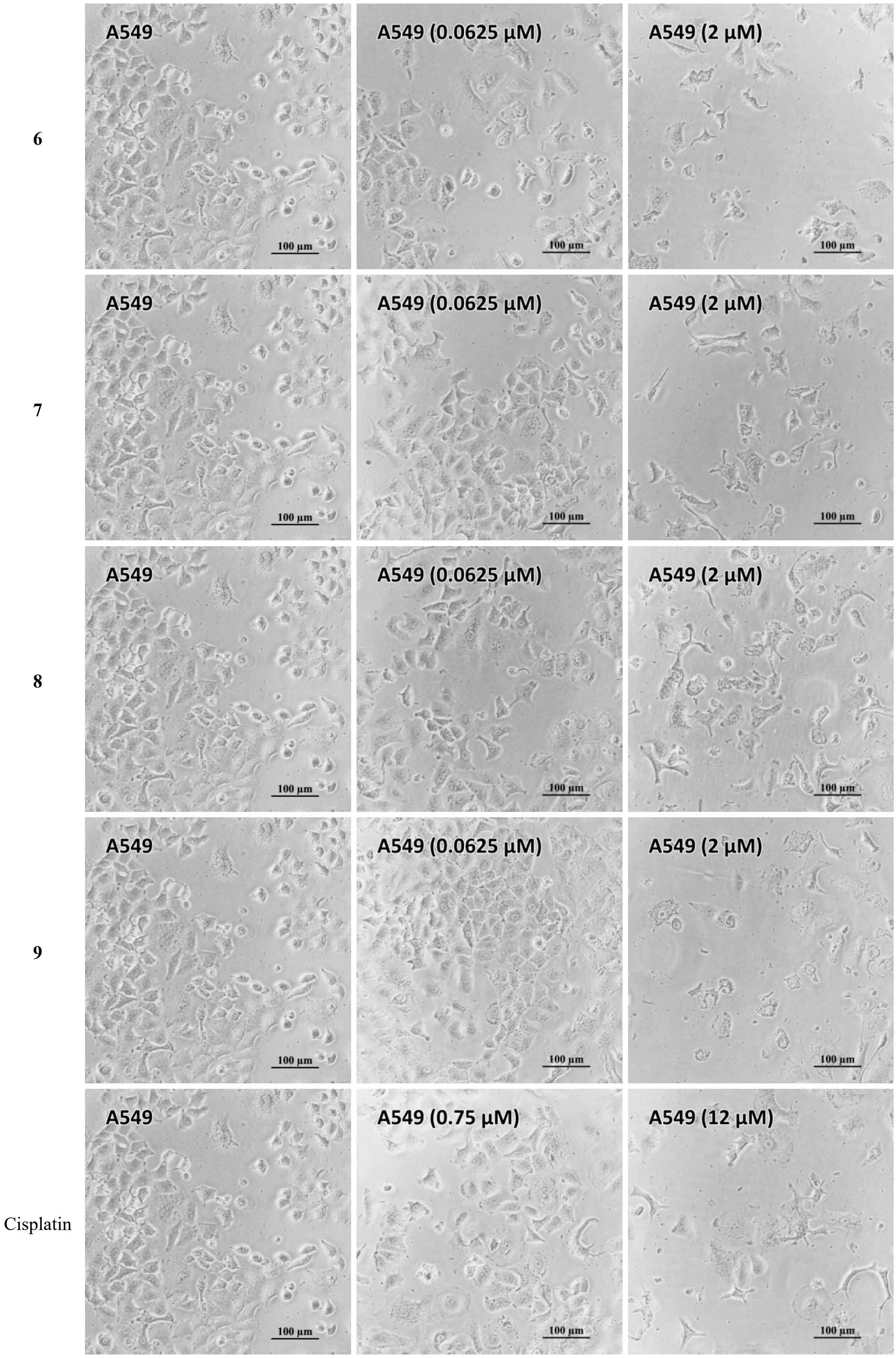

Figure S29 The microscopic photographs of the A549 cancer cell treated with increased concentrations of the compounds **1**–**9** and cisplatin at magnification of 200×.

Compound | Control | Compound
Low concentration → high concentration

1
2
3
4
5
6

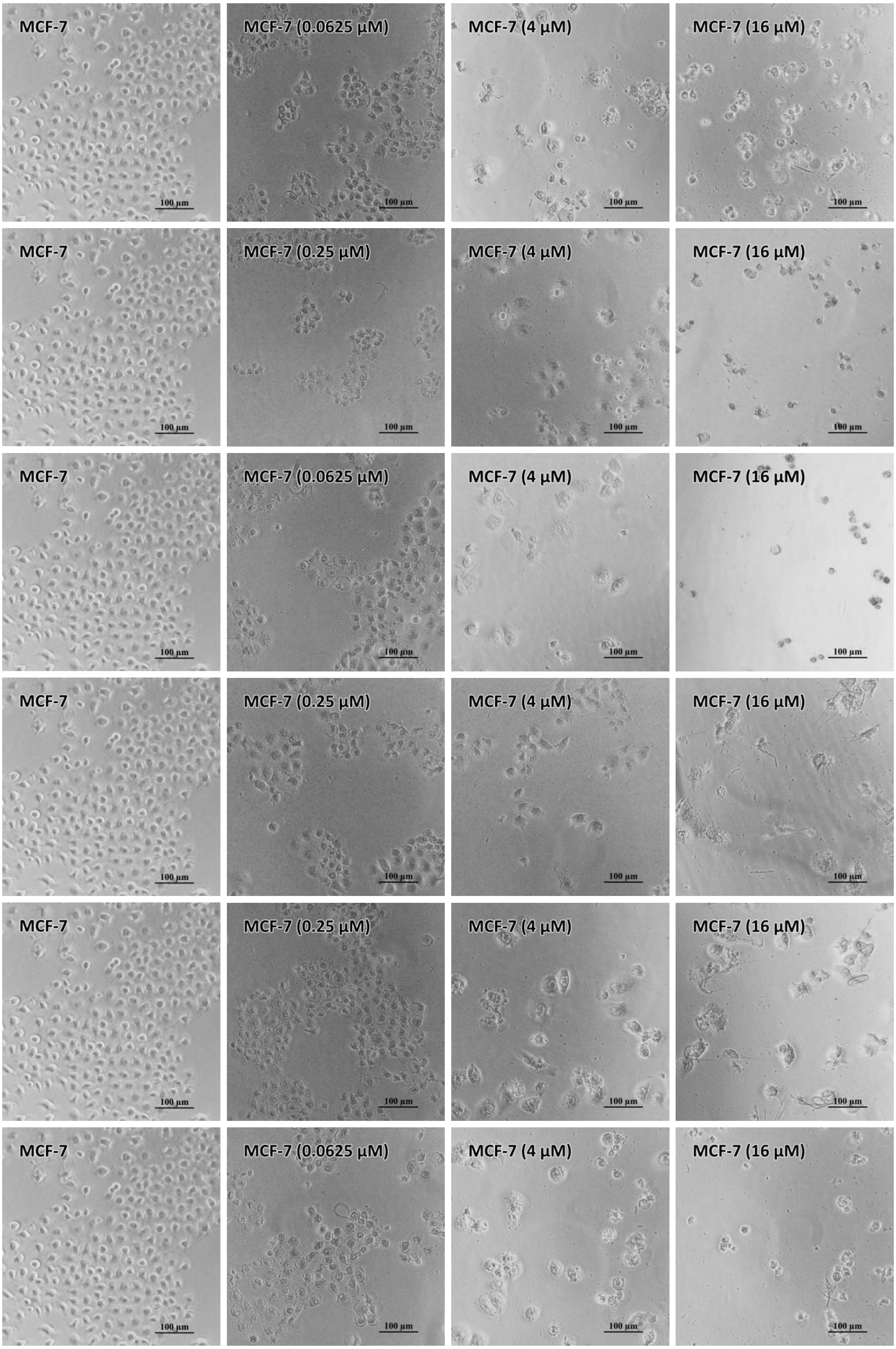

7

8

9

Cisplatin

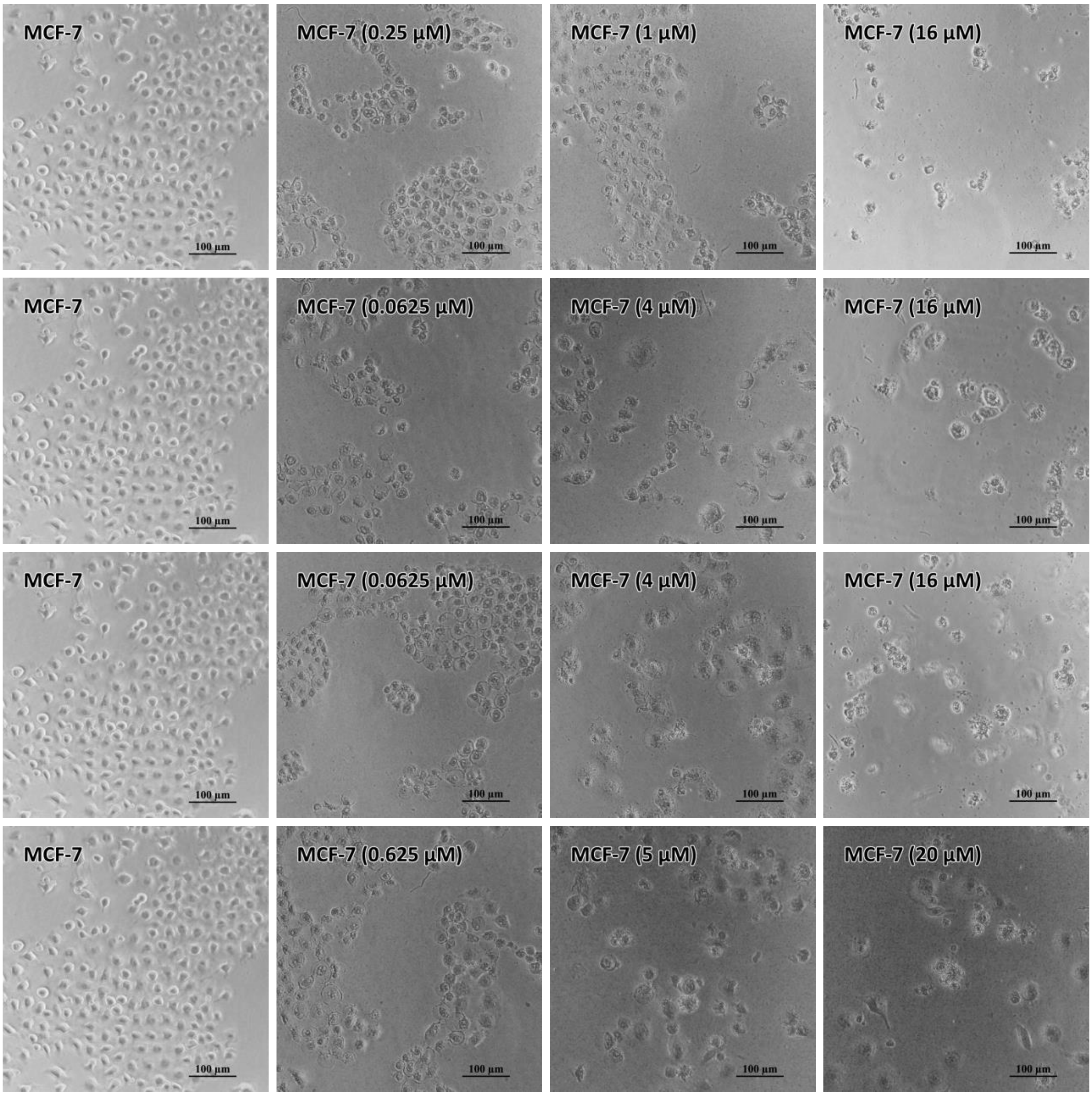

Figure S30 The microscopic photographs of the MCF-7 cancer cell treated with increased concentrations of the compounds **1**–**9** and cisplatin at magnification of 200×.

Compound | Control | Compound
Low concentration → high concentration

1

2

3

4

5

6

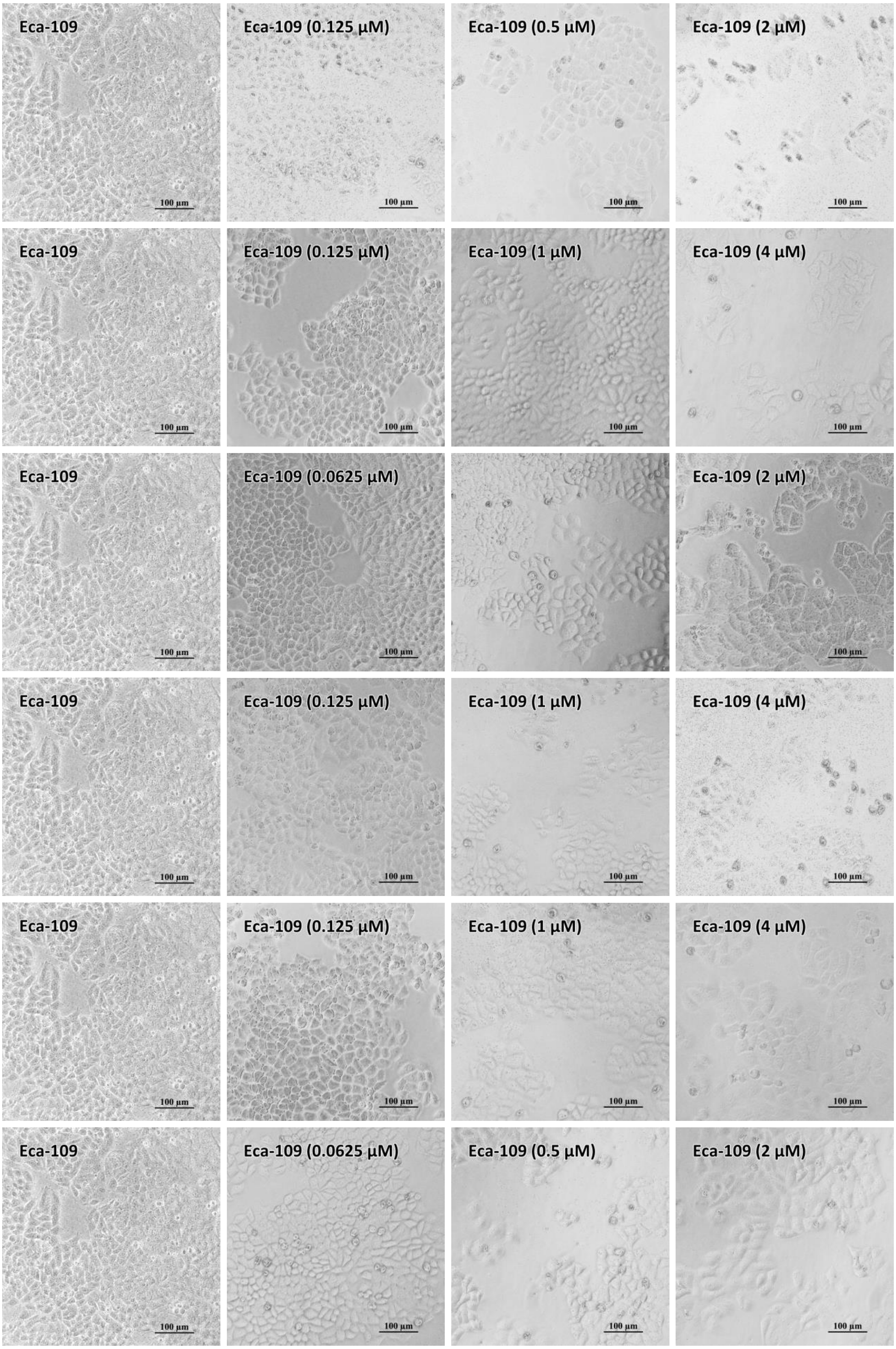

7
8
9
Cisplatin

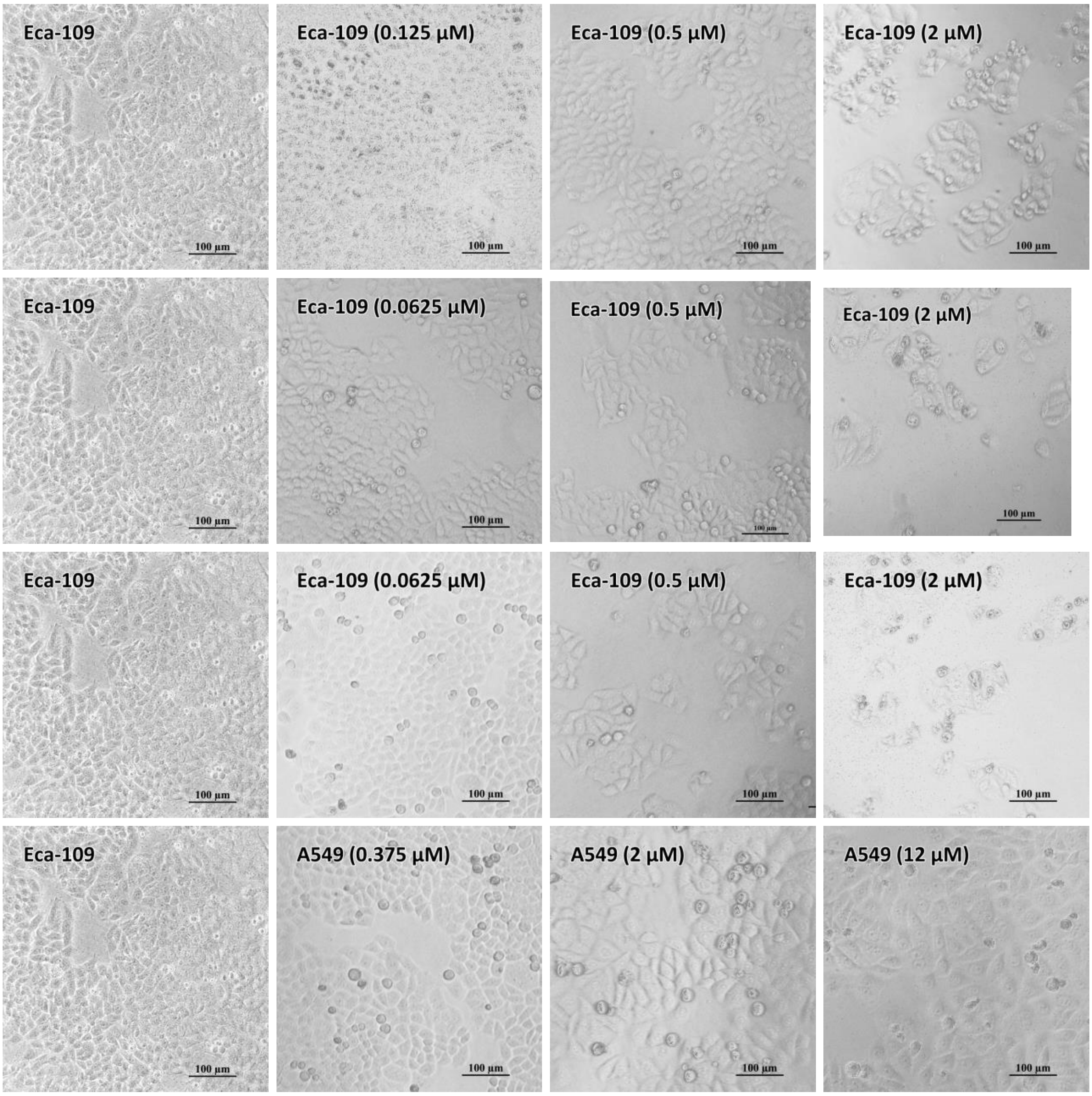

Figure S31 The microscopic photographs of the Eca-109 cancer cell treated with increased concentrations of the compounds **1**–**9** and cisplatin at magnification of 200×.

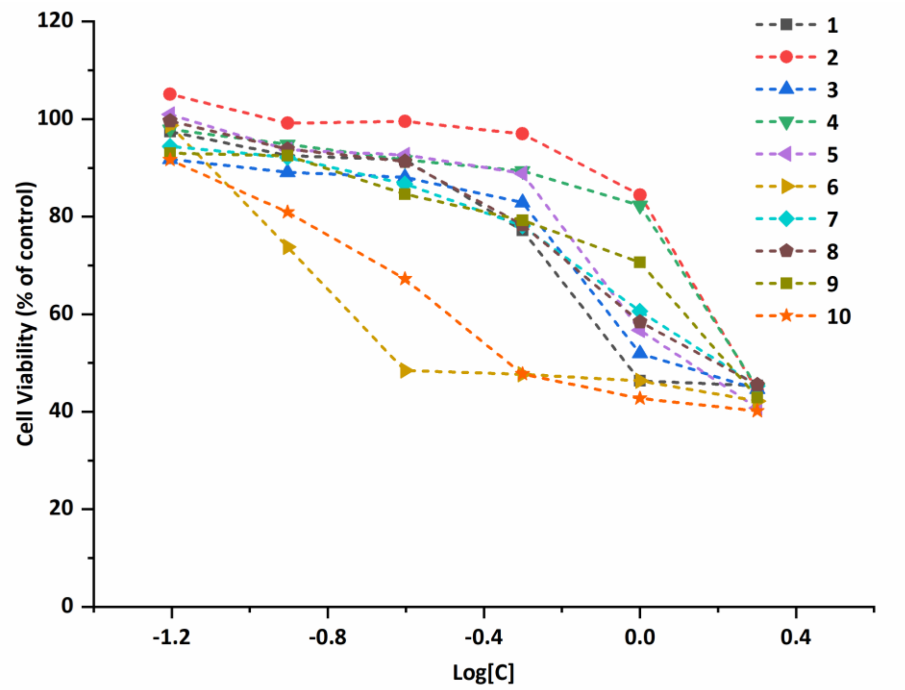

Figure S32 The plots of the cell viability *vs.* the concentration of compounds **1**–**10** against Bel-7402 cell line.

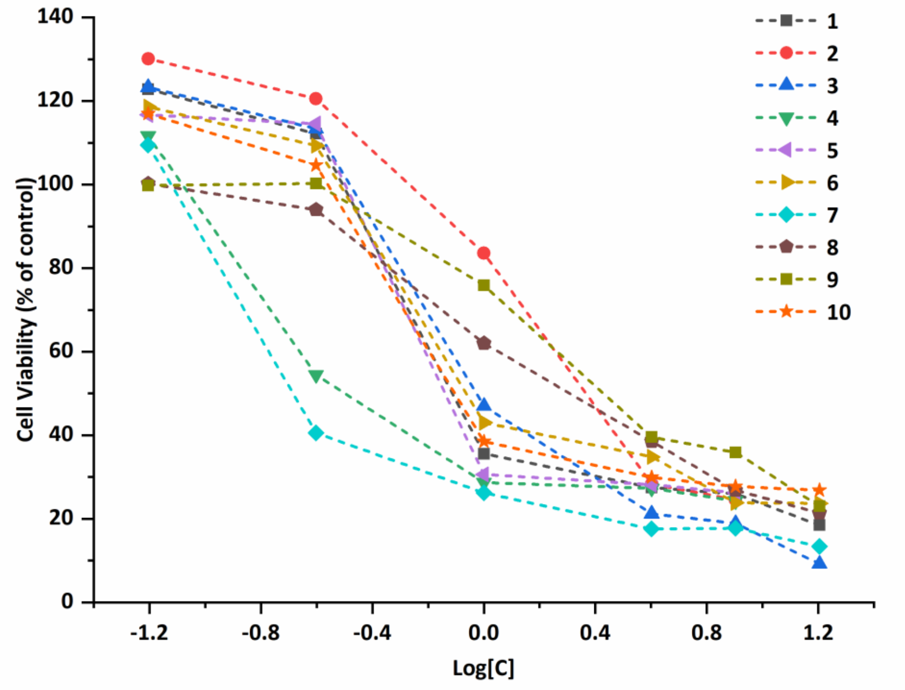

Figure S33 The plots of the cell viability *vs.* the concentration of compounds **1**–**10** against MCF-7 cell line.

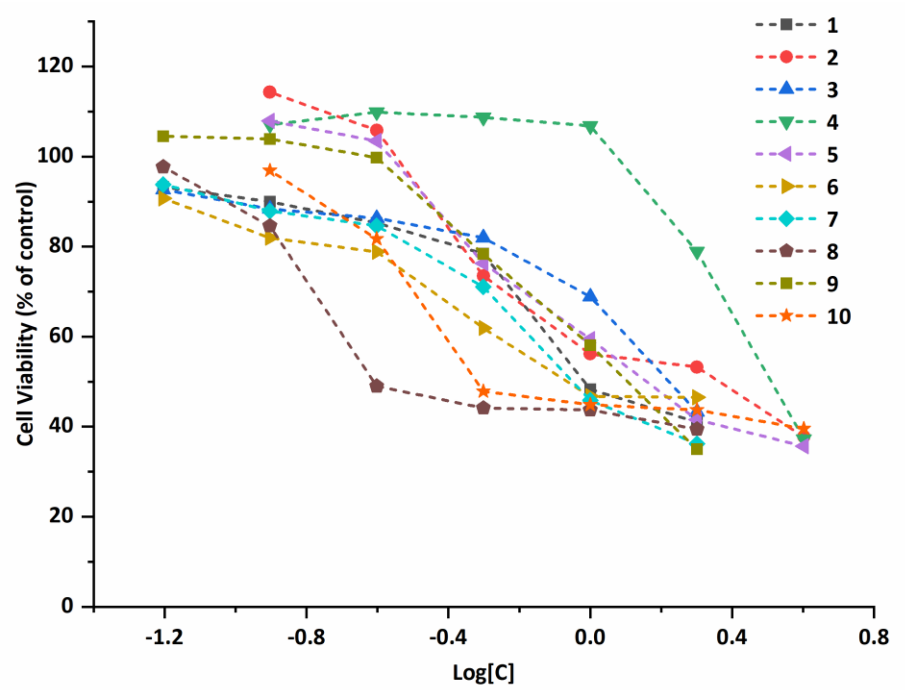

Figure S34 The plots of the cell viability *vs.* the concentration of compounds **1**–**10** against Eca-109 cell line.

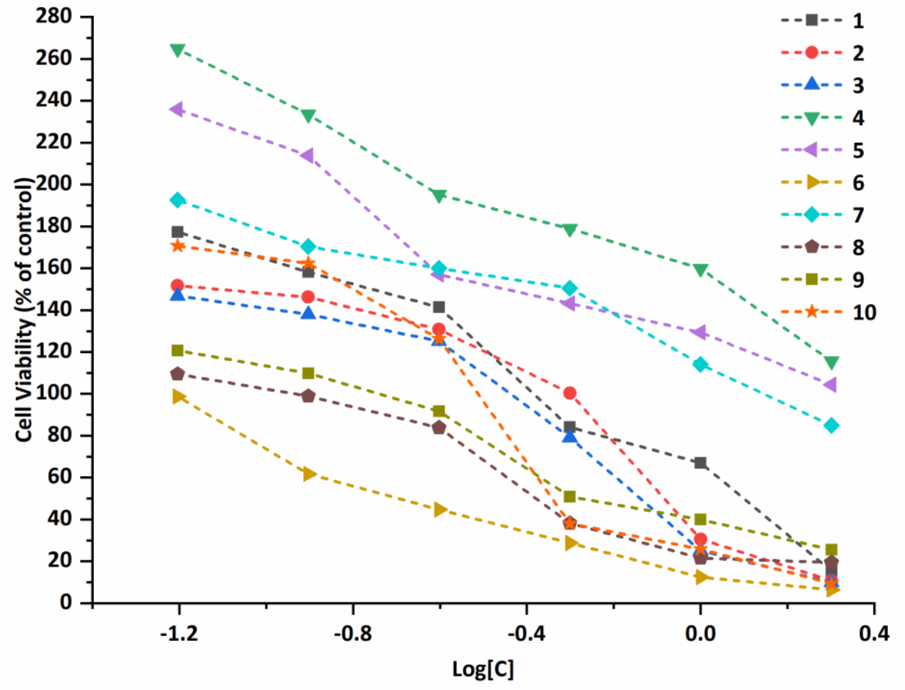

Figure S35 The plots of the cell viability *vs.* the concentration of compounds **1**–**10** against RAW 264.7 cell line.

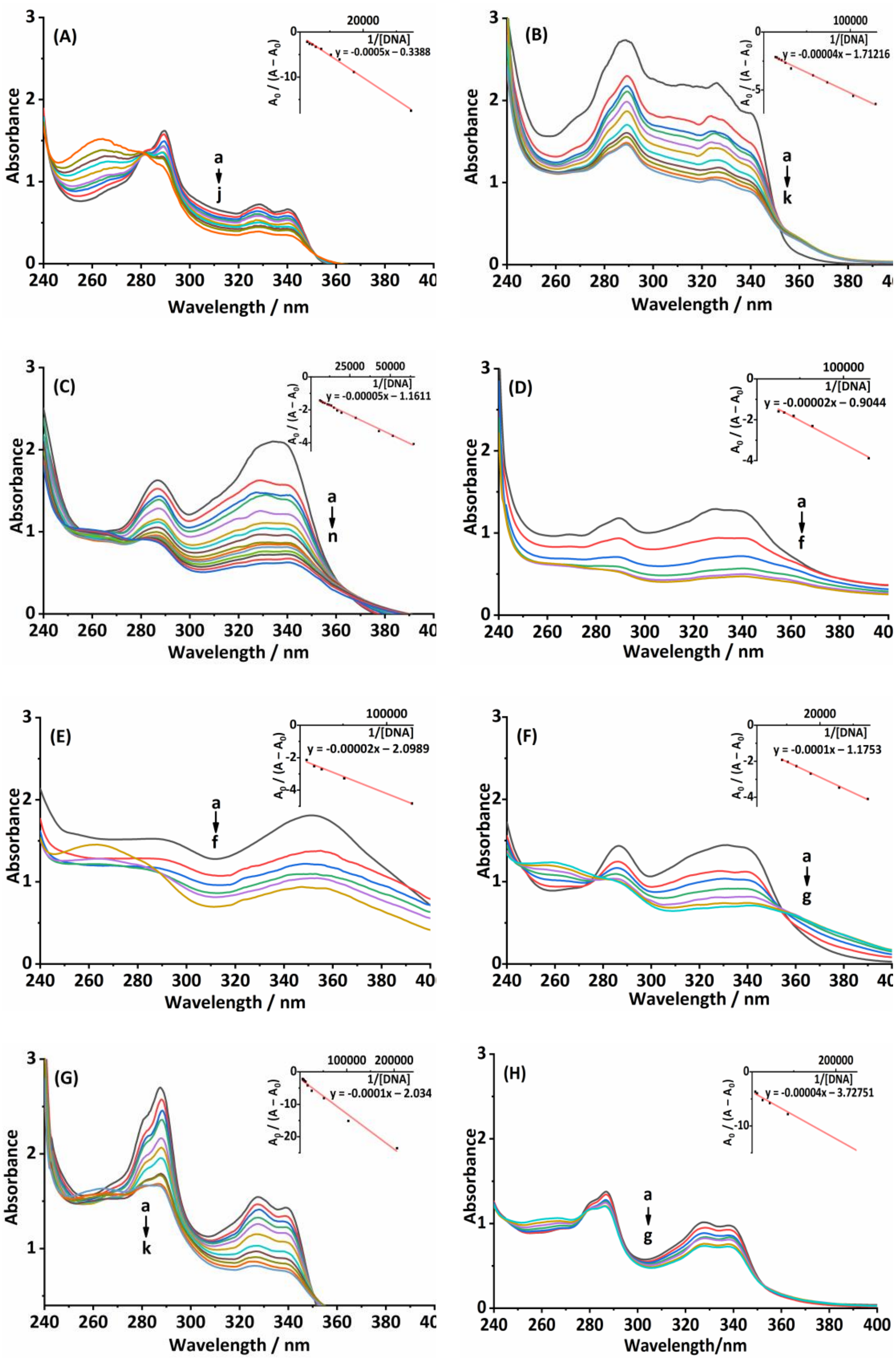
(A)
y = -0.0005x – 0.3388
(B)
y = -0.00004x – 1.71216
(C)
y = -0.00005x – 1.1611
(D)
y = -0.00002x – 0.9044
(E)
y = -0.00002x – 2.0989
(F)
y = -0.0001x – 1.1753
(G)
y = -0.0001x – 2.034
(H)
y = -0.00004x – 3.72751
1/[DNA]
A0 / (A – A0)
Absorbance
Wavelength / nm

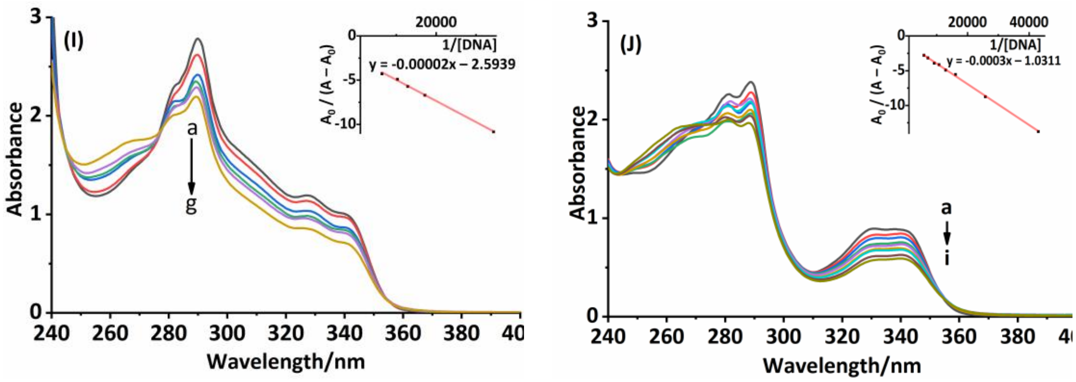

Figure S36 UV–vis spectra for compounds **1**–**10** in Tris-HCl 1.0 × 10^{-3} (pH 7.2) with increasing the concentration of CT-DNA, and the inset show plots of $A_0/(A–A_0)$ versus the concentration of CT-DNA. (A) c(**1**) = 67 μM; c(CT-DNA) = 0, 30, 60, 80, 100, 140, 180, 220 , 260 and 320 μM corresponding to the curves from a to j, respectively. (B) c(**2**) = 100 μM; c(CT-DNA) = 0, 8, 10, 14, 18, 32, 40, 48, 56, 64, and 72 μM corresponding to the curves from a to k, respectively. (C) c(**3**) = 67 μM; c(CT-DNA) = 0, 16, 20, 36, 52, 60, 68, 76, 84, 92, 108, 124, 140, and 156 μM corresponding to the curves from a to n, respectively. (D) c(**4**) = 100 μM; c(CT-DNA) = 0, 8, 16, 24, 32, and 40 μM corresponding to the curves from a to f, respectively. (E) c(**5**) = 100 μM; c(CT-DNA) = 0, 8, 20, 40, 60, and 120 μM corresponding to the curves from a to f, respectively. (F) c(**6**) = 100 μM; c(CT-DNA) = 0, 30, 40, 60, 80, 100 and 120 μM corresponding to the curves from a to g, respectively. (G) c(**7**) = 100 μM; c(CT-DNA) = 0, 5, 10, 20, 40, 60, 80, 100, 120, 140, and 160 μM corresponding to the curves from a to k, respectively. (H) c(**8**) = 100 μM; c(CT-DNA) = 0, 4, 12, 24, 40, 80 and 120 μM corresponding to the curves from a to g, respectively. (I) c(**9**) = 100 μM; c(CT-DNA) = 0, 30, 60, 80, 100 and 160 μM corresponding to the curves from a to f, respectively. (J) c(**10**) = 80 μM; c(CT-DNA) = 0, 25, 40, 60, 75, 90, 110, 140 and 170 μM corresponding to the curves from a to i, respectively.

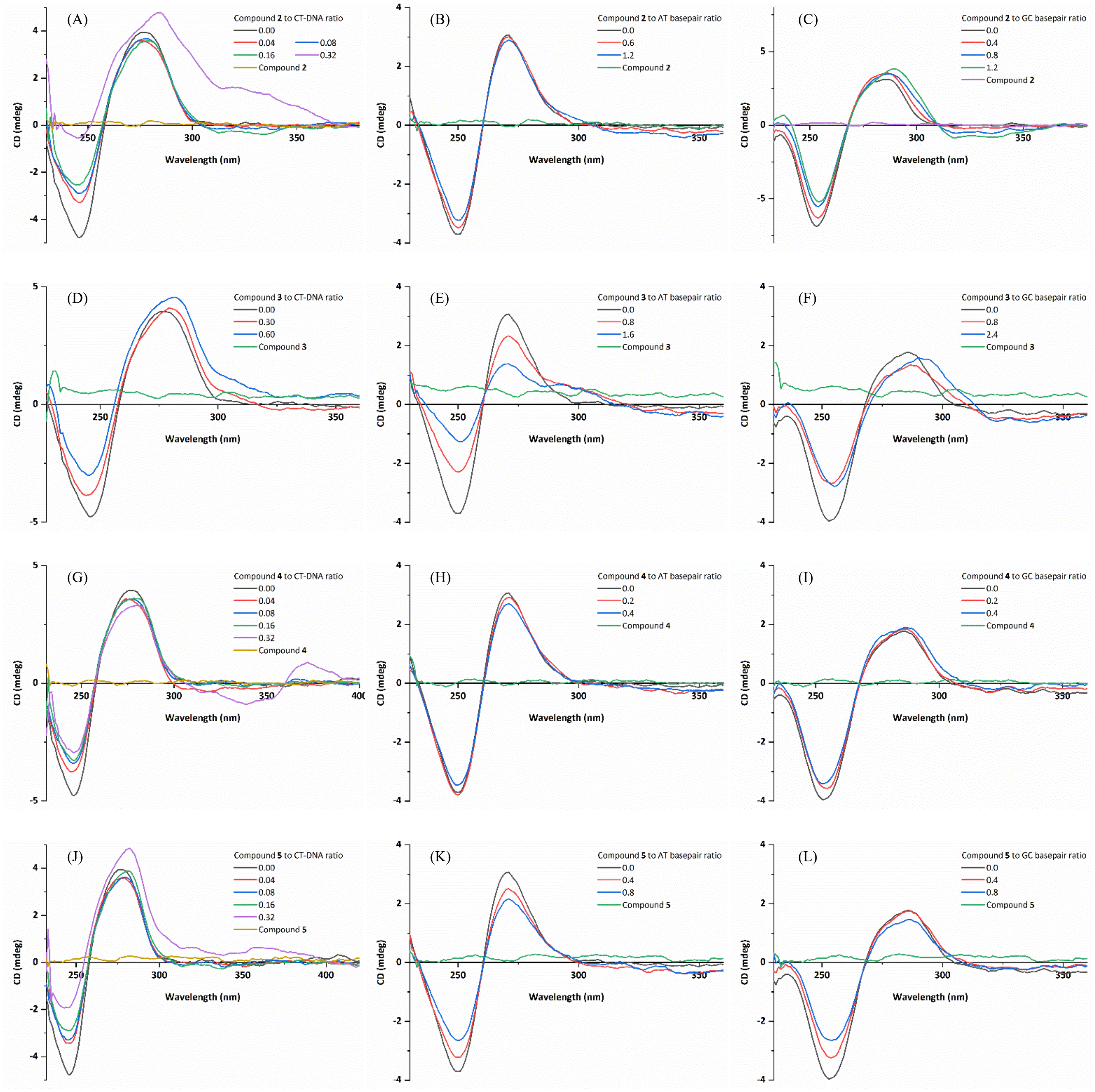

Figure S37 Circular dichroism spectra of three kinds of DNA in the presence or absence of compounds **2**–**5** in Tris-HCl buffer (pH 7.2), at 20 °C. (A) CT-DNA (4.0×10^{-4} M) treated with compound **2**, (B) ds(AT)$_6$ (1.44×10^{-5} M) treated with compound **2**, (C) ds(GC)$_6$ (1.58×10^{-5} M) treated with compound **2** , (D) CT-DNA (4.0×10^{-4} M) treated with compound **3** , (E) ds(AT)$_6$ (1.44×10^{-5} M) treated with compound **3**, (F) ds(GC)$_6$ (8.7×10^{-6} M) treated with compound **3**, (G) CT-DNA (4.0×10^{-4} M) treated with compound **4**, (H) ds(AT)$_6$ (1.44×10^{-5} M) treated with compound **4**, (I) ds(GC)$_6$ (8.7×10^{-6}) treated with compound **4**, (J) CT-DNA (4.0×10^{-4} M) treated with compound **5**, (K) ds(AT)$_6$ (1.44×10^{-5} M) treated with compound **5**, (L) ds(GC)$_6$ (8.7×10^{-6}) treated with compound **5**.

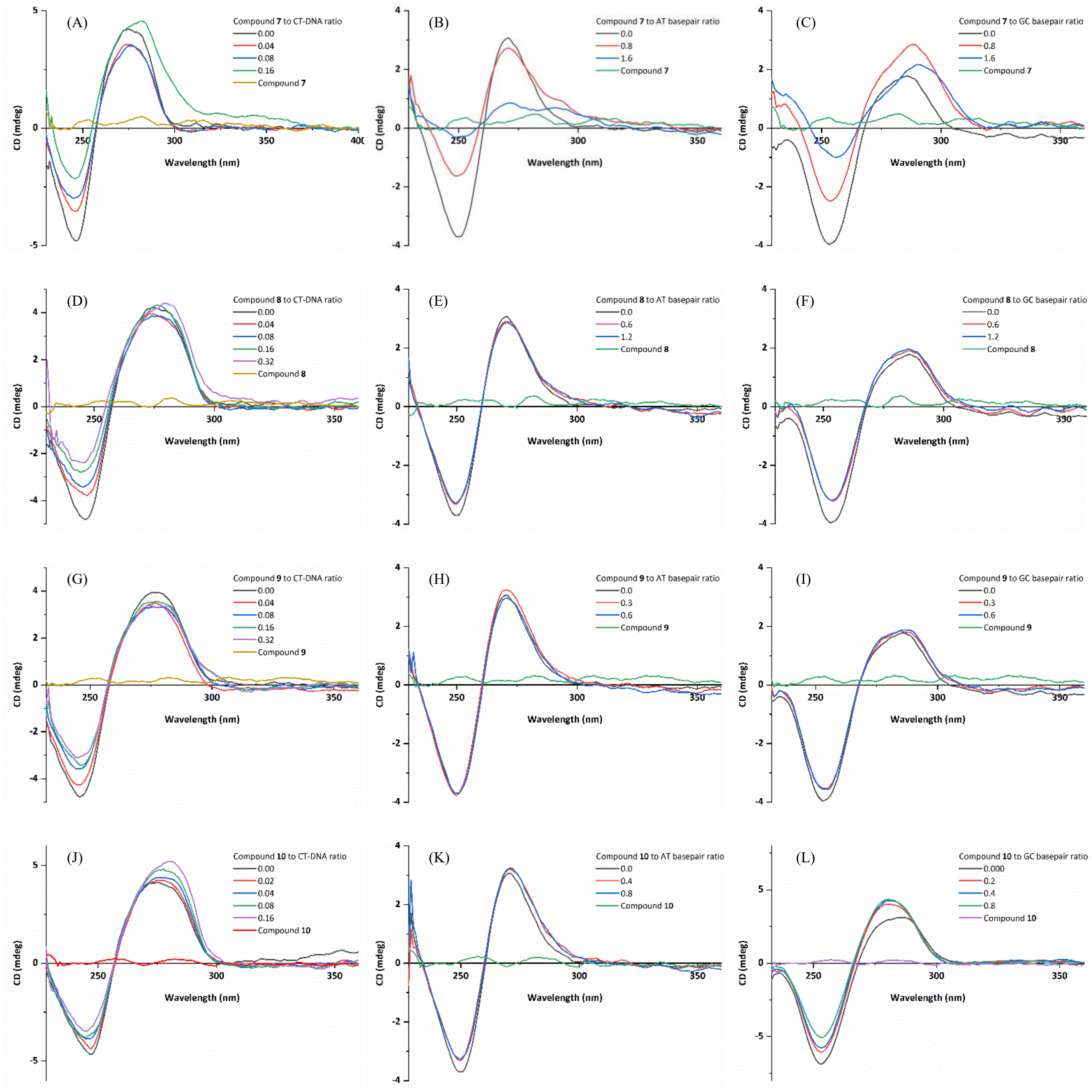

Figure S38 Circular dichroism spectra of three kinds of DNA in the presence or absence of compounds **7**–**10** in Tris-HCl buffer (pH 7.2), at 20 °C. (A) CT-DNA (4.0×10^{-4} M) treated with compound **7**, (B) ds(AT)$_6$ (1.44×10^{-5} M) treated with compound **7**, (C) ds(GC)$_6$ (1.58×10^{-5} M) treated with compound **7** , (D) CT-DNA (4.0×10^{-4} M) treated with compound **8** , (E) ds(AT)$_6$ (1.44×10^{-5} M) treated with compound **8**, (F) ds(GC)$_6$ (8.7×10^{-6} M) treated with compound **8**, (G) CT-DNA (4.0×10^{-4} M) treated with compound **9**, (H) ds(AT)$_6$ (1.44×10^{-5} M) treated with compound **9**, (I) ds(GC)$_6$ (8.7×10^{-6}) treated with compound **9**, (J) CT-DNA (4.0×10^{-4} M) treated with compound **10**, (K) ds(AT)$_6$ (1.44×10^{-5} M) treated with compound **10**, (L) ds(GC)$_6$ (1.58×10^{-5} M) treated with compound **10**.

Table S1 CD sprctra bands of CT-DNA, ds(AT)$_6$, ds(GC)$_6$ and with compounds **1**–**10**: wavelength λ (degree of ellipcity, Φ)

	λ/nm (Φ/°)	
	(–) band	(+) band
400 µM CT-DNA	246(-4.77)	277(3.94)
400 µM CT-DNA + 128 µM compound **1**	245(-3.79)	279(4.69)
400 µM CT-DNA + 256 µM compound **1**	245(-3.04)	279(4.73)
400 µM CT-DNA + 512 µM compound **1**	244(-2.53)	282(6.45)
400 µM CT-DNA + 16 µM compound **2**	246(-3.30)	276(3.57)
400 µM CT-DNA + 32 µM compound **2**	246(-2.89)	277(3.67)
400 µM CT-DNA + 64 µM compound **2**	245(-2.54)	278(3.59)
400 µM CT-DNA + 128 µM compound **2**	245(-0.55)	285(4.76)
400 µM CT-DNA + 160 µM compound **3**	245(-3.85)	280(4.07)
400 µM CT-DNA + 320 µM compound **3**	245(-3.02)	282(4.55)
400 µM CT-DNA + 16 µM compound **4**	245(-3.76)	274(3.60)
400 µM CT-DNA + 32 µM compound **4**	246(-3.40)	277(3.56)
400 µM CT-DNA + 64 µM compound **4**	246(-3.29)	281(3.59)
400 µM CT-DNA + 128 µM compound **4**	246(-2.94)	281(3.30)
400 µM CT-DNA + 16 µM compound **5**	246(-3.42)	278(3.59)
400 µM CT-DNA + 32 µM compound **5**	245(-3.29)	280(3.61)
400 µM CT-DNA + 64 µM compound **5**	245(-2.90)	282(3.88)
400 µM CT-DNA + 128 µM compound **5**	243(-1.93)	282(4.85)
400 µM CT-DNA + 8 µM compound **6**	246(-3.54)	277(3.60)
400 µM CT-DNA + 16 µM compound **6**	246(-3.62)	277(3.39)
400 µM CT-DNA + 32 µM compound **6**	246(-3.19)	277(3.42)
400 µM CT-DNA + 64 µM compound **6**	246(-2.34)	279(3.64)
400 µM CT-DNA + 128 µM compound **6**	246(-1.28)	283(4.47)
400 µM CT-DNA + 256 µM compound **6**	246(0.66)	288(5.71)
400 µM CT-DNA + 8 µM compound **7**	246(-3.57)	275(3.54)
400 µM CT-DNA + 32 µM compound **7**	246(-2.96)	278(3.48)
400 µM CT-DNA + 64 µM compound **7**	246(-2.15)	281(4.55)
400 µM CT-DNA + 16 µM compound **8**	247(-3.79)	274(3.95)
400 µM CT-DNA + 32 µM compound **8**	246(-3.44)	276(3.85)
400 µM CT-DNA + 64 µM compound **8**	245(-2.79)	277(4.31)
400 µM CT-DNA + 128 µM compound **8**	245(-2.37)	279(4.40)
400 µM CT-DNA + 16 µM compound **9**	246(-4.27)	276(3.48)
400 µM CT-DNA + 32 µM compound **9**	246(-3.59)	277(3.32)
400 µM CT-DNA + 64 µM compound **9**	246(-3.43)	277(3.56)
400 µM CT-DNA + 128 µM compound **9**	245(-3.11)	280(3.35)
400 µM CT-DNA + 8 µM compound **10**	246(-4.35)	277(4.21)
400 µM CT-DNA + 16 µM compound **10**	246(-3.87)	278(4.35)
400 µM CT-DNA + 32 µM compound **10**	245(-3.80)	278(4.82)
400 µM CT-DNA + 64 µM compound **10**	245(-3.48)	280(5.20)
15.7 µM ds(AT)$_6$	250(-4.43)	271(3.59)
15.7 µM ds(AT)$_6$ + 160 µM compound **1**	249(-2.97)	271(3.00)
15.7 µM ds(AT)$_6$ + 320 µM compound **1**	251(-0.82)	275(1.40)
15.7 µM ds(AT)$_6$ + 480 µM compound **1**	251(-0.37)	275(1.09)
14.4 µM ds(AT)$_6$	250(-3.70)	271(3.07)
14.4 µM ds(AT)$_6$ + 108 µM compound **2**	250(-3.48)	271(3.00)
14.4 µM ds(AT)$_6$ + 180 µM compound **2**	250(-3.23)	271(2.90)
14.4 µM ds(AT)$_6$ + 144 µM compound **3**	250(-2.30)	271(2.33)
14.4 µM ds(AT)$_6$ + 288 µM compound **3**	251(-1.27)	271(1.38)
14.4 µM ds(AT)$_6$ + 36 µM compound **4**	250(-3.79)	271(2.92)
14.4 µM ds(AT)$_6$ + 72 µM compound **4**	250(-3.46)	271(2.70)
14.4 µM ds(AT)$_6$ + 72 µM compound **5**	250(-3.22)	271(2.51)
14.4 µM ds(AT)$_6$ + 144 µM compound **5**	250(-2.65)	271(2.16)
14.4 µM ds(AT)$_6$ + 108 µM compound **6**	251(-2.11)	271(2.33)
14.4 µM ds(AT)$_6$ + 216 µM compound **6**	253(-0.33)	271(0.84)
14.4 µM ds(AT)$_6$ + 144 µM compound **7**	249(-1.63)	271(2.72)
14.4 µM ds(AT)$_6$ + 288 µM compound **7**	249(-0.31)	271(0.86)
14.4 µM ds(AT)$_6$ + 108 µM compound **8**	250(-3.28)	271(2.86)
14.4 µM ds(AT)$_6$ + 216 µM compound **8**	250(-3.32)	271(2.90)
14.4 µM ds(AT)$_6$ + 72 µM compound **9**	251(-3.70)	271(2.97)
14.4 µM ds(AT)$_6$ + 144 µM compound **9**	250(-3.78)	271(3.24)

14.4 μM ds(AT)$_6$ + 72 μM compound **10**	250(-3.31)	271(3.21)
14.4 μM ds(AT)$_6$ + 144 μM compound **10**	250(-3.26)	271(3.25)
15.8 μM ds(GC)$_6$	253(-7.10)	284(3.27)
15.8 μM ds(GC)$_6$ + 160 μM compound **1**	254(-4.56)	286(3.69)
15.8 μM ds(GC)$_6$ + 320 μM compound **1**	257(-2.23)	296(4.65)
15.8 μM ds(GC)$_6$ + 400 μM compound **1**	259(-1.49)	296(5.01)
15.8 μM ds(GC)$_6$ + 80 μM compound **2**	253(-6.26)	285(3.52)
15.8 μM ds(GC)$_6$ + 160 μM compound **2**	253(-5.50)	287(3.49)
15.8 μM ds(GC)$_6$ + 320 μM compound **2**	254(-5.20)	290(3.82)
15.8 μM ds(GC)$_6$ + 80 μM compound **6**	254(-5.11)	291(4.16)
15.8 μM ds(GC)$_6$ + 160 μM compound **6**	255(-1.83)	295(6.11)
15.8 μM ds(GC)$_6$ + 320 μM compound **6**	256(0.21)	296(6.69)
15.8 μM ds(GC)$_6$ + 40 μM compound **10**	253(-6.06)	280(4.01)
15.8 μM ds(GC)$_6$ + 80 μM compound **10**	253(-5.77)	281(4.27)
15.8 μM ds(GC)$_6$ + 160 μM compound **10**	253(-5.07)	280(4.34)
8.7 μM ds(GC)$_6$	253(-3.97)	286(1.77)
8.7 μM ds(GC)$_6$ + 90 μM compound **3**	254(-2.68)	287(1.34)
8.7 μM ds(GC)$_6$ + 180 μM compound **3**	255(-2.78)	290(1.58)
8.7 μM ds(GC)$_6$ + 20 μM compound **4**	255(-3.57)	286(1.84)
8.7 μM ds(GC)$_6$ + 40 μM compound **4**	255(-3.35)	287(1.89)
8.7 μM ds(GC)$_6$ + 40 μM compound **5**	255(-3.24)	287(1.75)
8.7 μM ds(GC)$_6$ + 80 μM compound **5**	255(-2.66)	287(1.45)
8.7 μM ds(GC)$_6$ + 90 μM compound **7**	253(-2.47)	289(2.85)
8.7 μM ds(GC)$_6$ + 180 μM compound **7**	256(-0.99)	290(2.16)
8.7 μM ds(GC)$_6$ + 60 μM compound **8**	254(-3.23)	286(1.90)
8.7 μM ds(GC)$_6$ + 120 μM compound **8**	254(-3.18)	286(1.95)
8.7 μM ds(GC)$_6$ + 30 μM compound **9**	254(-3.54)	287(1.80)
8.7 μM ds(GC)$_6$ + 60 μM compound **9**	254(-3.54)	289(1.86)

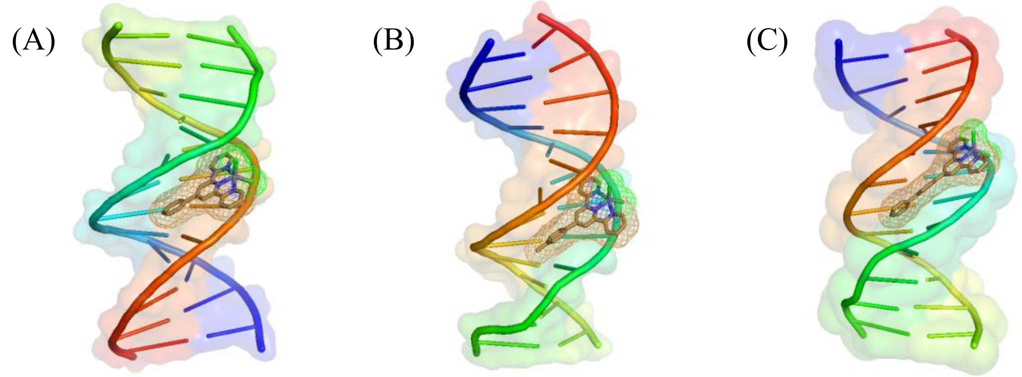

Figure S39 The most favorable orientation of (A): compound **1**, (B): compound **2** and (C): compound **4** bound with the minor groove of the CT-DNA (PDB ID: 1BNA).

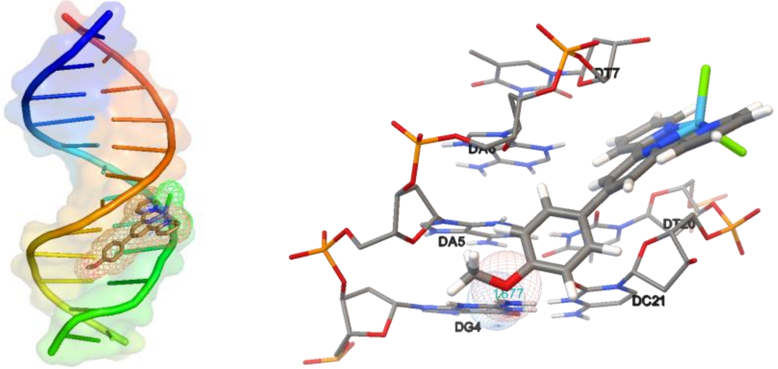

Figure S40 The most favorable orientation of compound **3** bound with the minor groove of the B-DNA (PDB ID: 1BNA). The formed hydrogen bond and distance have been marked.

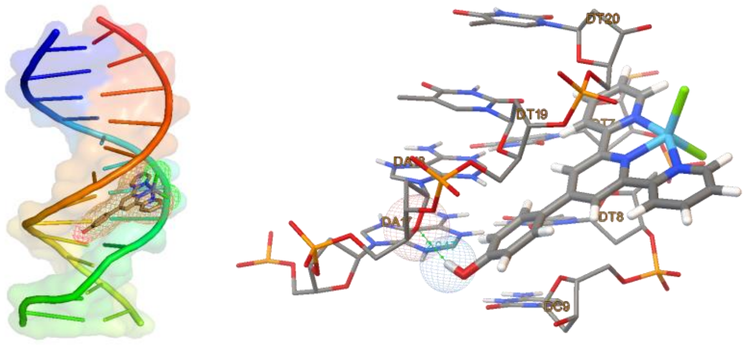

Figure S41 The most favorable orientation of compound **6** bound with the minor groove of the B-DNA (PDB ID: 1BNA). The formed hydrogen bond and distance have been marked.

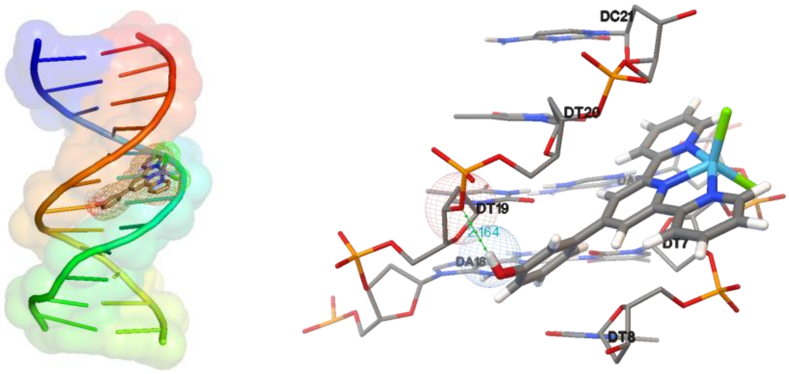

Figure S42 The most favorable orientation of compound **7** bound with the minor groove of the B-DNA (PDB ID: 1BNA). The formed hydrogen bond and distance have been marked.

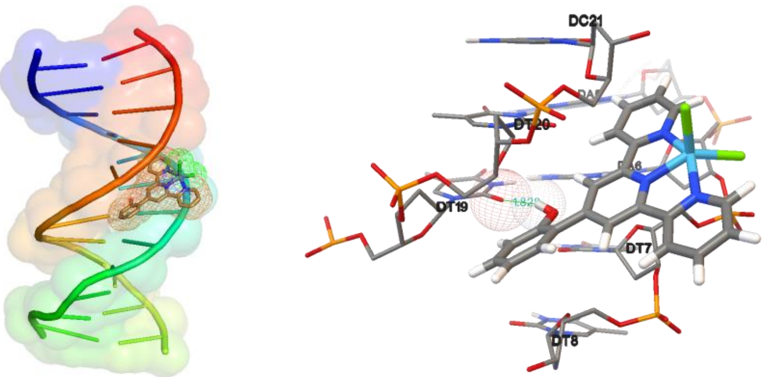

Figure S43 The most favorable orientation of compound **8** bound with the minor groove of the B-DNA (PDB ID: 1BNA). The formed hydrogen bond and distance have been marked.

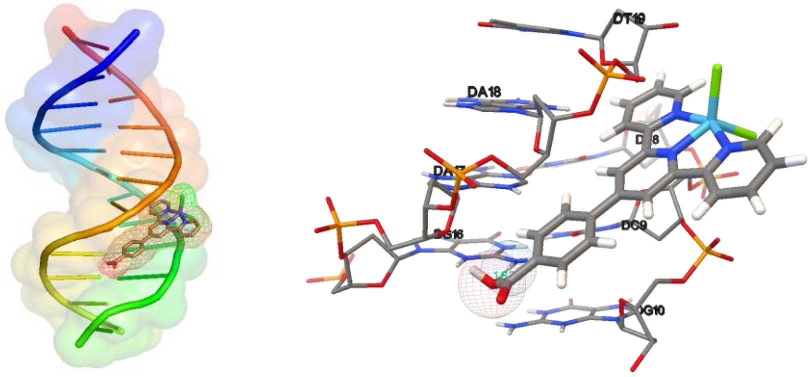

Figure S44 The most favorable orientation of compound **9** bound with the minor groove of the B-DNA (PDB ID: 1BNA). The formed hydrogen bond and distance have been marked.

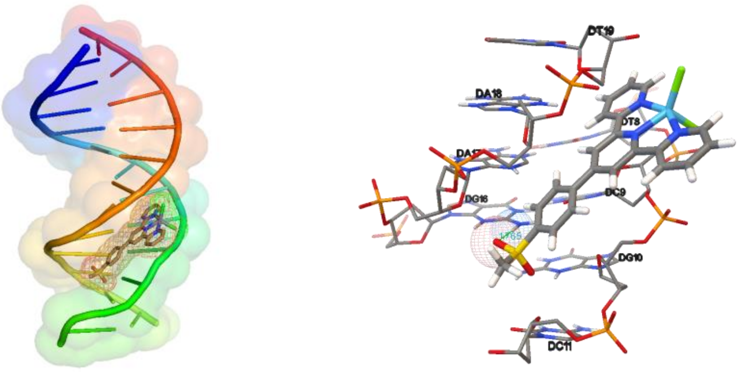

Figure S45 The most favorable orientation of compound **10** bound with the minor groove of the B-DNA (PDB ID: 1BNA). The formed hydrogen bond and distance have been marked.

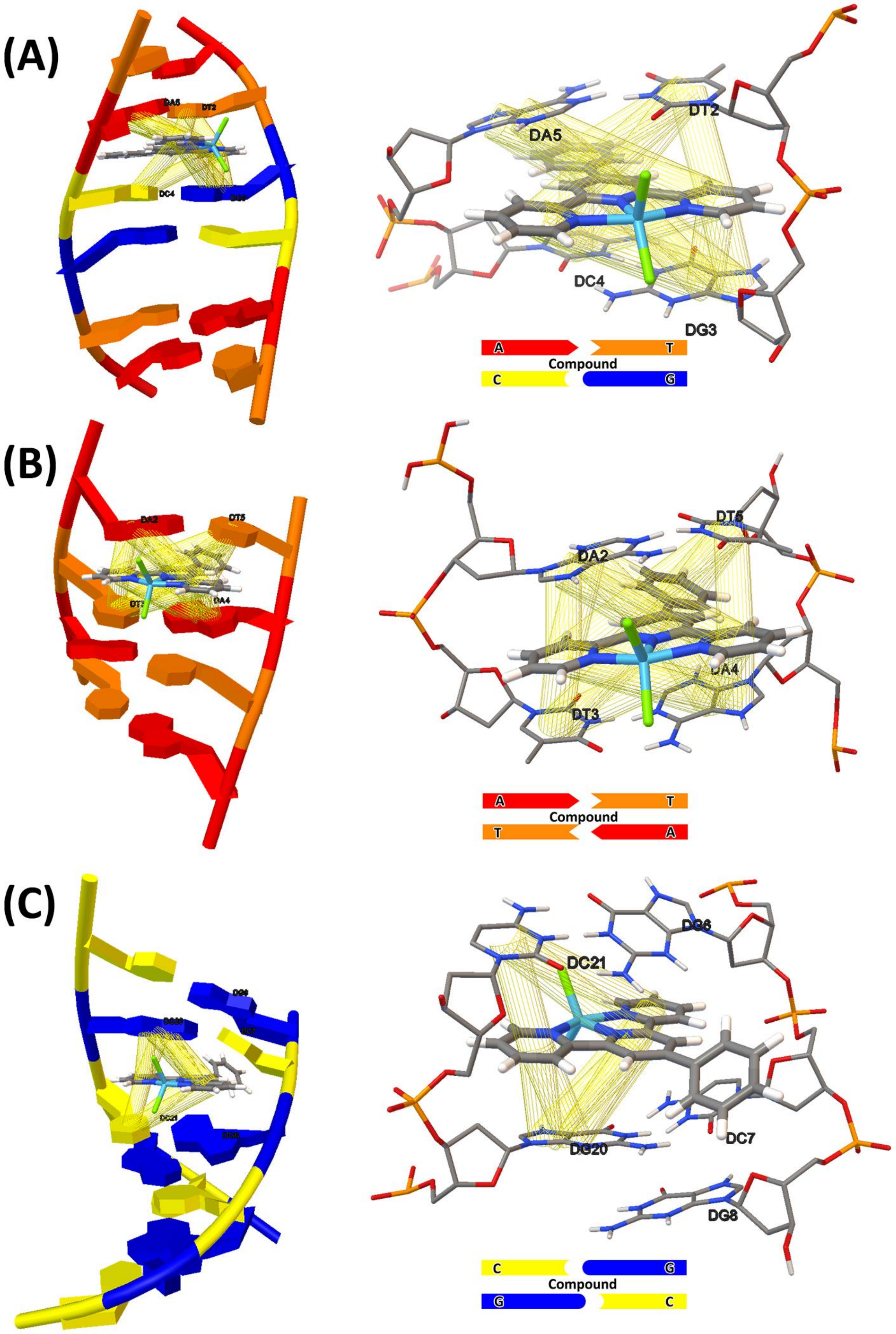

Figure S46 View of the energy minimized docked poses of compound **1** with (A) ds(ATGCAT)$_2$ (PDB ID: 4JD8), (B) ds(ATAT)$_2$ (PDB ID: 2DA8M), and (C) ds(CGCGCG)$_2$ (PDB ID: 2ROUM).

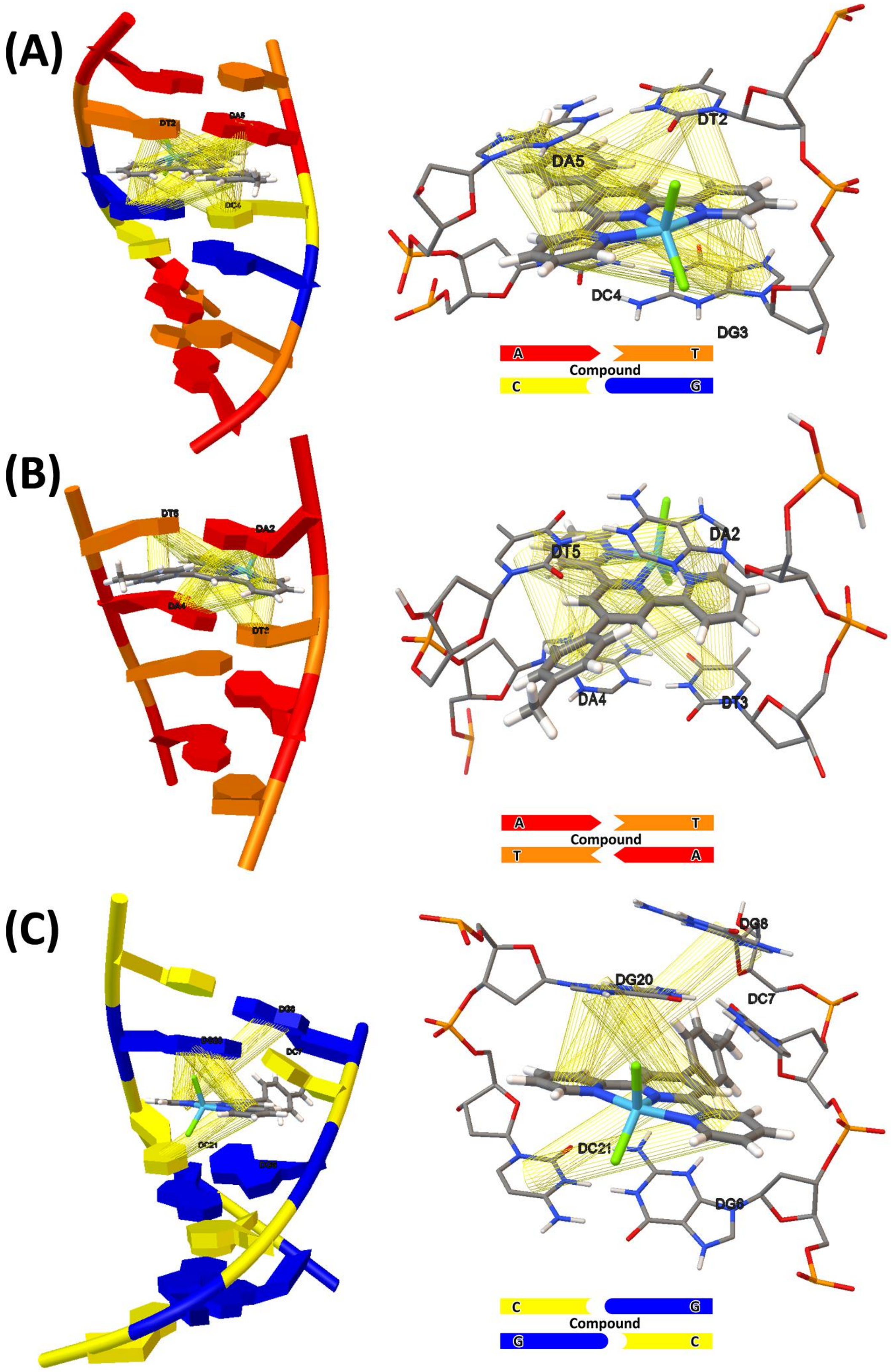

Figure S47 View of the energy minimized docked poses of compound **2** with (A) ds(ATGCAT)$_2$ (PDB ID: 4JD8), (B) ds(ATAT)$_2$ (PDB ID: 2DA8M), and (C) ds(CGCGCG)$_2$ (PDB ID: 2ROUM).

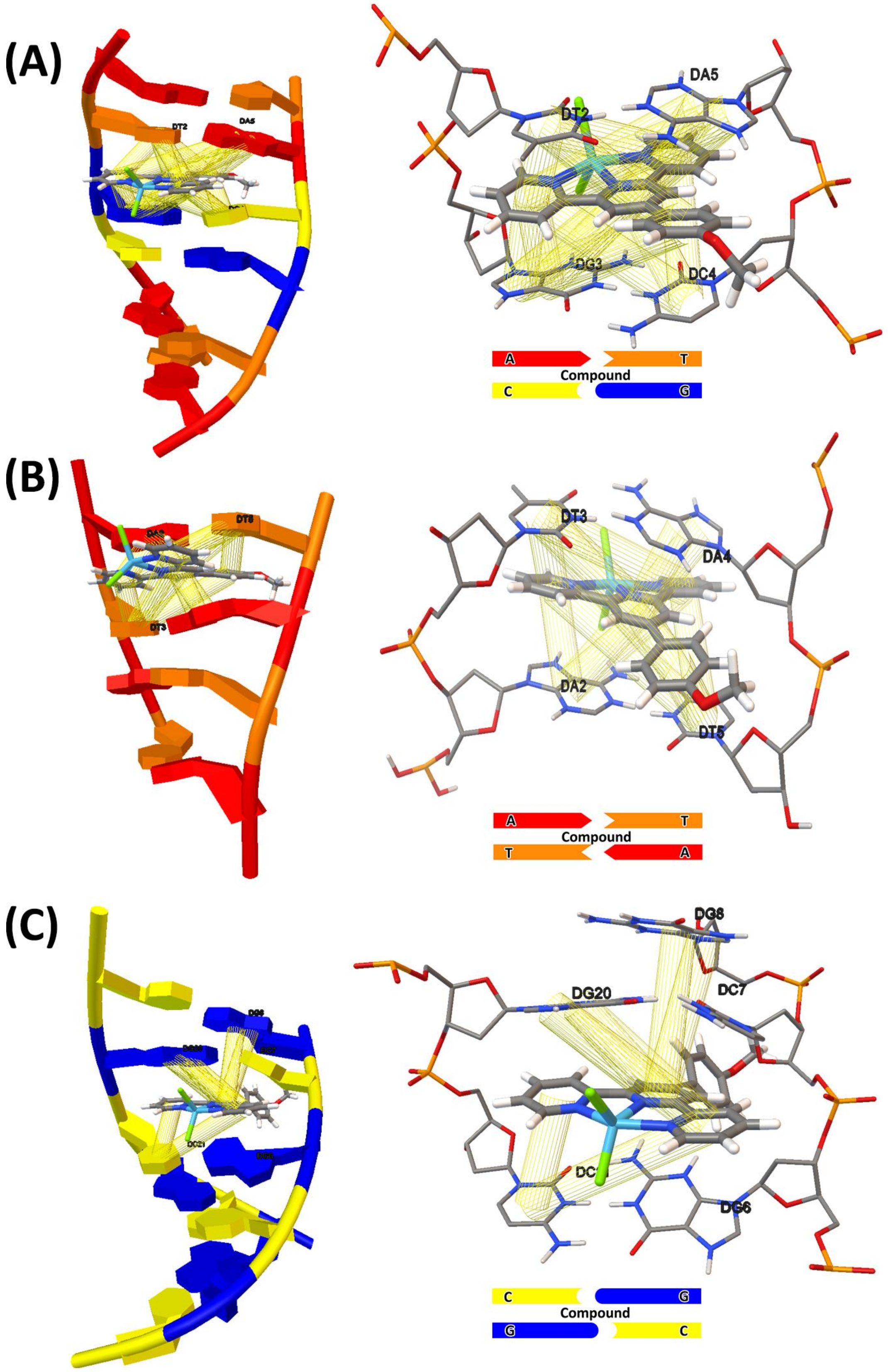

Figure S48 View of the energy minimized docked poses of compound **3** with (A) ds(ATGCAT)$_2$ (PDB ID: 4JD8), (B) ds(ATAT)$_2$ (PDB ID: 2DA8M), and (C) ds(CGCGCG)$_2$ (PDB ID: 2ROUM).

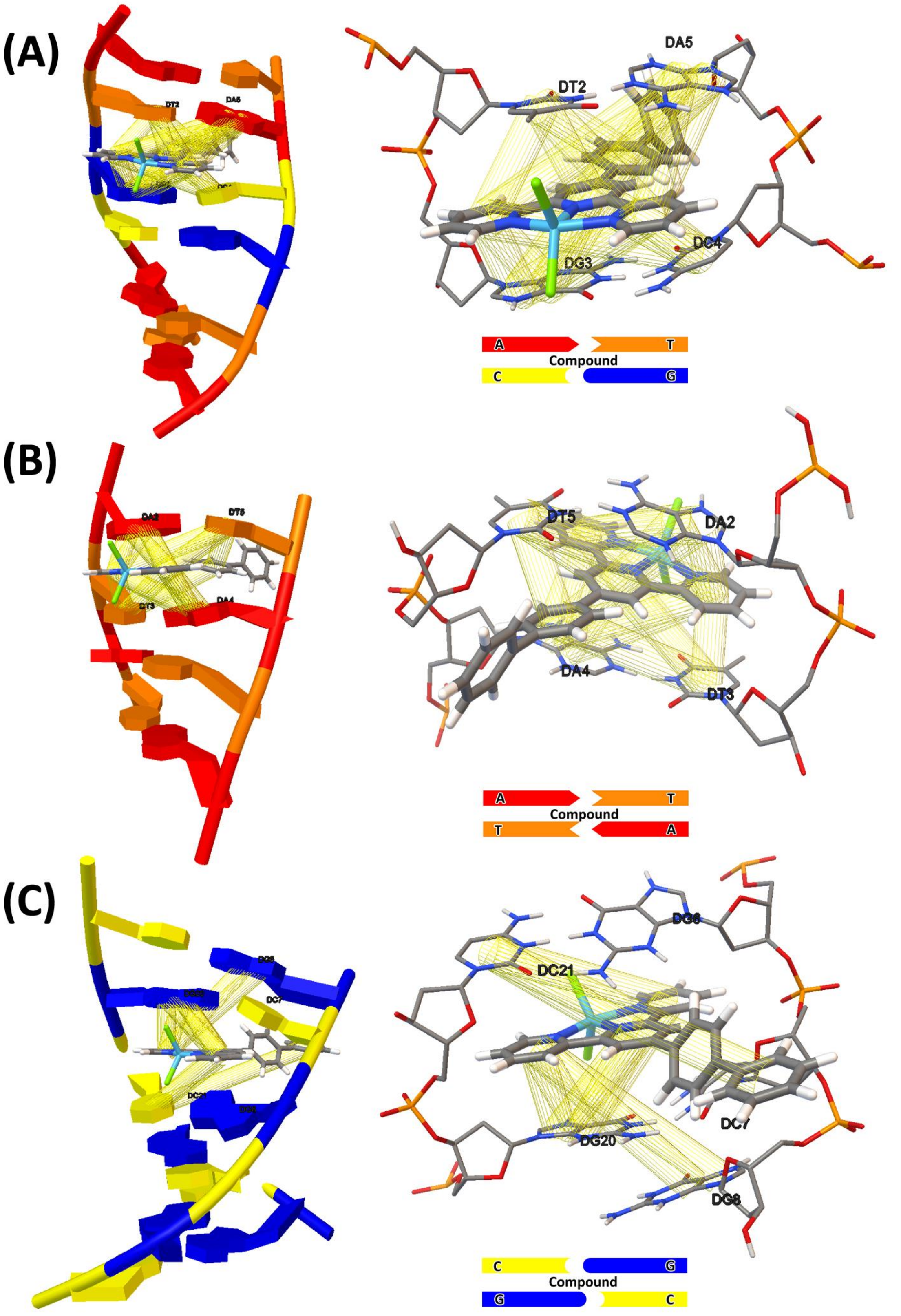

Figure S49 View of the energy minimized docked poses of compound **4** with (A) ds(ATGCAT)$_2$ (PDB ID: 4JD8), (B) ds(ATAT)$_2$ (PDB ID: 2DA8M), and (C) ds(CGCGCG)$_2$ (PDB ID: 2ROUM).

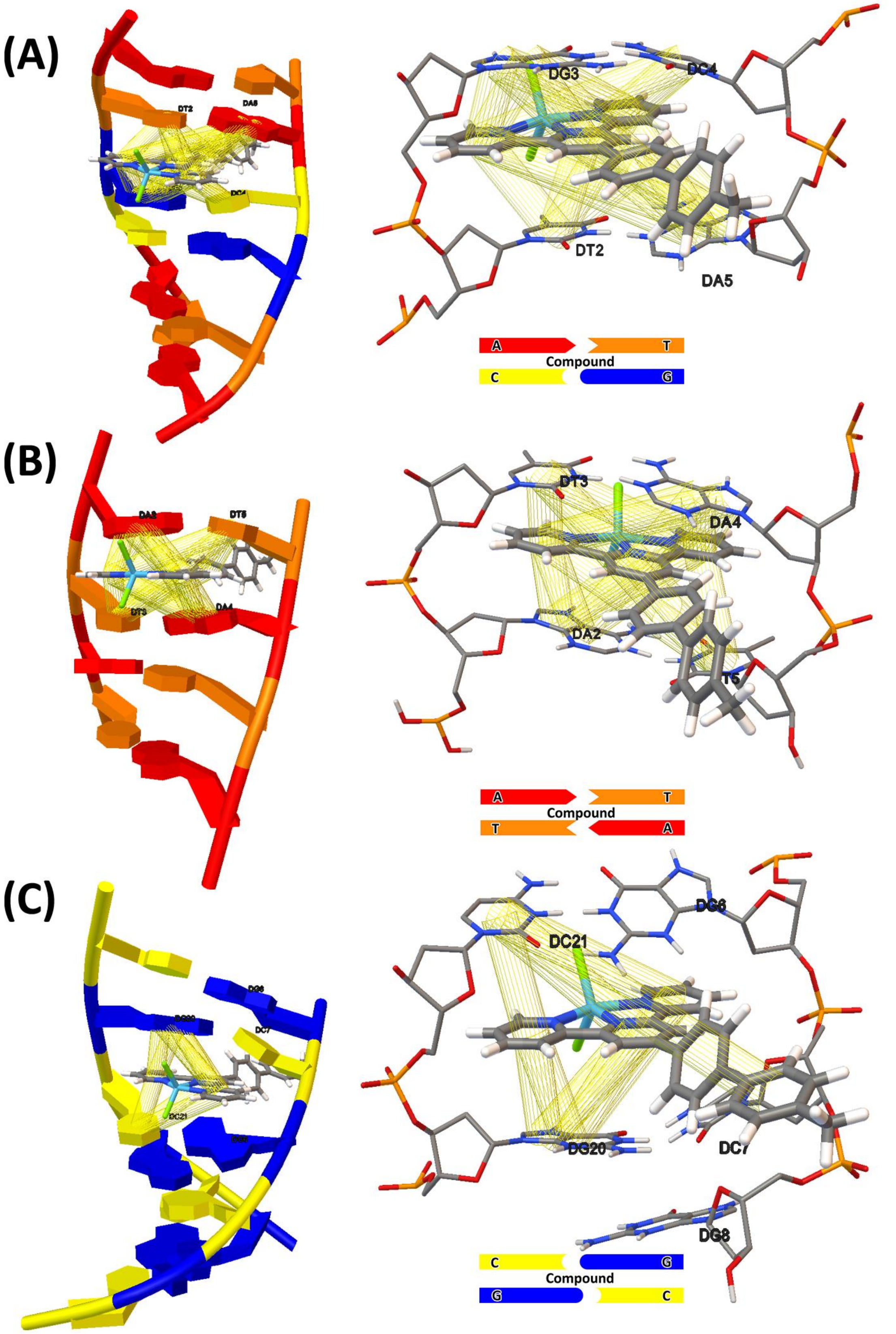

Figure S50 View of the energy minimized docked poses of compound **5** with (A) ds(ATGCAT)$_2$ (PDB ID: 4JD8), (B) ds(ATAT)$_2$ (PDB ID: 2DA8M), and (C) ds(CGCGCG)$_2$ (PDB ID: 2ROUM).

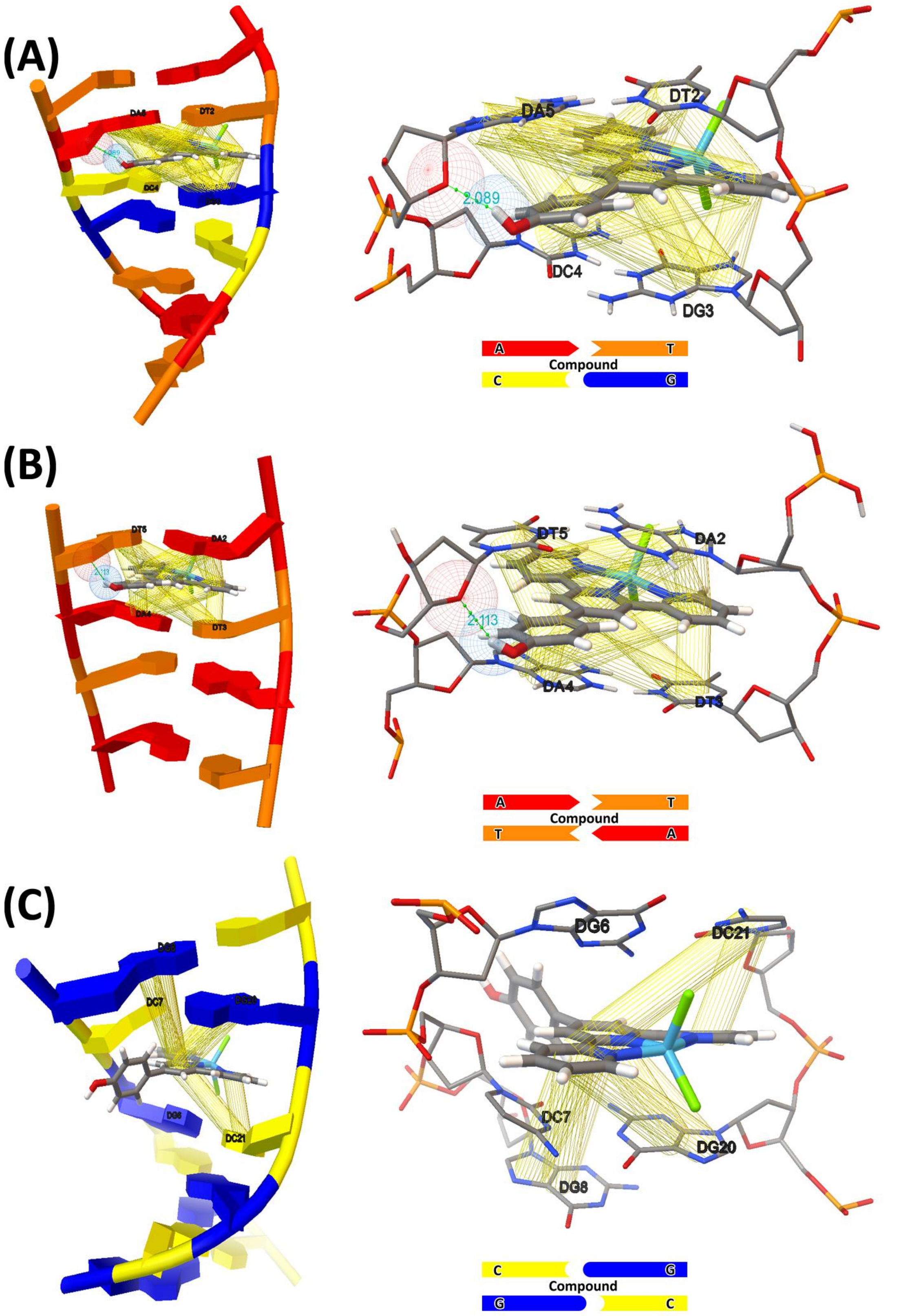

Figure S51 View of the energy minimized docked poses of compound **6** with (A) ds(ATGCAT)$_2$ (PDB ID: 4JD8), (B) ds(ATAT)$_2$ (PDB ID: 2DA8M), and (C) ds(CGCGCG)$_2$ (PDB ID: 2ROUM).

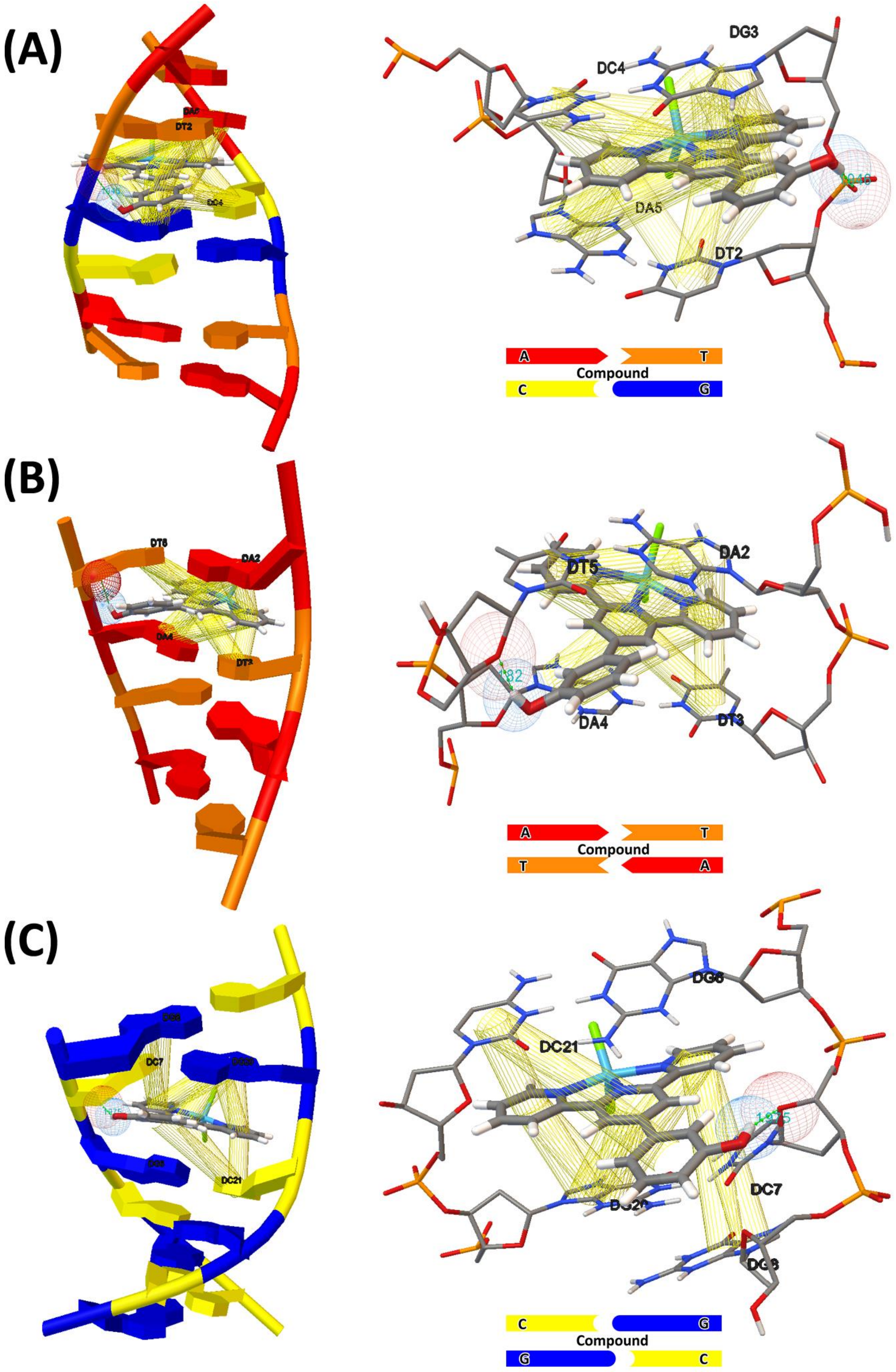

Figure S52 View of the energy minimized docked poses of compound **7** with (A) ds(ATGCAT)$_2$ (PDB ID: 4JD8), (B) ds(ATAT)$_2$ (PDB ID: 2DA8M), and (C) ds(CGCGCG)$_2$ (PDB ID: 2ROUM).

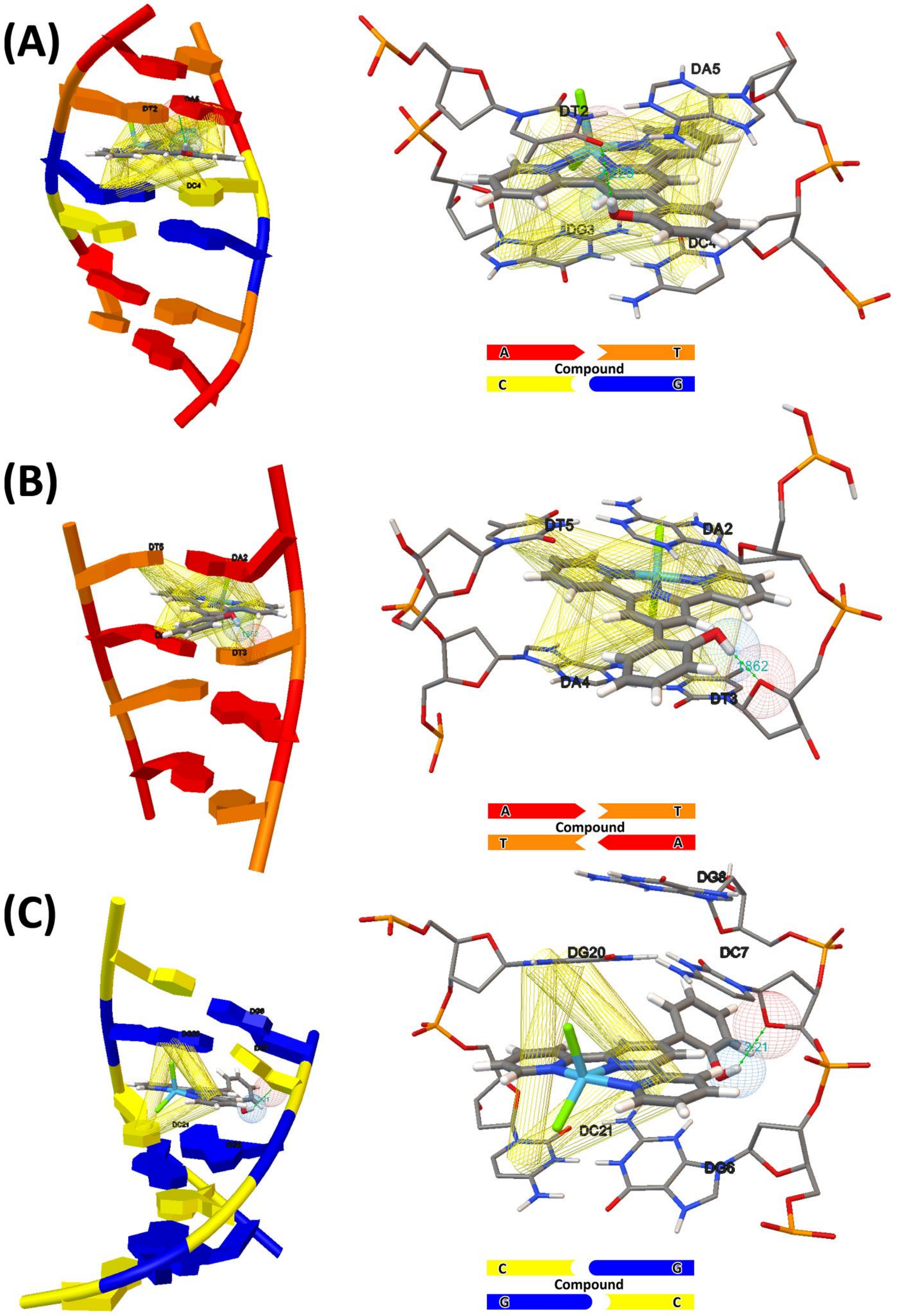

Figure S53 View of the energy minimized docked poses of compound **8** with (A) ds(ATGCAT)$_2$ (PDB ID: 4JD8), (B) ds(ATAT)$_2$ (PDB ID: 2DA8M), and (C) ds(CGCGCG)$_2$ (PDB ID: 2ROUM).

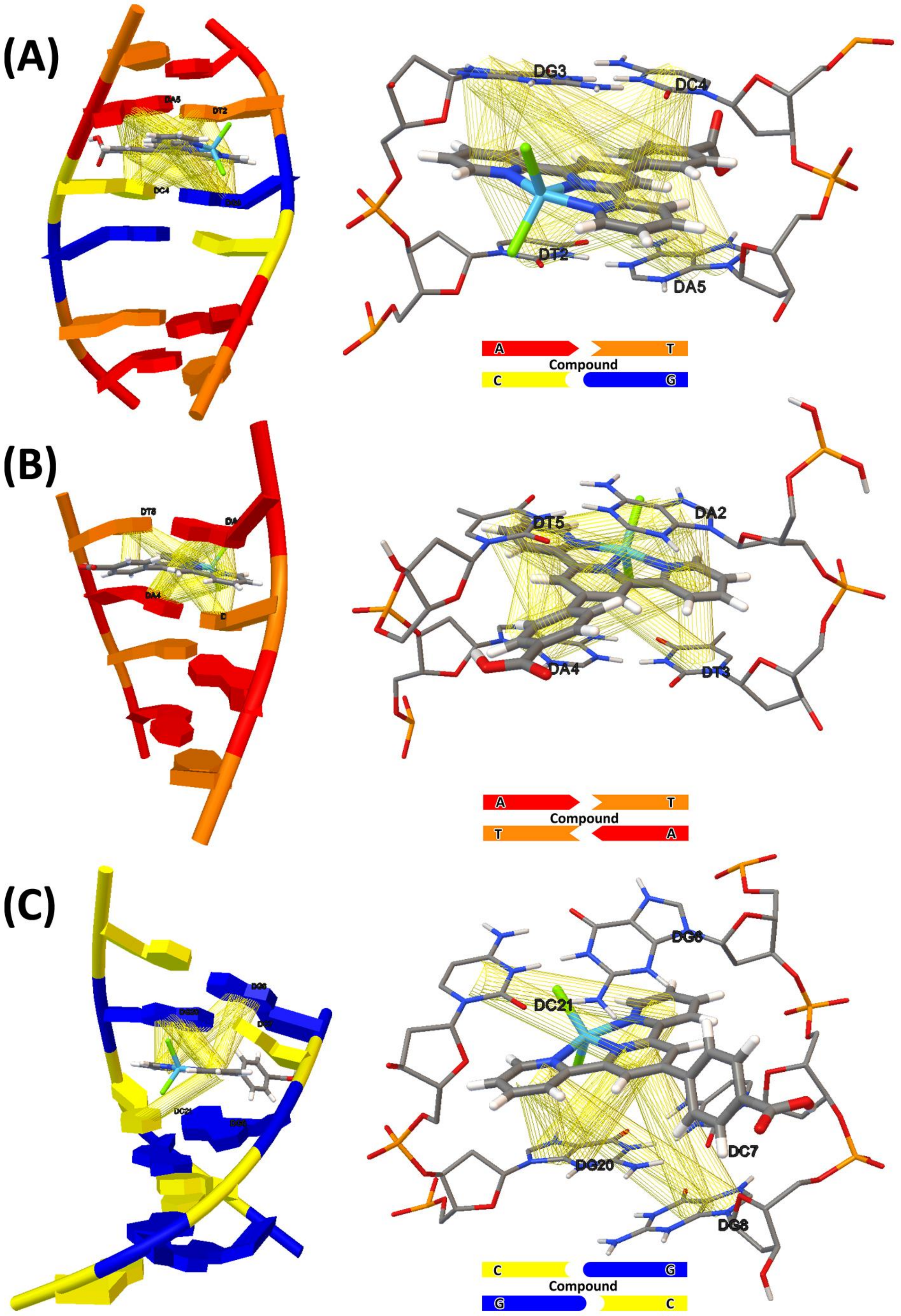

Figure S54 View of the energy minimized docked poses of compound **9** with (A) ds(ATGCAT)$_2$ (PDB ID: 4JD8), (B) ds(ATAT)$_2$ (PDB ID: 2DA8M), and (C) ds(CGCGCG)$_2$ (PDB ID: 2ROUM).
